# ﻿A review of Nearctic *Lathrobium* (Coleoptera, Staphylinidae), with revision and descriptions of new flightless species from the mountains of the southeastern U.S.

**DOI:** 10.3897/zookeys.1198.118355

**Published:** 2024-04-25

**Authors:** Adam Haberski, Michael S. Caterino

**Affiliations:** 1 Department of Plant & Environmental Sciences, Clemson University, Clemson, SC 29634-0310, USA Clemson University Clemson United States of America

**Keywords:** Appalachian Mountains, cryptic species, larvae, molecular phylogeny, morphology, Paederinae, sky-islands, species delimitation

## Abstract

Species of the genus *Lathrobium* Gravenhorst (Coleoptera: Staphylinidae: Paederinae) from North America north of Mexico are reviewed and 41 species are recognized. Morphology and mitochondrial COI sequence data were used to guide species designations in three flightless lineages endemic to the southern Appalachian Mountains, a biologically diverse region known for cryptic diversity. Using a combination of phylogeny, algorithm-based species delimitation analyses, and genitalic morphology, five new cryptic species are described and possible biogeographic scenarios for their speciation hypothesized: *L.balsamense* Haberski & Caterino, **sp. nov.**, *L.camplyacra* Haberski & Caterino, **sp. nov.**, *L.islae* Haberski & Caterino, **sp. nov.**, *L.lividum* Haberski & Caterino, **sp. nov.**, *L.smokiense* Haberski & Caterino, **sp. nov.** Five additional species are described: *L.absconditum* Haberski & Caterino, **sp. nov.**, *L.hardeni* Haberski & Caterino, **sp. nov.**, *L.lapidum* Haberski & Caterino, **sp. nov.**, *L.solum* Haberski & Caterino, **sp. nov.**, and *L.thompsonorum* Haberski & Caterino, **sp. nov.** Two species are transferred from *Lathrobium* to *Pseudolathra* Casey: *Pseudolathraparcum* (LeConte, 1880), **comb. nov.** and *Pseudolathratexana* (Casey, 1905), **comb. nov.** Twenty-six names are reduced to synonymy. Lectotypes are designated for 47 species. Larvae are described where known, and characters of possible diagnostic value are summarized. Species diagnoses, distributions, illustrations of male and female genitalia, and a key to *Lathrobium* species known from the Nearctic region (including several introduced species) are provided.

## ﻿Introduction

The Holarctic genus *Lathrobium* Gravenhorst, 1802 is the most speciose genus of Paederinae with over 650 species (Assing 2019). The Nearctic fauna is relatively species poor and incompletely known, with 64 described species and no new descriptions in more than 100 years (Notman 1921). A revision was attempted by Casey (1905), but it caused more confusion than clarity because he created copious synonyms. Although the generic limits have since been clarified (Blackwelder 1939; Assing and Schülke 2012; Żyła et al. 2020; Staniec and Bordoni 2022), identification of species remains nearly impossible without referring to the type specimens, because external morphology is highly conserved, and the only comprehensive key is outdated and based on dubious characters (Casey 1905). Regional keys exist for northeastern North America (Downie and Arnett 1995) and Indiana (Blatchley 1910), but both are derived from Casey (1905) and share its shortcomings.

Much of *Lathrobium* diversity can be attributed to flightless, short-range endemics that are restricted to cryophilic microhabitats such as mountains, caves, and deep soil layers. They are numerous in mountain ranges where complex topography produces clusters of allopatric endemics, such as 48 species endemic to the Himalayas and 29 endemic to the Apennine Mountains of Italy (Assing, 2012; Bordoni, 2018). Among these, species that are depigmented, microphthalmous, and generally subterranean are placed in the subgenera *Abletobium* Casey (Nearctic) and *Glyptomerus* Müller (Palearctic). Both subgenera lack clear synapomorphies beyond their gestalt, which is convergent among hypogean taxa, and their boundaries are unclear. Leaf litter and soil layers in the southeastern United States are just now being fully explored and have already revealed new flightless *Lathrobium* diversity, including new species of *Abletobium* and high elevation endemics in the southern Appalachian Mountains. They present an opportunity to reexamine the relationship between *Abletobium* and *Glyptomerus*, and to explore patterns of montane diversification.

Recent phylogeographic studies of southern Appalachian arthropods have repeatedly uncovered cryptic diversity among seemingly widespread flightless species (Gusarov 2002; Miller and Wheeler 2005; Carlton 2008; Ferro and Carlton 2010; Caterino and Vásquez-Vélez 2017; Caterino and Langton-Myers 2019; Caterino 2022; Caterino and Recuero 2024; Caterino and Harden 2024). A cursory examination of Appalachian *Lathrobium* genitalia suggested there might be cryptic diversity among three lineages endemic to southern Appalachian “sky-islands.” Two of these lineages are endemic to a rare bryophyte mat microhabitat in the endangered red spruce-Fraser fir (*Picearubens* and *Abiesfraseri*) forests, a habitat they share with the federally protected spruce-fir moss spider, *Microhexuramontivaga* Crosby & Bishop, 1925. Properly delimiting species boundaries and distributions could therefore have implications for conservation and provide additional motivation to protect these sensitive habitats.

To address current taxonomic deficiencies and document new diversity, we review the Nearctic species of *Lathrobium*, with emphasis on the flightless species of the southern Appalachian Mountains. We use an integrative approach that combines morphology, molecular phylogeny, and algorithmic species delimitation to guide species designations and test the hypothesis of cryptic speciation in three high elevation Appalachian lineages. We also describe five new microphthalmous species from lower elevations and the larvae of five species. Larval morphology is compared with that of other known larvae to ascertain characters of possible diagnostic value.

## ﻿Materials and methods

We follow the generic concepts of Assing and Schülke (2012), which treat *Lathrobium* in a restricted sense and recognize *Lobrathium* Mulsant and Rey, *Pseudolathra* Casey, and *Tetartopeus* Czwalina as distinct genera. Species are described under a phylogenetic species concept, in which species are “the smallest aggregation of populations or lineages diagnosable by a unique combination of character states” (Wheeler and Meier 2000). We have avoided the use of subspecies as the biological requirements for such designation (e.g., the ability of populations to interbreed) are undefined, and instead treat consistent lineages exhibiting morphological or molecular variation as full species.

An initial morphological investigation revealed three lineages that might contain cryptic diversity, *Lathrobiumcarolinae* (Casey, 1905), and the yet undescribed lineages “*Lathrobiumsmokiense*,” and “*Lathrobiumislae*”. They belong to the subgenera *Apteralium*, *Abletobium*, and *Lathrobium* s. str., respectively. Each lineage contains two allopatric morphotypes that are indistinguishable externally but differ in genitalic morphology. We use morphology, phylogenetic inference, Assemble Species by Automatic Partitioning (ASAP), and Multi-rate Poisson tree processes (mPTP) to guide species delimitation in these lineages. ASAP and mPTP are complementary species discovery methods. ASAP uses pairwise distances to partition samples into putative species (Puillandre et al. 2021), but unlike previous “barcode gap” methods, ASAP tests and ranks multiple partition thresholds. mPTP is a single-locus, tree-based coalescence method that uses a maximum-likelihood criterion to delimit species based on branch lengths (Kapli et al. 2017).

A revision of *Lathrobium* was begun by L. Watrous (formerly of the Field Museum, Chicago, Illinois, USA) but never published. He dissected all of the type specimens and first hypothesized the synonymies recognized herein.

### ﻿Molecular methods

A larger-scale molecular phylogeny of Nearctic *Lathrobium* was conducted in tandem with this study and will be published separately. It includes a more detailed explanation of the DNA extraction and amplification processes used. Briefly, whole genome DNA was non-destructively extracted from freshly collected specimens and from museum specimens fixed in 100% ethanol using the GeneJET Genomic DNA Purification Kit (Thermo Fisher Scientific Inc., Waltham, MA, USA). The abdomen was separated from the thorax and both halves were digested with lysis buffer and proteinase K. Following extraction, cleared specimens were dissected, point mounted, and assigned unique Clemson University Arthropod Collection (**CUAC**) numbers.

We amplified and sequenced a 658bp fragment of the mitochondrial gene cytochrome oxidase I (COI) using standard primers (HCO1490+LCO2198; Folmer et al. 1994) and following the amplification profile of Chatzimanolis et al. (2010). Sanger sequencing was performed by Psomagen (Rockville, MD) and all fragments were sequenced in both directions.

Pairwise alignment of reads was performed in GENEIOUS v. 8.1.8. Primer sequences were trimmed, and alignments were visually inspected for quality. Next, we used the EXPASY webtool (Artimo et al. 2012) to find a read frame free of stop codons. Multiple sequence alignment was then performed with MAFFT (Katoh et al. 2019) using the Muscle algorithm and default settings.

We performed a maximum-likelihood analysis with IQ-TREE (Minh et al. 2020) on 50 *Lathrobium* sequences. Our samples included all three cryptic lineages and five other flightless species. We chose *Tetartopeustetricum* Casey, 1905, *Medonicarus* Caterino, 2023, and *Astenus* sp. as outgroups. Sequence data were initially partitioned by codon position and then we allowed candidate partitions to be merged during model estimation to avoid over-parameterization of the model. The best-fitting model was selected automatically with free rate heterogeneity (+R). We performed 1000 ultrafast bootstrap analyses to determine branch support values.

For ASAP analysis, we chose Kimura 2-parameter (K2P) distances and report results for the best partitioning schemes. We used the maximum likelihood tree as the input tree for mPTP analysis.

### ﻿Morphology

Adult terminology follows Watrous (1980), and larval terminology follows Staniec and Bordoni (2022). Adults were examined with a Leica M80 stereomicroscope, or equivalent, and measurements were taken with a Leica M80 eyepiece micrometer. Larvae were suspended in a drop of hand sanitizer on a recessed slide and examined with a Zeiss Axioscope. Most specimens were cleared in conjunction with DNA extraction. After tissue digestion, abdominal segment VIII and the genitalia were removed. Additional specimens were dissected by relaxing in boiling water, removing the genitalia, and clearing them in 85% lactic acid warmed to 110 °C for 20 min (Blahnik et al. 2007).

Digital illustrations were created by importing hand sketches and photographs into Affinity Designer (Serif Europe Ltd, Nottingham, UK). Multiple specimens were sometimes referenced to create a single illustration that displayed all characters. Dashed lines are used to indicate structures that are obscured from view, such as the female subgenital plate and spines of the internal sac of the aedeagus. Distribution data were compiled from specimen label data and the literature. New state or province records are in bold.

We examined material from the following institutions:

**CMNH** Carnegie Museum of Natural History

**CNC** Canadian National Collection

**CUAC** Clemson University Arthropod Collection

**GSNP** Great Smoky Mountains National Park Collection

**LSAM** Louisiana State Arthropod Museum

**MCZC** Museum of Comparative Zoology

**NBC** Nicolas Bédard Private Collection

**UAM** University of Alaska Museum

**UNHC** University of New Hampshire

**USNM** United States National Museum

Holotypes were deposited in the **FMNH**, Chicago, Illinois, USA. Paratypes and additional specimens were vouchered in the **CUAC**, Great Smoky Mountains National Park, Gatlinburg, Tennessee (**GSNP**), and Virginia Museum of Natural History, Martinsville, Virginia (**VMNH**).

We did not examine material from five species because the types were inaccessible at the time, and we could not find authoritatively identified specimens in available collections. We could not locate Casey’s specimens of *Lathrobiumsubgracile* Casey, 1905 in the USNM collection. The holotype of *Lathrobiumpuncticolle* Kirby, 1837 is at the Natural History Museum, London, United Kingdom, and we did not attempt to borrow it. Time and access constraints during the COVID-19 pandemic prevented us from examining the types *Lathrobiuminsanum* Blatchley, 1910 in the Purdue Entomological Research Collection and *Lathrobiumlintneri* Notman, 1921 and *Lathrobiumtenebrosum* Notman, 1921, reportedly in the Staten Island Institute of Arts and Sciences collection. These species were excluded from the key. Similarly, we were unable to illustrate some characters for *Lathrobiumcrurale* (Casey, 1905) and *Lathrobiumlineatocolle* Scriba, 1859 due to a lack of authoritatively identified specimens.

**Figure 1.**
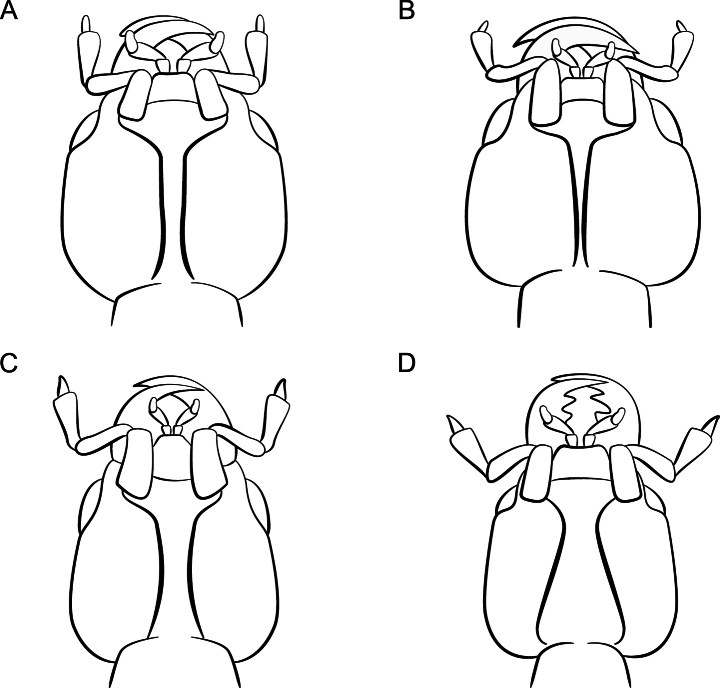
*Lathrobium* gular sutures **A***L.simile***B***L.amplipenne***C***L.islae***D***L.debile*.

External morphology is highly conserved, but species are easily identified by their sexual characters, especially the aedeagus and male abdominal sternite VIII. Comparing the terminalia to illustrations is essential for accurate identifications, so dissection is strongly encouraged. The few somatic characters of diagnostic value include the relative dimensions of the head, pronotum, and elytra, the shape of the antennomeres, the shape of the gular sutures (Fig. 1), and the size of the eyes.

Measurements were taken from uncleared adults and cleared larvae. References to color were based on uncleared specimens. Head length was measured at midline from the anterior margin of the frons to the anterior edge of the neck constriction. Pronotal length was measured along the midline. Elytral length was measured from the anterior angle, or “shoulder”, to the apex. Head and pronotum widths were measured at their widest points, and elytra width was measured across both elytra at the widest point. An estimate of total body length is given, but forebody length (**FL**), a surrogate unaffected by the expansion or contraction of intersegmental membranes, was calculated by adding head, pronotum, and elytra length. Eye width was measured in lateral view from the anterior-most to posterior-most point of the eye. Larval body length was measured from the anterior margin of the nasale to the posterior edge of the last abdominal segment (excluding the urogomphi). The relative lengths of larval antennomeres and palpomeres are presented as ratios, beginning with the basal article.

**Figure 2.**
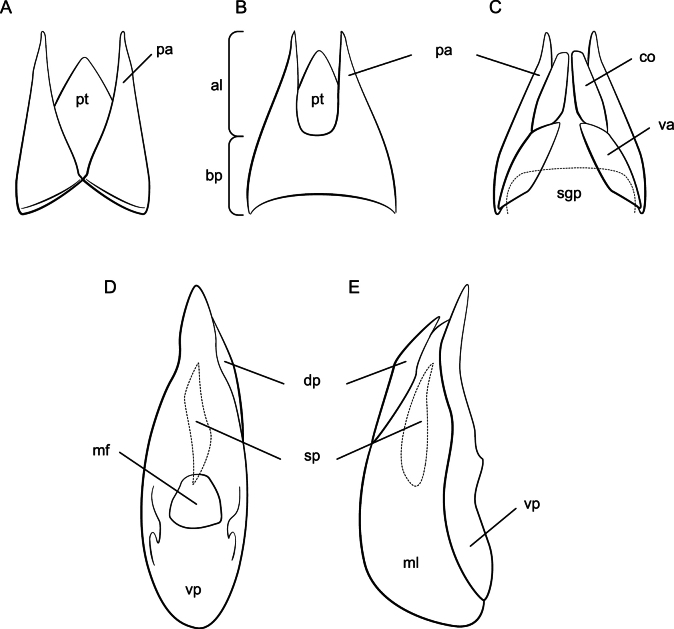
Genitalia morphology **A** female paraprocts divided **B** female paraprocts undivided **C** female terminalia, ventral **D** aedeagus, ventral **E** aedeagus, lateral. Abbreviations: al – apical lobe of paraproct, bp – basal portion of paraproct, co – coxite, dp – dorsal plate, mf – median foreman, ml – median lobe, pa – paraprocts, pt – proctiger, sgp – subgenital plate, sp – spine of the internal sac, va – valvifer, vp – ventral process.

The side of the aedeagus with the median foramen is arbitrarily designated as “ventral” for convenience, although it is positioned laterally in life. The median lobe is divided into a lightly sclerotized basal portion and an elongate and well-sclerotized ventral process, although shape and degree of sclerotization varies. The female sternite VIII occurs in two shapes, conical with sides convex their entire length or oblong with sides concave in the apical 1/3. The female tergite IX (= paraprocts) can be either divided its entire length (Fig. 2A) or continuous in the basal portion. If undivided, the apices can be longer (Fig. 2B) or shorter (Fig. 2C) than the basal portion.

Several characters used in previous keys are not reliable for diagnosis. Elytra length varies in some wing-dimorphic species, being longer than the pronotum in winged individuals and subequal in non-winged individuals. Elytra length does not vary in micropterous species with elytra shorter than the pronotum. Elytra color is likewise variable, with some individuals possessing bicolored elytra (black anteriorly and reddish posteriorly) but others with elytra entirely black.

## ﻿Results

### ﻿Phylogenetics

**Figure 3.**
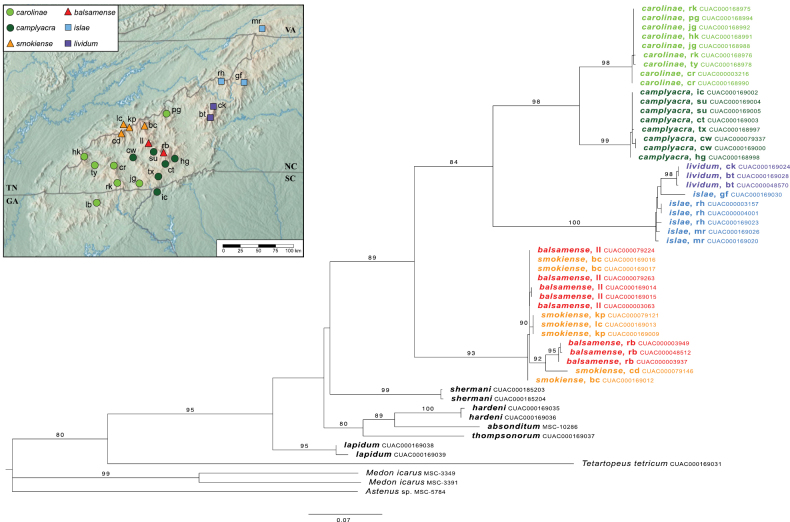
COI maximum-likelihood majority-rule consensus tree of flightless *Lathrobium*. Abbreviations: (bc) Big Cataloochee Mtn; (bt) Big Tom; (cd) Clingmans Dome; (ck) Celo Knob; (cr) Copper Ridge Bald; (cw) Cowee Bald; (gf) Grandfather Mtn; (hg) Horse Cove Gap; (hk) Huckleberry Knob; (ic) Indian Camp Creek; (jg) Jones Gap; (kp) Mt. Kephart; (lb) Little Bald; (lc) Mt. LeConte; (ll) Mt. Lyn Lowry; (mr) Mt. Rogers; (pg) Pisgah National Forest; (rb) Richland Balsam Mtn; (rh) Roan High Knob; (rk) Riley Knob; (su) Sugarloaf Mtn; (tx) Toxaway Mtn; (ty) Teyahalee Bald.

Our final COI alignment contained 221 parsimony-informative sites, 398 constant sites, 39 singleton sites, and 0.14% missing data. Automatic model estimation selected a two-partition scheme fitting the TN+F+I substitution model to the first and second codon positions and the TPM3+F+G4 model to the third codon position. The maximum-likelihood consensus tree was fully resolved, except for a polytomy among the outgroups *Astenus* and *Tetartopeus*, and had good branch support within *Lathrobium* (Fig. 3). Relationships among species at deeper levels are specious due to the globally sparse taxon sampling.

**Figure 4.**
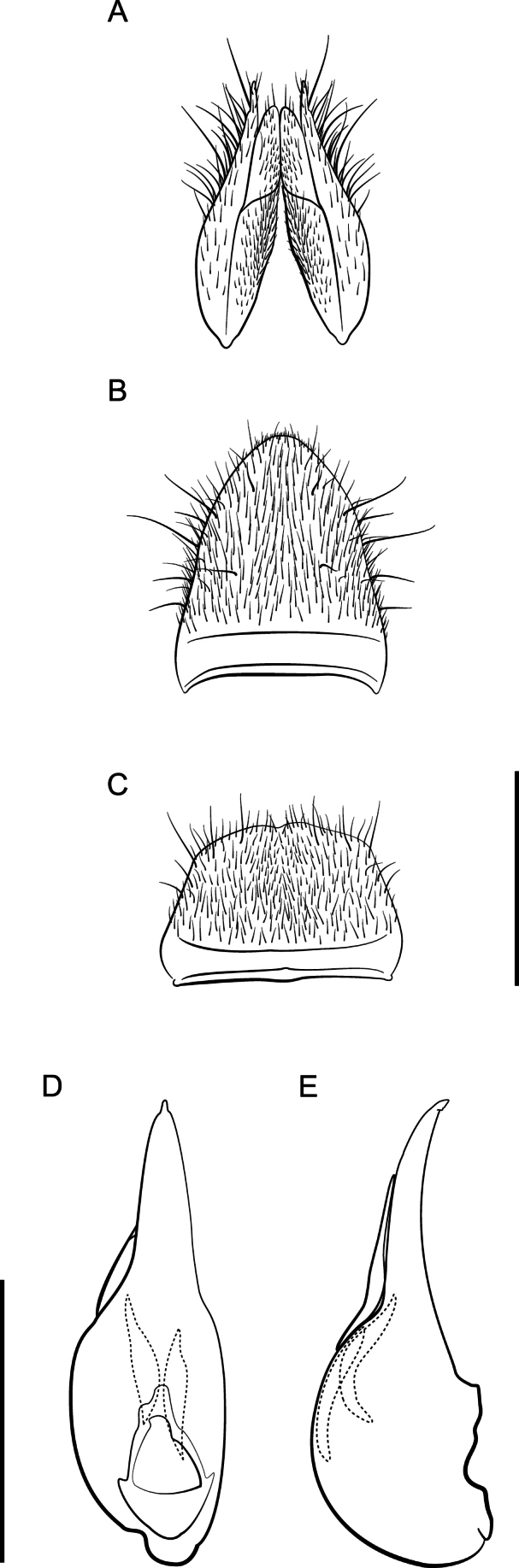
*Lathrobiumcarolinae***A** female terminalia **B** female sternite VIII **C** male sternite VIII **D** aedeagus in ventral view **E** aedeagus in lateral view. Scale bars: 1 mm.

**Figure 5.**
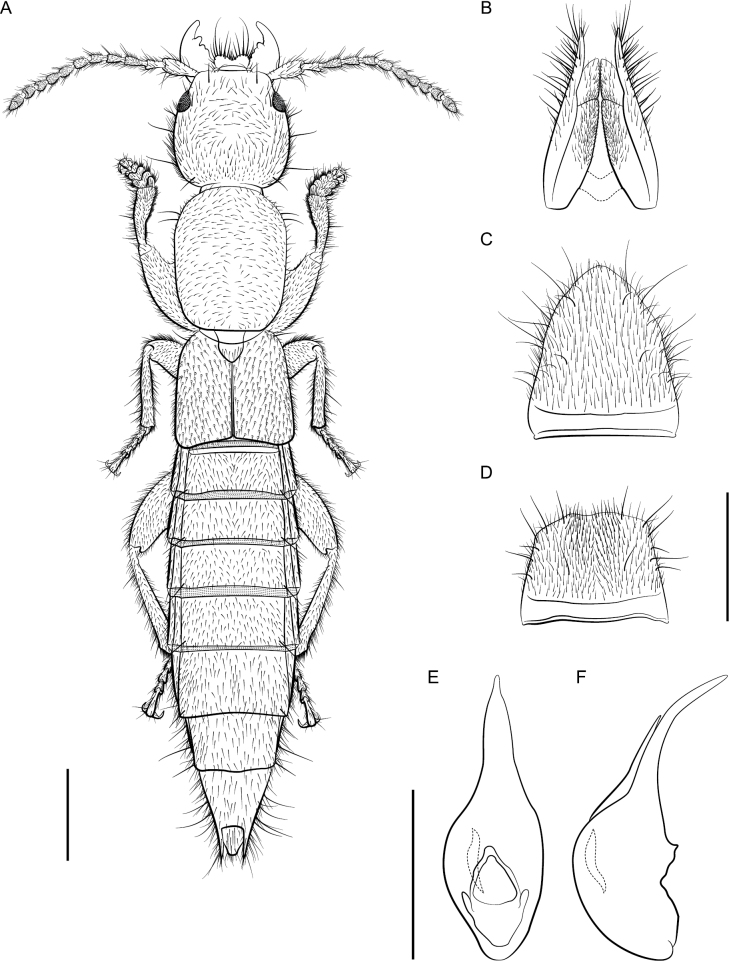
*Lathrobiumcamplyacra***A** habitus **B** female terminalia **C** female sternite VIII **D** male sternite VIII **E** aedeagus in ventral view **F** aedeagus in lateral view. Scale bars: 1 mm.

**Figure 6.**
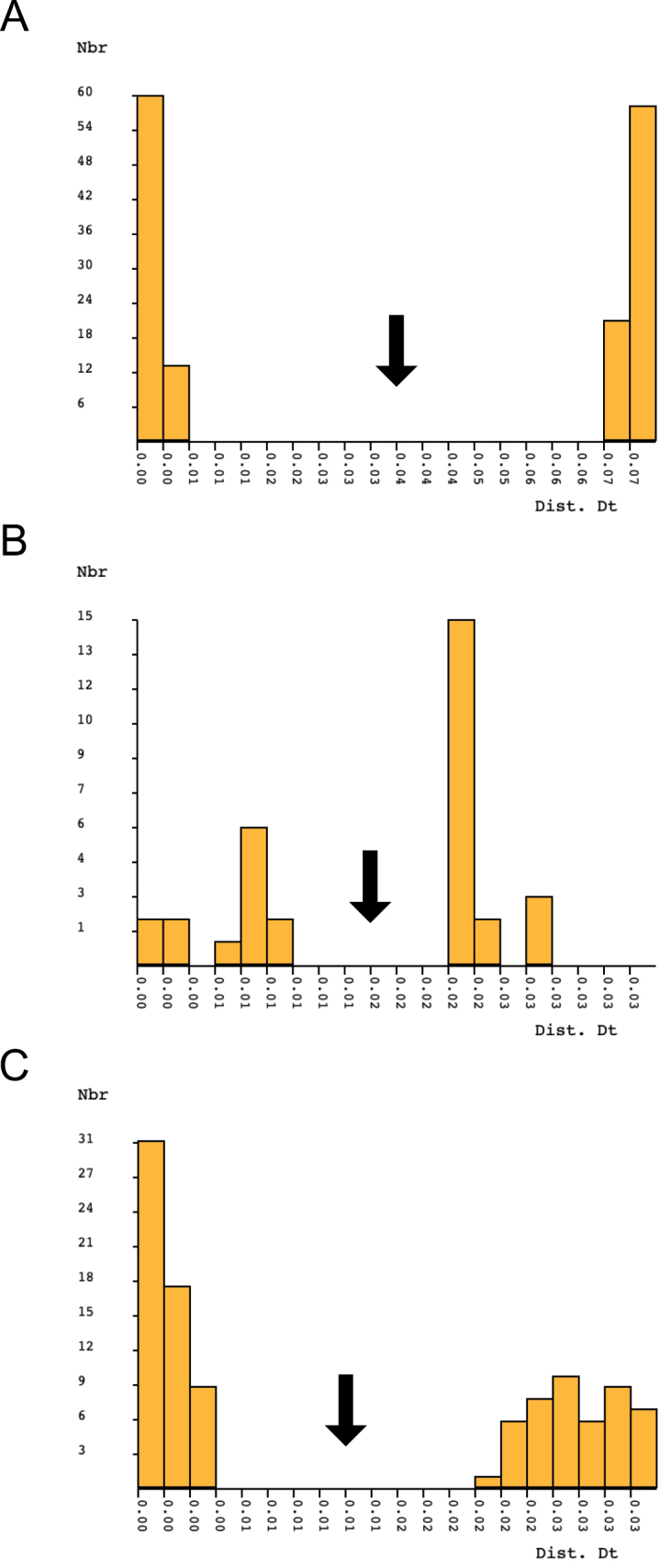
Species by Automatic Partitioning (ASAP) histograms of K2P distances for **A***Lathrobiumcarolinae* + *L.camplyacra***B***Lathrobiumislae* + *L.lividum*, and **C***Lathrobiumsmokiense* + *L.balsamense.* Y-axis is the number of sequence pairs (Nbr), and x-axis is K2P distance between paired sequences (Dist. Dt). Arrows indicate the “barcode gap” between intra- and interspecific variation.

The two morphotypes of *L.carolinae* resolved as reciprocally monophyletic with a deep divergence and 100% bootstrap support. The first clade (Fig. 3: *L.carolinae*) includes the more widespread morphotype which spans 250 km from Brasstown Bald, Georgia, to the French Broad River basin (Asheville depression), although most are west of the Little Tennessee River (with the disjunct exception of one individual form Wilkins Creek, north of Asheville). It is characterized by an aedeagus with a ventral process that is not projected ventrad and two structures on the internal sac visible when invaginated (Fig. 4D, E). The female terminalia are more robust and lack a subgenital plate (Fig. 4A). The second clade (Fig. 3: *L.camplyacra*) is restricted to the Plott Balsam, Great Balsam, and Cowee Mountains. Aedeagi in this morphotype have a ventral process that projects beyond the median foramen in lateral view (Fig. 5F) and one structure visible on the internal sac. The female terminalia are more elongate, with noticeably shorter coxites and a small, lightly sclerotized subgenital plate (Fig. 5B). Examination of 130 specimens found no intermediate forms and a single specimen of morphotype 2 out of range. Within-clade K2P distances were close to zero, and between clade distances were up to 7% (Fig. 6A). The highest-ranking ASAP scheme included three partitions, two corresponding to the two morphotypes and a third partition for a single low-quality read of morphotype 1. mPTP supported two putative species. The type locality of *L.carolinae* is on the border of the two ranges, but the aedeagus of the lectotype matches that of morphotype 1, so we designate morphotype 2 as a new species, *Lathrobiumcamplyacra* sp. nov., described below.

**Figure 7.**
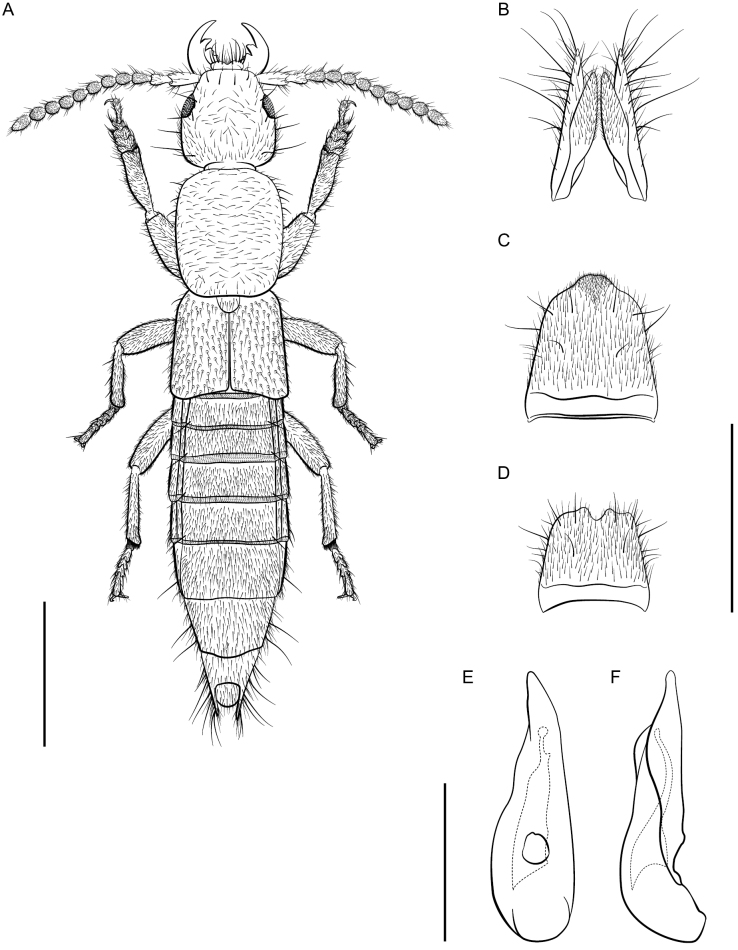
*Lathrobiumislae***A** habitus **B** female terminalia **C** female sternite VIII **D** male sternite VIII **E** aedeagus in ventral view **F** aedeagus in lateral view. Scale bars: 1 mm.

**Figure 8.**
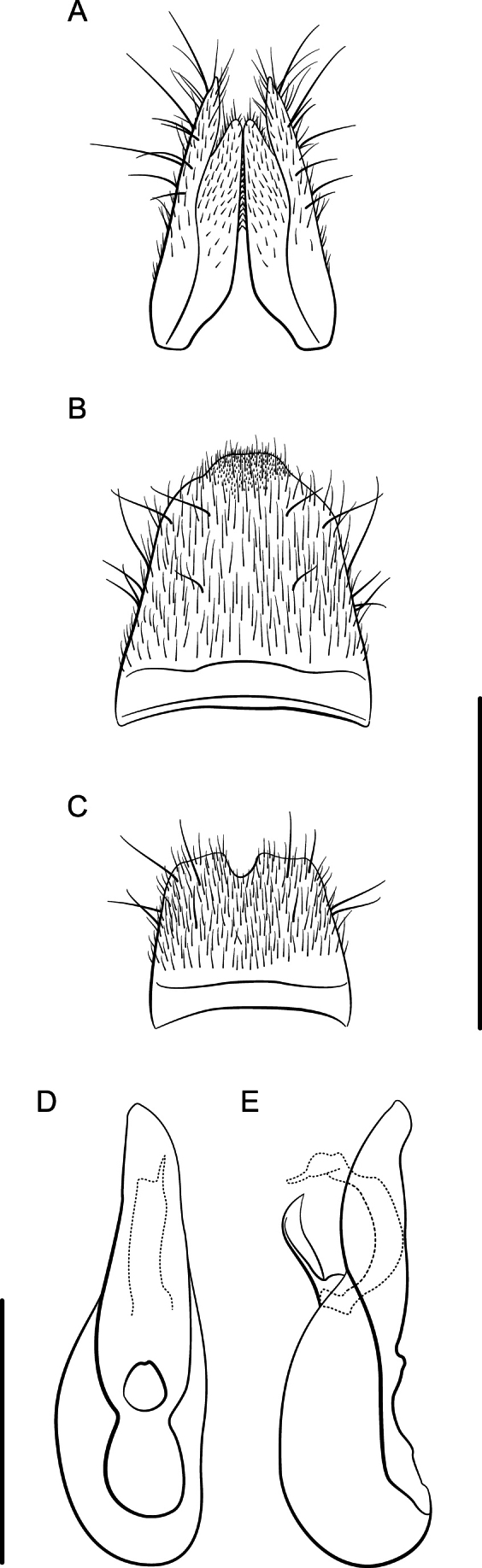
*Lathrobiumlividum***A** female terminalia **B** female sternite VIII **C** male sternite VIII **D** aedeagus in ventral view **E** aedeagus in lateral view. Scale bars: 1 mm.

The “*L.islae*” lineage occupies high-elevation (>1500 m) spruce-fir forests north of the French Broad River basin. A clade containing morphotype 1 (Fig. 3: *L.islae*) was paraphyletic with respect to morphotype 2 (Fig. 3: *L.lividum*). Intraspecific K2P distances ranged from 0 to 3% distance (Fig. 6B), and mPTP suggested the existence of a single species. The highest-ranking ASAP scheme included seven partitions, five containing only morphotype 1 and two containing only morphotype 2. Each morphotype has distinct genitalic morphology. The major spine of the internal sac of the aedeagus is long, sinuate, and club-tipped in morphotype 1 (Fig. 7E), but short, curved, and projecting above the median lobe in morphotype 2 (Fig. 8E). They also differ in the shape of the ventral process (straight vs apically projecting) and width of the gonocoxites. Examination of 54 specimens found no further morphological variation or intermediate forms. We designate morphotype 1 as *Lathrobiumislae* sp. nov. and morphotype 2 as *Lathrobiumlividum* sp. nov.

**Figure 9.**
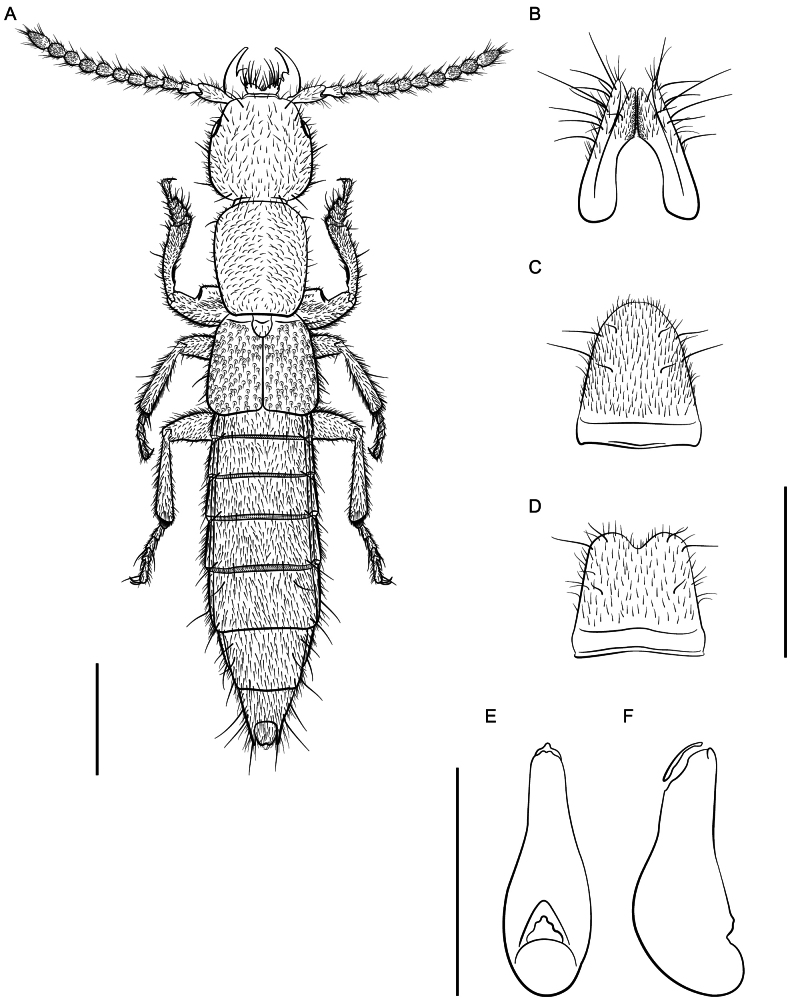
*Lathrobiumsmokiense***A** habitus **B** female terminalia **C** female sternite VIII **D** male sternite VIII **E** aedeagus in ventral view **F** aedeagus in lateral view. Scale bars: 1 mm.

**Figure 10.**
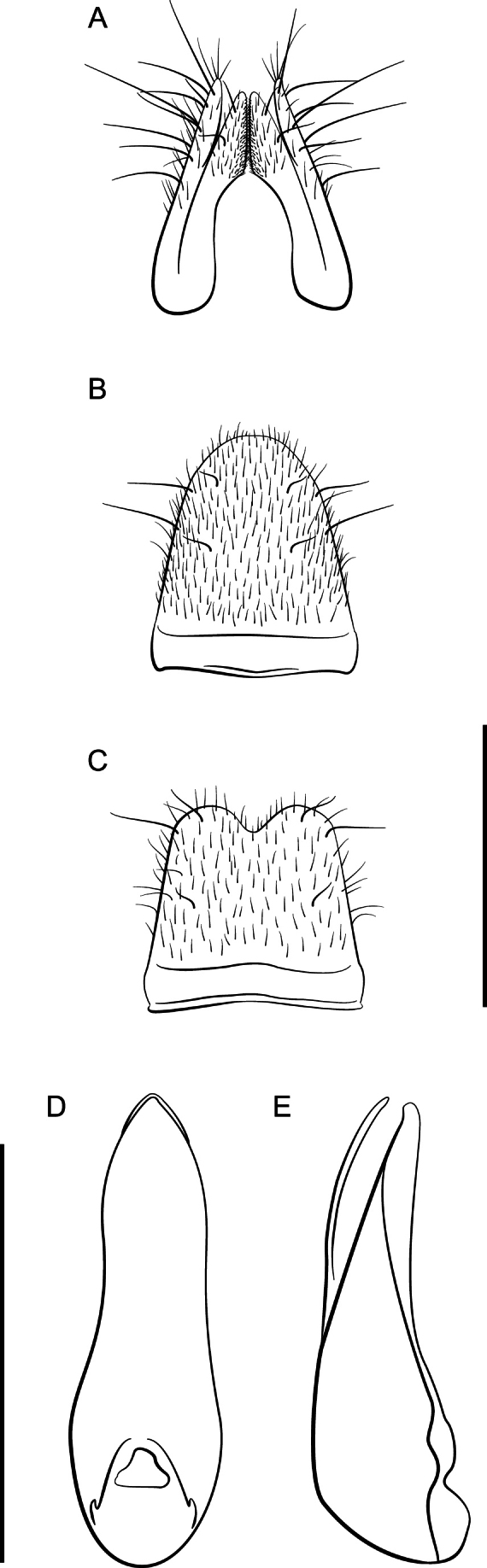
*Lathrobiumbalsamense***A** female terminalia **B** female sternite VIII **C** male sternite VIII **D** aedeagus in ventral view **E** aedeagus in lateral view. Scale bars: 1 mm.

The “*L.smokiense*” lineage inhabits high elevation spruce-fir forests south of the French Broad River basin. The morphotypes are geographically coherent, with morphotype 1 (Fig. 3: *L.smokiense*) restricted to the Great Smoky Mountains and morphotype 2 (Fig. 3: *L.balsamense*) to the Plott Balsam and Great Balsam Mountains. They can be distinguished only by their aedeagi. Morphotype 1 has a distinctive median lobe that is fully sclerotized and tube-like, with a small dorsal plate (operculum) positioned at the apex like a cap on the tube (Fig. 9E, F). The aedeagus of morphotype 2 is more typical of the genus, with a distinct ventral process and dorsal plate positioned dorsally (Fig. 10D, E). No distinguishing characters were found for females. Each morphotype was resolved as polyphyletic, intermingled in two clades (Fig. 3). The first clade contained a sequence of morphotype 1 from Clingmans Dome (cd) and three sequences of morphotype 2 from Richland Balsam (rb), two geographic extremes of the lineage’s range. The remaining sequences resolved in a mixed clade spread across the Smokies and Plott Balsams. Intraspecific K2P distances were low (Fig. 6C) and in several cases sequences from divergent morphotypes were identical. mPTP suggested one species, and the highest-ranking ASAP scheme included three partitions, one for the Clingmans Dome sequence (cd), one containing the three sequences from Richland Balsam (rb), and a mixed partition containing the remainder. Nonetheless, given the consistency of clear morphological differences in geographically disparate ranges, we designate morphotype 1 as *Lathrobiumsmokiense* sp. nov. and morphotype 2 as *Lathrobiumbalsamense* sp. nov. based primarily on morphology.

## ﻿Taxonomy

**Subfamily Paederinae Fleming, 1821**

**Tribe Lathrobiini Laporte, 1835**

**Subtribe Lathrobiina Laporte, 1835**

### ﻿Genus *Lathrobium* Gravenhorst, 1802

**Subgenus Abletobium Casey, 1905: 70.**

#### ﻿ Lathrobium (Abletobium) absconditum

Taxon classificationAnimaliaColeopteraStaphylinidae

Haberski & Caterino sp. nov.

293586F7-BB7B-5827-BB86-FFA82966BA0C

https://zoobank.org/F524D9C1-23EC-4511-9883-61137650F36E

##### Type material.

***Holotype*** ♂ (FMNH): “USA: VA: Bath Co., 38.0744°N, 79.6836°W, Dry Run Gorge, X.28.2018, C. Harden, oak-hickory woods; limestone gorge.” / “CLEMSON ENT [QR CODE] CUAC000185176”. ***Paratypes*** (3, CUAC, FMNH, VMNH): same data as holotype (CUAC000185174, CUAC000185175, CUAC000185177).

##### Other material.

Virginia: same data as holotype, CUAC000187891 (1 larva, CUAC); Highland Co.: Owl Cave, 2070’, Water Sinks (38.2205, -79.6046), C. Harden, 31 May–2 Aug 2019 (CUAC); Highland Co.: Water Sinks, 2080’ (38.2211, -79.6042), C. Harden, 4 May–31 May 2019 (VMNH).

##### Diagnosis.

This species is larger than most microphthalmous *Lathrobium* known from Virginia and West Virginia. Males can be distinguished from most species by the lack of setal combs on sternite VIII and females by the presence of gonocoxite lobes. *Lathrobiumsolum* is of similar size and also lacks the transverse combs on sternite VIII. However, *Lathrobiumsolum* has transverse antennomeres V–VII, and more widely separated gular sutures. Aedeagi differ in the shape of their major spines, and *L.solum* has a characteristically shaped ventral process that reaches the dorsal plate in lateral view. The female of *L.solum* is unknown, so no distinctions can be made.

##### Description.

**Figure 11.**
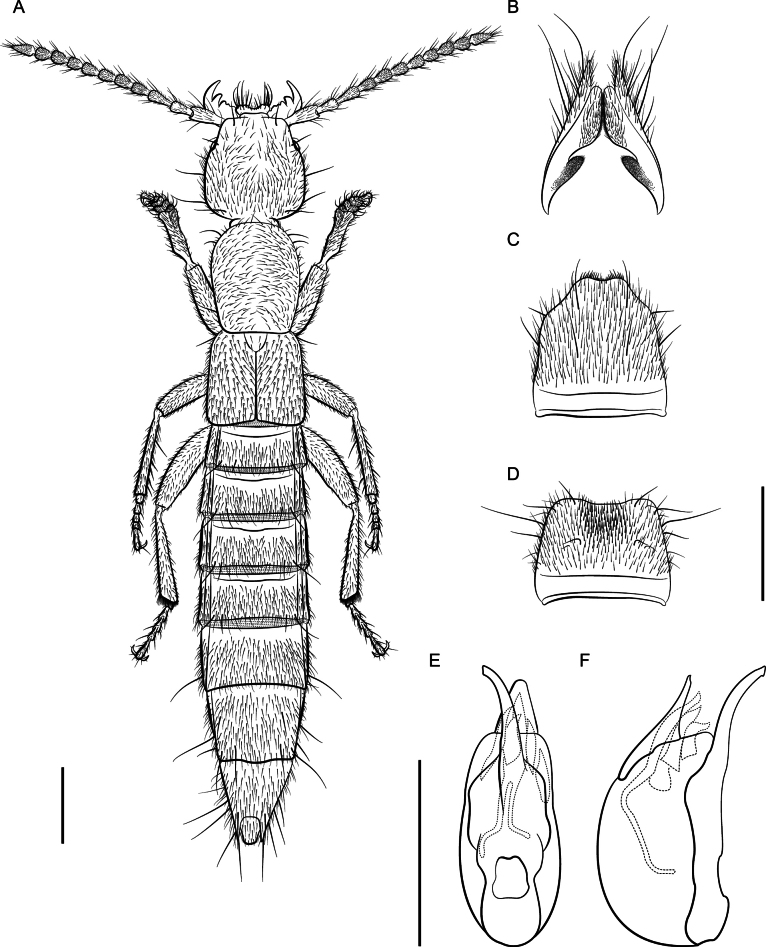
*Lathrobiumabsconditum***A** habitus **B** female terminalia **C** female sternite VIII **D** male sternite VIII **E** aedeagus in ventral view **F** aedeagus in lateral view. Scale bars: 1 mm.

Habitus (Fig. 11A). Large species, total body length ~ 9 mm long, FL 3.4–4.0 mm long. Coloration: body and appendages pale reddish.

Head slightly wider than long, widest at posterior; posterior angles rounded. Epicranium coarsely punctate with punctures less dense in median dorsal portion; interstices with strong transversely reticulate microsculpture throughout; head setose throughout, with long macrosetae projecting at posterior corners of head, corners of eyes, laterally posterior to eyes, and above mandible insertions; gular sutures (sulci) narrowing posteriorly until nearly touching; neck 1/2 as wide as head. Eyes reduced to small white membranes without ommatidia, occupying 1/9 length of head. Antennae moniliform, as long as head and pronotum combined; scape as long as antennomeres II and III combined; antennomeres II–IX obconic, longer than wide but become progressively wider; apical antennomere longer, subacute.

Pronotum longer than wide, narrower than head and elytra; widest at anterior angles and tapering slightly posteriorly; all angles round, posterior angles less so; punctation is dense, punctures spaced one diameter apart, impunctate at midline with a faint line visible on posterior two thirds; interstices shiny with no microsculpture. Elytra distinctly shorter than pronotum, as wide as head, approximately as long as wide; posterior margin sinuate; punctures small and shallow with indistinct edges, irregularly spaced, most one diameter apart; setae angled to posterior; interstices with finely punctate microsculpture. Hindwings vestigial. Posterior margin of abdominal tergite VII without palisade fringe.

♂: Larger, forebody 3.8–4.0 mm. Posterior margin of sternite VIII with broad but shallow emargination, patch of dense setae medially (Fig. 11D). Aedeagus 1.5 mm long; ventral process long, narrow, and asymmetrical (Fig. 11E, F); dorsal plate short and broad; internal sac with five spines, major spine long with two narrow asymmetrical processes.

**Figure 12.**
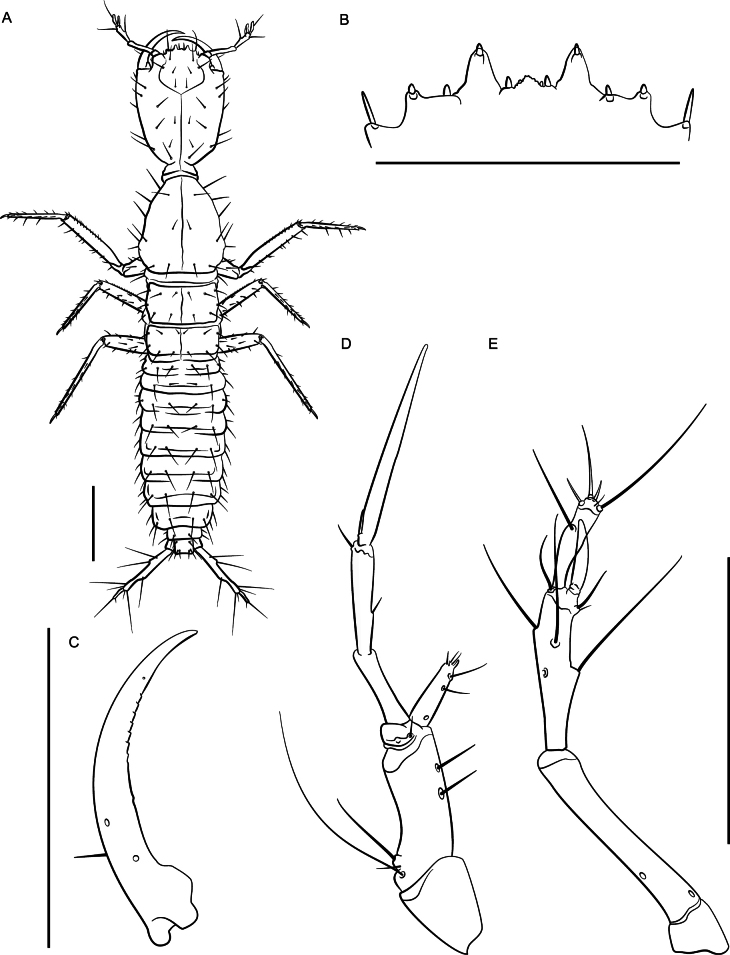
*Lathrobiumabsconditum* second instar larva **A** habitus **B** nasale **C** mandible **D** maxilla **E** antenna. Scale bars: 1 mm (**A, C**); 500 μm (**B, D, E**).

♀: Smaller, forebody 3.4 mm. Sternite VIII oblong, apex notched with micropubescence (Fig. 11C); paraprocts undivided anteriorly, apices longer than basal portion; proctiger conical; sternum IX with coxites and valvifers fused (Fig. 11B), base produced into distinct setose lobes; subgenital plate absent.

***Second instar larva***: Body elongate, ~ 7 mm long; lightly sclerotized (Fig. 12A), head more sclerotized; head and thoracic tergites light brown, legs and body white, translucent.

Head ovate, tapered posteriad (in dorsal view), dorsoventrally flattened, 1.3× as long as wide, with dorsal setae as in Fig. 12A; head 3.6× wider than neck; dorsal ecdysial lines bifurcate 2/5 distance between neck and nasale margin; stemmata absent; anterior margin of nasale (Fig. 12B) as in *L.hardeni*, but median tooth triangular with edge serrated; Apotome of gula not reaching tentorial pits.

Antennae (Fig. 12E) length ratios: 1.0:3.8:2.9:1.7; antennomere I triangular; antennomere II with two pores; antennomere III with three elongate macrosetae, three solenidia, one pore; antennomere IV club-shaped with apical solenidia; sensory appendage 0.8× as long as antennomere IV.

Mandibles (Fig. 12C) long, falciform, serrate along apical 1/3 of inner margin, with a single seta near base on outer ventral edge. Maxilla (Fig. 12D) with cardo triangular; stipes elongate, 1.7× longer than cardo; mala digitiform, tapering toward apex, 0.9× as long as palpomere I, with apical sensory appendages and two pores; palpifer with one seta. Maxilla and labium as in *L.hardeni*, except maxillary palpomere length ratios: 1.0:1.4:2.7; ligula separated from prementum by a distinctly sclerotized transverse strip; palpomere I 1.2× as long as II; palpomere II bent near apex.

Dorsal sclerites of thorax with ecdysial lines along midline; prothorax narrow, 1.1× as long as wide, narrowed anteriorly, chaetotaxy as in Fig. 12A; thoracic tergite II longer than tergite III; abdominal sclerites lightly sclerotized, with two small pleural sclerites per segment on each side; basal segment of urogomphus 4× as long as terminal segment, with seven prominent lateral setae; terminal segment of urogomphus slender, with one short and one long apical seta.

##### Etymology.

The specific name is Latin (singular, neuter), meaning hidden or concealed, in reference to the species’ endogean habitat.

##### Distribution and ecology.

**Figure 13.**
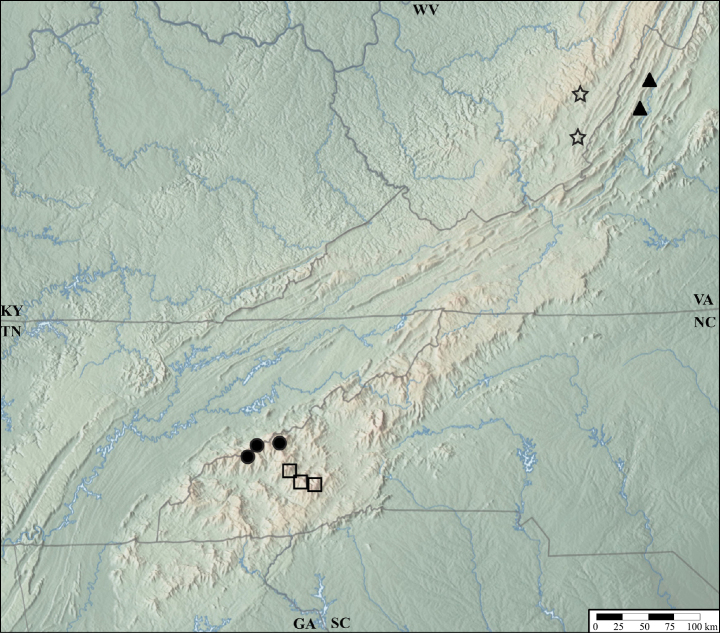
Distribution of *Lathrobiumabsconditum* (triangle), L. *balsamense* (square), *L.hardeni* (star), *L.smokiense* (circle).

*Lathrobiumabsconditum* is known from Highlands County and Bath County, Virginia (Fig. 13). Two specimens were collected near the mouth of Owl Cave, but none were found inside the cave. All specimens were collected with buried pipe traps, which suggests they are hypogean. Adults collected Mar–Oct. Larvae collected in Oct. Males and females have yet to be collected together.

##### Remarks.

Larvae were associated with adults by DNA barcoding.

#### ﻿ Lathrobium (Abletobium) balsamense

Taxon classificationAnimaliaColeopteraStaphylinidae

Haberski & Caterino sp. nov.

0E9F4960-06BE-5363-A92A-654F26953332

https://zoobank.org/5694CA73-B2C9-48EE-849E-2BD354476BA9

##### Type material.

***Holotype*** ♂ (FMNH): “USA: NC: Haywood Co., 35.3632°N, 82.9885°W, Richland Balsam Mountain, 6180’, IX.11.2019, Sifted Litter, M. Caterino.” / Caterino DNA voucher, Ext. MSC-4413, Morphosp RB.B.320” / “CLEMSON ENT [QR CODE] CUAC000003949”. ***Paratypes*** (4, CUAC, FMNH): 2: same locality as type, 35.3627°N, 82.9885°W, IX.11.2019 (CUAC000003627, CUAC000177150); same locality as type, 35.3630°N, 82.9890°W, 6200ft, v.08.2018 (CUAC000177151); same locality as type, 35.3676, -82.9902, 6398’, v.8.2018 (CUAC000048512).

##### Other material.

North Carolina: Jackson Co.: Balsam Mountain Preserve, Nantahala National Forest, (35.3751, -83.0981), S. Myers, 15 Jun 2015 (CUAC); Haywood Co.: Mt. Lyn Lowry, 6205’ (35.4640, -83.1100), M.S. Caterino, 22 Sep 2020 (2, CUAC); Haywood Co.: Mt. Lyn Lowry, 6192–6203’ (35.4640, -83.1101), M.S. Caterino, 15 Apr 2021 (9, CUAC); Haywood Co.: Mt. Lyn Lowry, 6200’ (35.4638, -83.1108), M.S. Caterino, 22 Apr 2020 (3, CUAC); Haywood Co.: Richland Balsam, 6398’, Blue Ridge Parkway (35.3676, -82.9902), M.S. Caterino, 8 May 2018 (USNM); Haywood Co.: Richland Balsam, 6069–6397’, Blue Ridge Parkway, A. Smetana, 25 May 1986 (CNC); Haywood Co.: Waterrock Knob, 6275’, Blue Ridge Parkway (35.4643, -83.1374), M.S. Caterino, 29 May 2018 (4, CUAC); Haywood Co.: Waterrock Overlook, 5800’, Blue Ridge Parkway, J.M. & B.A. Campbell, 1 Sep 1967 (4, CNC); Haywood Co.: Browning Knob, 6003–6200’, Blue Ridge Parkway, A. Smetana, 28 May 1986 (CNC); Haywood Co.: Browning Knob, 6140’, Blue Ridge Parkway (35.4630, -83.1310), 22 Sep 2020, M.S. Caterino (7, CUAC); Haywood Co.: Browning Knob, 6200–6220’, Blue Ridge Parkway (35.4633, -83.1315), 22 Sep 2020 M.S. Caterino, A. Haberski (5, CUAC).

##### Diagnosis.

This species can be distinguished from its close relative, *L.smokiense*, only by their aedeagi. In *L.smokiense*, the entire aedeagus is well sclerotized, whereas the aedeagus of *L.balsamense* is more typical for the genus with a distinct ventral process (Fig. 9E, F vs Fig. 10D, E). Females can only be identified by geography, association with males, or DNA.

One other microphthalmous species, *L.shermani* Fall, 1917, occurs above 1000 m elevation in the southern Appalachians, although their ranges do not overlap. Adults are similar in size and appearance but are distinguished by the sexual characters. Males of *L.balsamense* lack a conspicuous comb of thick black setae on sternite VIII and has no spines on the internal sac of the aedeagus. In females, the gonocoxites lack the pubescence of *L.shermani* in the lower half.

##### Description.

Habitus (Fig. 9A). Small species, total body length ~ 6 mm long, FL 2.6–2.8 mm long. Coloration: body reddish becoming lighter towards posterior segments of abdomen; legs, palpomeres, and antennae a paler reddish yellow.

Head subquadrate, posterior angles rounded; epicranium coarsely punctate with punctures less dense in median dorsal and anterior regions; interstices with strong transversely reticulate microsculpture throughout; head setose throughout, with long macrosetae projecting at posterior corners of head, corners of eyes, laterally posterior to eyes, and above mandible insertions; gular sutures straight and widely separated, 1/8 width of head but narrowing slightly posteriorly; neck 1/2 as wide as head. Eyes reduced to small white membranes without ommatidia, occupying 1/9 length of head. Antennae moniliform, as long as head and pronotum combined; scape as long as antennomeres II and III combined; antennomeres II–IV obconic and elongate, gradually widening so that antennomeres V–IX are as wide as long; apical antennomere longer, subacute.

Pronotum longer than wide, narrower than head and elytra; widest at anterior angles and tapering slightly posteriorly; anterior angles round, posterior angles less so; punctures small, spaced 1–2× their diameter apart, impunctate at midline; interstices shiny with no microsculpture. Elytra shorter than pronotum, as wide as head, approximately as long as wide; posterior margin slightly sinuate; punctures large and shallow with indistinct edges, irregularly spaced, most approximately one diameter apart; setae angled to posterior; interstices with finely punctate microsculpture. Hindwings vestigial, 0.2 mm long, 1/4 length of elytra. Posterior margin of abdominal tergite VII without palisade fringe.

♂: Posterior margin of sternite VIII with a shallow U-shaped notch (Fig. 10C). Aedeagus 1.1 mm long (Fig. 10E, F), ventral process long, broad, and symmetrical, tapering to a blunt tip; dorsal plate long and situated dorsally; internal sac without spines.

♀: Sternite VIII conical (Fig. 9B); paraprocts undivided anteriorly, apices as long as basal portion; proctiger conical; sternum IX with coxites and valvifers fused, basal half glabrous (Fig. 9B); subgenital plate absent.

##### Etymology.

This species is named for the Plott Balsam and Great Balsam Mountains.

##### Distribution and ecology.

*Lathrobiumbalsamense* inhabits spruce-fir forests above 1500 m in the Great Balsam Mountains and Plott Balsam Mountains (Fig. 13) but is absent from the Great Smoky Mountains where the closely related *L.smokiense* occurs. It does not occur in spruce-fir forests north of the French Broad River basin, where its microhabitat is inhabited by *L.lividum* and *L.islae*. It can be collected from leaf litter but is most common beneath bryophyte mats on boulders. Collected May–Sep.

#### ﻿ Lathrobium (Abletobium) hardeni

Taxon classificationAnimaliaColeopteraStaphylinidae

Haberski & Caterino sp. nov.

0316C7FF-374B-54D5-9DDC-B1E69CC3D9BF

https://zoobank.org/07EEDC41-8050-4B49-B07A-539459101D12

##### Type material.

***Holotype*** ♂ (FMNH): “USA: West Virginia, Pocahontas Co., Monongahela Nat. For. Cranberry Backcountry, past glades, 38.2101, -80.2871, 11-June-2019, C.W. Harden, under large rock nr. steep stream. Spruce/ Northern hardwoods.” / “Caterino DNA voucher, Ext. MSC-7071” / “CLEMSON ENT [QR CODE] CUAC000169036”. ***Paratypes*** (4, FMNH, CUAC, VMNH): 3: “USA: WEST VIRGINIA, Pocahontas Co. Monongahela Nat. For. Kennison Mtn Tr., W of Cranberry Glades, 1224m, 38.18002, -80.27846, 10-June-2019, C.W. Harden, Under rock during rain” (also 2 larvae from this collecting event). 1: “USA: WEST VIRGINIA, Pocahontas Co. Monongahela Nat. For. Kennison Mtn Tr., 38.19114N, 80.28524W, 1181m elev. 11.June–3.August.2019, C.W. Harden & L.M. Thompson, Buried jar trap, northern hardwood forest, sandstone boulders present. Rich dark rocky soil. KEN-04” (also 2 larvae from this collecting event).

##### Other material.

West Virginia: Pocahontas Co.: Dogway Rd., 4019’, Cranberry Wildlife Management Area (38.1903, -80.2893), C. Harden, 02 Sep 2018 (5, CUAC, VMNH); Pocahontas Co.: Monongahela Nat. For. Cranberry Backcountry (38.1800, -80.2785), C. Harden, 10 Jun 2019, CUAC000187895, CUAC000187896 (3 larvae, CUAC); Pocahontas Co.: Kennison Mountain Trail, Cranberry Wildlife Management Area (38.1900, -80.2780), C. Harden, 11 Sep 2017 (CUAC); Pocahontas Co.: Kennison Mountain Trail, 4388’, Kennison Mountain (38.1979, -80.2915), C. Harden, 19 Sep 2018 (CUAC). Pocahontas Co.: Kennison Mountain Trail, W. of Cranberry Glades, 4015’, Kennison Mountain (38.18002, -80.27846), C. Harden, 06 Oct 2019 (CUAC); Pocahontas Co.: Monongahela Nat. For. Kennison Mtn Tr. (38.19114, -80.28524), C. Harden, 11 Jun–3 Aug 2019, CUAC000187894 (1 larva, CUAC); Pocahontas Co.: Monongahela Nat. For. Kennison Mtn Tr. (38.1903, -80.2893), C. Harden, 2 Sep 2018, CUAC000187897, CUAC000187898 (5 larvae, CUAC).

##### Diagnosis.

Three other species of microphthalmous *Lathrobium* are known from West Virginia and Virginia, *L.absconditum*, *L.lapidum*, and *L.shermani*. The extents of their ranges are unknown and might overlap. Adults of *L.hardeni* can be distinguished from those of *L.absconditum* by its smaller body size, transverse combs of thick setae on male sternite VIII, and the presence of a ventral process in the female genitalia, which is unique among the microphthalmous *Lathrobium* of North America. Males of *L.shermani* are approximately the same size but have a single row of thick black setae on sternite VIII. Males of *L.lapidum* are most similar in appearance but are larger and have quadrate elytral margins. The aedeagus of *L.lapidum* has a characteristic ventral process that reaches the dorsal plate in lateral view, and asymmetrical spines on the internal sac.

##### Description.

**Figure 14.**
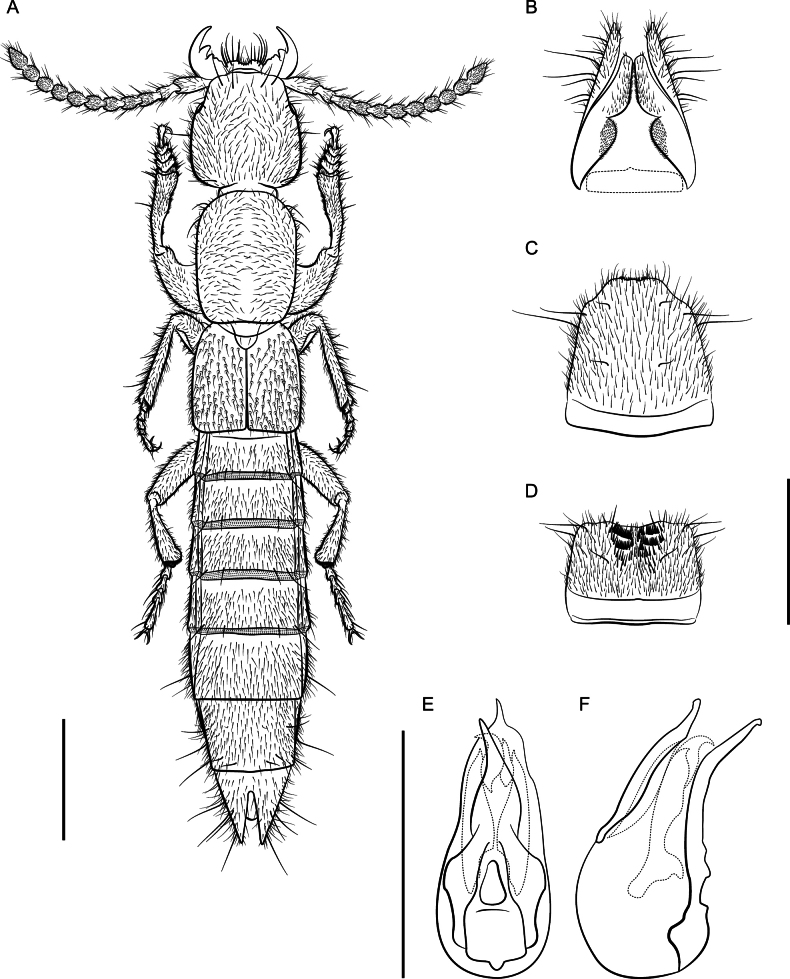
*Lathrobiumhardeni***A** habitus **B** female terminalia **C** female sternite VIII **D** male sternite VIII **E** aedeagus in ventral view **F** aedeagus in lateral view. Scale bars: 1 mm.

Habitus (Fig. 14A). Small species, total body length ~ 6 mm long, FL 3.1–3.3 mm long. Coloration: body pale reddish becoming lighter towards posterior segments of abdomen; legs, palpomeres, and antennae paler reddish yellow.

Similar in appearance to *L.absconditum*, except head subquadrate; gular sutures straight, separated by 1/15 width of head and narrowing slightly posteriorly. Eyes reduced to small white membranes without ommatidia, occupying 1/8 lateral width of head. Pronotum with sides parallel, just perceptibly narrowed posteriorly; punctation spaced less than one diameter apart, impunctate at midline. Posterior margins of elytra less sinuate; punctures larger.

♂: Posterior margin of sternite VIII with broad but shallow emargination and three transverse combs of thick black setae to either side of midline (Fig. 14D). Aedeagus 1.2 mm long; ventral process long, curved ventrally, narrowing to a rounded point with small apical tooth (Fig. 14E, F); dorsal plate long and broad, distal end narrowing to an abrupt point curved away from ventral process with small apical tooth; internal sac with four more-or-less symmetrical spines.

♀: Sternite VIII oblong, apex notched, with micropubescence (Fig. 14C); paraprocts undivided anteriorly, apices shorter than basal portion; proctiger conical; sternum IX with valvifers and coxites fused, finely setose (Fig. 14B); subgenital plate square, distal end with round projection.

**Figure 15.**
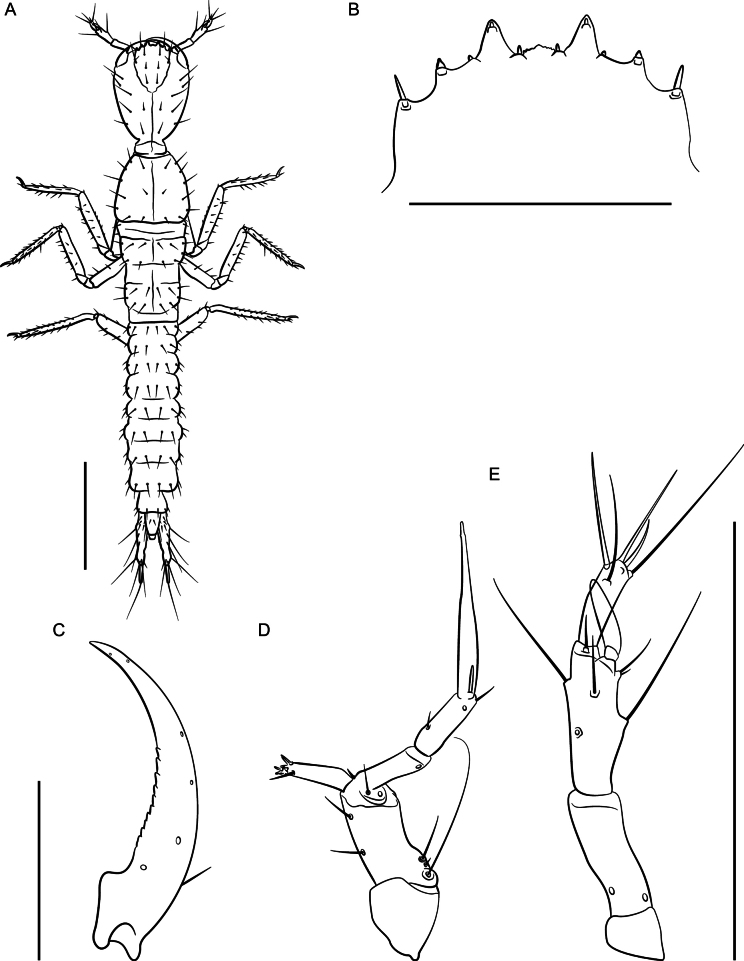
*Lathrobiumhardeni* first instar larva **A** habitus **B** nasale **C** mandible **D** maxilla **E** antenna. Scale bars: 1 mm (**A)**; 250 μm (**B, C**); 500 μm (**D, E**).

***First instar larva***: Body elongate, 4 mm long; lightly sclerotized (Fig. 15A), head more sclerotized; head light brown, legs and body white, translucent.

Head ovate, tapered posteriad (in dorsal view), dorsoventrally flattened, 1.2× as long as wide, dorsal setae as in Fig. 15A; head 3.2× longer than neck; dorsal ecdysial lines bifurcate halfway between neck and nasale margin; stemmata absent; anterior margin of nasale (Fig. 15B) with nine blunt teeth pointing anteriorly, one short median tooth with edge emarginated, a pair of paramedian teeth, and three pairs of lateral teeth; innermost lateral teeth are small and indistinct; paramedian and lateral teeth armed with nodular setae, and a pair of nodular setae separate median and paramedian teeth; Apotome of gula just reaching tentorial pits.

Antennae (Fig. 15E) length ratios: 1.0:2.4:2.7:1.6; antennomere I triangular; antennomere II with two pores; antennomere three with three elongate macrosetae, three solenidia, one pore; antennomere IV parallel sided with apical solenidia; sensory appendage 0.8× as long as antennomere IV.

Mandibles (Fig. 15C) long, falciform, serrate along basal 1/3 of inner margin, with a single seta near base on outer ventral edge. Maxilla (Fig. 15D) with cardo triangular; stipes elongate, 1.3× longer than cardo; mala digitiform, tapering toward apex, 0.9× as long as palpomere I, with apical sensory appendages and two pores; palpifer with one seta. Maxillary palpomere length ratios: 1.0:1.1:2.5; palpomere II with two setae; palpomere III with one basal sensory appendage and numerous small apical appendages. Labium with prementum subquadrate, basal portion strongly sclerotized; ligula with elongate membranous apex, 3× as long as wide, densely fimbriate, separated from prementum by a lightly sclerotized transverse strip; palpomere I 1.3× as long as II; palpomere II bearing short sensilla at apex.

Dorsal sclerites of thorax with ecdysial lines along midline of body; prothorax as long as wide, as long as tergite II and III combined, narrowed anteriorly, with chaetotaxy as in Fig. 15A; thoracic tergite II wider than III, but with similar chaetotaxy; abdominal sclerites lightly sclerotized, with two small pleural sclerites per segment on each side; basal segment of urogomphus 3× as long as terminal segment, with seven prominent lateral setae; terminal segment of urogomphus slender, with one short and one long apical setae.

**Figure 16.**
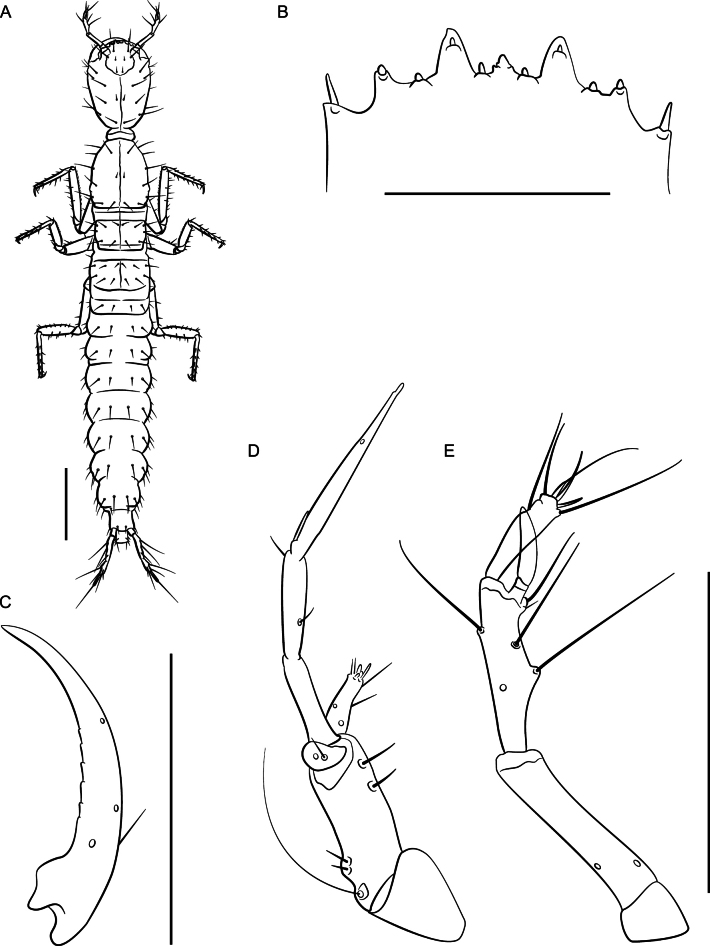
*Lathrobiumhardeni* second instar larva **A** habitus **B** nasale **C** mandible **D** maxilla **E** antenna. Scale bars: 1 mm (**A**); 250 μm (**B**); 500 μm (**C, D, E**).

***Second instar larva***: Second instar (Fig. 16A) resembles first, except as follows. Body larger, ~ 7 mm long. Head 1.4× as long as wide, dorsal ecdysial lines bifurcate 1/3 distance between nasale margin and neck; median tooth of nasale projecting, trifurcate (Fig. 16B). Antenna (Fig. 16D) length ratios: 1.0:2.4:2.7:1.6; antennomere IV club shaped. Maxillary palpomere length ratios: 1.0:1.2:2.8 (Fig. 16C). Labial palpomere I 1.6× longer than II and distinctly curved; palpomere II bent near apex. Thoracic tergite II narrower than III; urogomphi slender.

##### Etymology.

Named in honor of the collector Curt Harden. Curt designed the buried pipe trap that has been instrumental in collecting microphthalmous *Lathrobium* and was the first to collect several of the species described here.

##### Distribution and ecology.

*Lathrobiumhardeni* is known from two locations in the Monongahela National Forest, West Virginia (Fig. 13). Specimens have been collected in buried pipe traps and from underneath embedded rocks, which suggest this species is hypogean. Adults collected May–Sep. Larvae collected Aug–Sep in the same trap as adults.

#### ﻿ Lathrobium (Abletobium) lapidum

Taxon classificationAnimaliaColeopteraStaphylinidae

Haberski & Caterino sp. nov.

6E29A850-B119-51C3-BD9A-E02985716EEC

https://zoobank.org/351364B1-0580-44E7-B8C7-AF4F09A92E52

##### Type material.

***Holotype*** ♂ (FMNH): “USA: VA: Dickenson Co., 37.2724°N, 82.2956°W, Breaks Interstate Park, Camp Branch Trail, VI.09.2022, C. Harden, under large rock in sandy soil nr rock face, mesic stream hollow.” / “Caterino DNA voucher, Ext. MSC-11164”/ “CLEMSON ENT [QR CODE] CUAC000169038”. ***Paratypes* (1, VMNH)**: “USA: VA: Scott Co., 36.8584°N, 82.4477°W, Jefferson NF, N of Dungannon, VI.09.2022, C. Harden, On rock face under root/soil mat on base of boulder.” / “Caterino DNA voucher, Ext. MSC-11165” / “CLEMSON ENT [QR CODE] CUAC000169039”.

**Figure 17.**
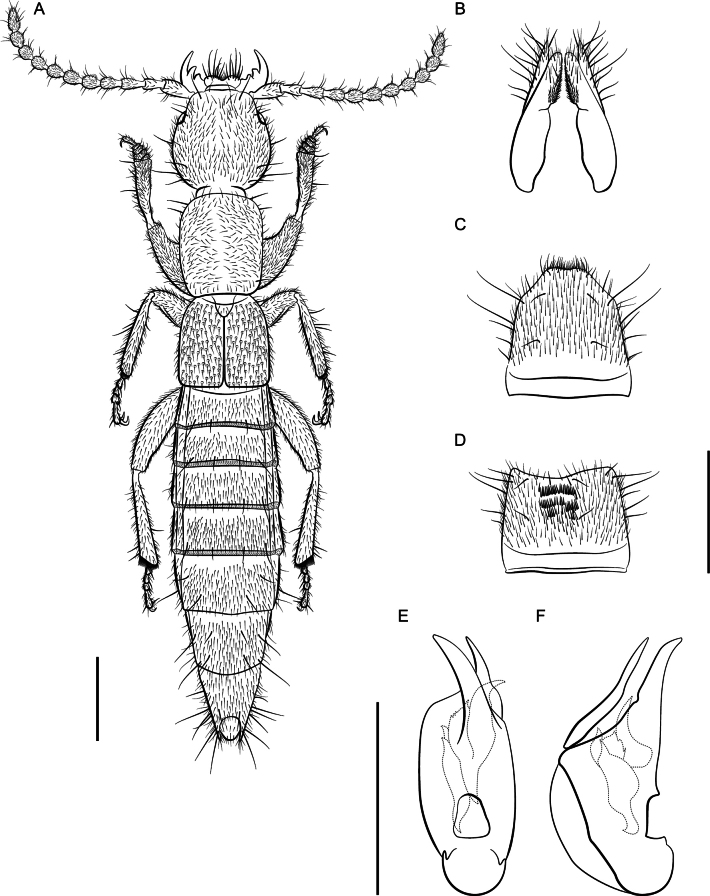
*Lathrobiumlapidum***A** habitus **B** female terminalia **C** female sternite VIII **D** male sternite VIII **E** aedeagus in ventral view **F** aedeagus in lateral view. Scale bars: 1 mm.

##### Diagnosis.

This species’ range might overlap with *L.absconditum*, *L.shermani*, and *L.hardeni*. Compared to these, it has a more rounded head, square elytral margins, and glabrous bases of the gonocoxites. It can be further distinguished from *L.absconditum* by its smaller size and transverse black setae on male sternite VIII. Males of *L.shermani* are approximately the same size but have a single setal comb on sternite VIII. *Lathrobiumhardeni* is most similar and distinguishing the two requires comparison of genitalia. Males of *L.hardeni* have more-or-less symmetrical spines on the internal sac of the aedeagus, and females have a subgenital plate.

##### Description.

Habitus (Fig. 17A). Large species, total body length ~ 8 mm long, FL 3.5 mm long. Coloration: body and appendages pale reddish.

Similar to *L.hardeni*, except posterior angles of head more strongly rounded. Eyes smaller, reduced to small white membranes occupying 1/9 lateral width of head.

Pronotum with punctures spaced one diameter apart, no line visible down midline. Posterior margin of elytra quadrate; punctures small and shallow, relatively sparse, irregularly spaced 2× their diameters apart.

♂: Posterior margin of sternite VIII with a broad, shallow, V-shaped emargination and three transverse combs of irregularly spaced thick black setae either side of midline (Fig. 17D). Aedeagus 1.3 mm long; ventral process long, reaching dorsal plate in lateral view, apex curved, asymmetrical (Fig. 17E, F); dorsal plate short and oval, distal end with a curved spine; internal sac with four spines, one bulbous and covered with short spikes.

♀: Sternite VIII oblong, apex notched with micropubescence (Fig. 17C); paraprocts undivided anteriorly, apices longer than basal portion; proctiger conical; sternum IX with valvifers and coxites fused, setose in apical 2/3 with a dense patch of thick setae at base (Fig. 17B); subgenital plate absent.

##### Etymology.

The specific name is from Latin, meaning stone, because both specimens were found on or under rocks.

##### Distribution and ecology.

**Figure 18.**
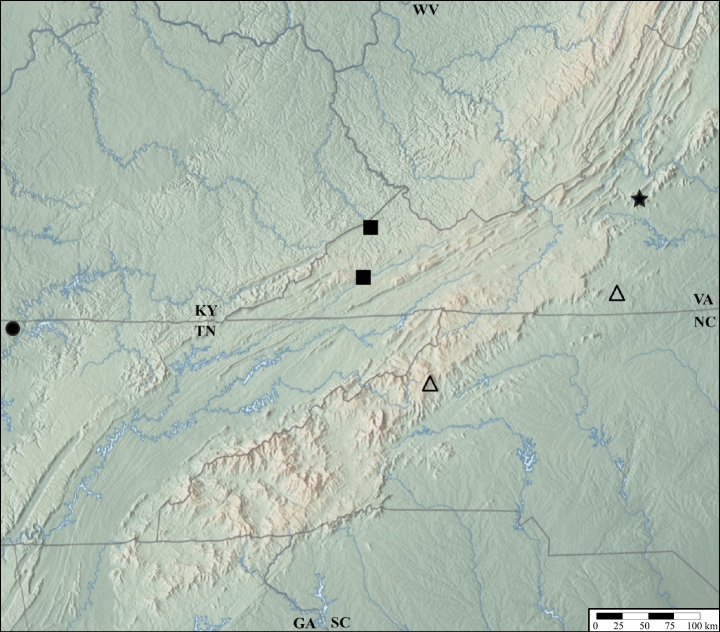
Distribution of *Lathrobiumlapidum* (star), *L.shermani* (triangle), *L.solum* (square), *L.thompsonorum* (circle)

*Lathrobiumlapidum* is known from two specimens collected in Jefferson National Forest, Virginia (Fig. 18). One was collected under an embedded rock, and the other beneath a root and soil mat on top of a boulder. Collected in June.

#### ﻿ Lathrobium (Abletobium) pallescens

Taxon classificationAnimaliaColeopteraStaphylinidae

(Casey, 1905)

9568180B-0FEE-5C1C-9E88-CEC8D48FB2FB

Abletobium pallescens Casey, 1905: 79.
Lathrobium (Abletobium) pallescens : Bernhauer and Schubert 1912: 265.

##### Type material.

***Lectotype***, *Abletobiumpallescens* Casey herein designated (USNM): “MASS / CASEY bequest 1925 / [red] TYPE USNM 38106 / [handwritten] *Abletobiumpallescens* Jul / Lectotype *Abletobiumpallescens* Casey Desg. Haberski & Caterino.”

##### Other material.

Canada, Ontario: Carleton Co.: Fitzroy Provincial Park, 02 May 1979, A. & Z. Smetana, (1 CNC); Grey Co.: Ingli’s Falls, 24 Jun 1985, B. Sinclair (1 CNC). Quebec: Haut Saint François: Johnville, 01 Nov 1987, C. Levesque (1 CNC); same locality, 03 Jul 1988 (1 CNC); same locality, 30 Oct 1988 (1 CNC).

##### Diagnosis.

This species can be distinguished from all other *Abletobium* by the presence of eyes with ommatidia.

##### Description.

**Figures 19, 20.**
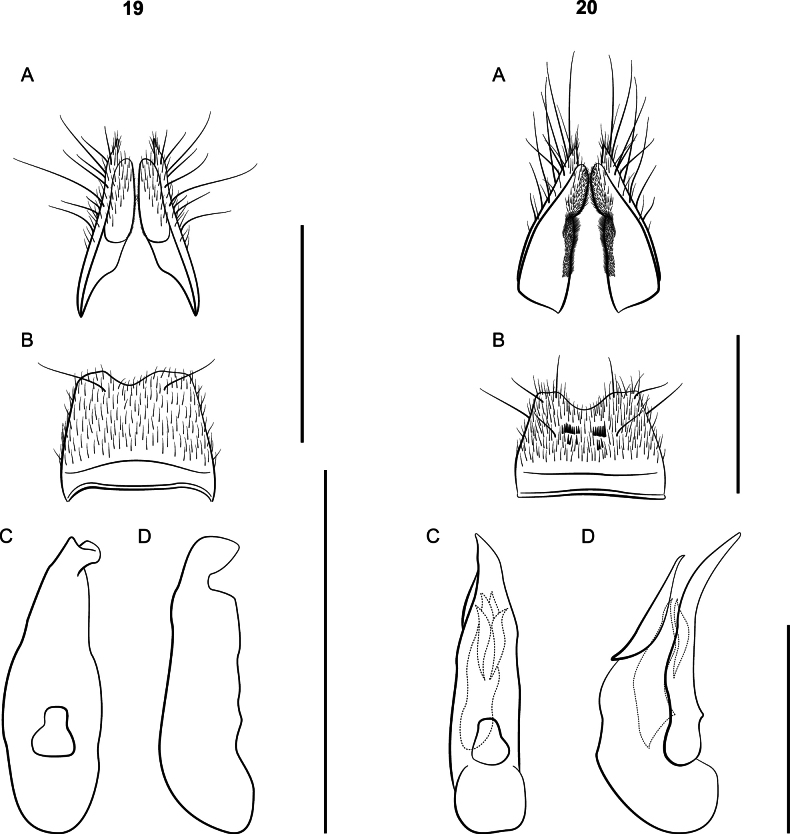
*Lathrobium* terminalia **A** female terminalia **B** male sternite VIII **C** aedeagus in ventral view **D** aedeagus in lateral view. **19***L.pallescens***20***L.shermani.* Scale bars: 1 mm.

Body length 6 mm; Body coloration pale red. Eyes small, ~ 30 ommatidia. Head wider than pronotum; gular sutures parallel, widely separate throughout; antennomeres V–VII 1.8× longer than wide. Elytra slightly shorter than pronotum. Females with paraprocts undivided, apices longer than basal portion; sternite VIII conical with small apical notch. Median lobe of aedeagus fully sclerotized and tube-like (Fig. 19).

##### Distribution.

Canada: **ON**, **QC**. USA: MA.

##### Remarks.

This species had not been previously recorded in Canada because CNC specimens had been misidentified as *L.shermani*. We corrected these identifications.

#### ﻿ Lathrobium (Abletobium) shermani

Taxon classificationAnimaliaColeopteraStaphylinidae

Fall, 1917

E8EE47D2-3143-55E9-9FC1-5F00D57EA74E

Lathrobium shermani Fall, 1917: 164.

##### Type material.

**Holotype** ♂ (MCZ): “[Handwritten] Grandfather Mt Early Sep 1915 N.C. 4000 to 5000 ft. / ♂ / FSherman Collector / H. C. FALL COLLECTION / TYPE [handwritten] *shermani* / [red] M. C. Z. Type 24086.”

##### Other material.

USA: Virginia: Patrick Co.: Stuart (36.7004, -80.2622), 12 Aug–10 Nov 2022, K. Ivanov, J. Means, L. Hightower (5 CUAC and VMNH).

##### Diagnosis.

*Lathrobiumshermani* is similar in appearance to *L.hardeni*, *L.lapidum*, and *L.thompsonorum*, all of which have combs of black setae on male sternite VIII. However, those species all have multiple combs, and the combs are continuous across the midline. They also differ in the shape and spines of the aedeagus. Females of *L.shermani* can be distinguished from *L.hardeni* by the lack of a subgenital plate, and from *L.thompsonorum* and *L.lapidum* by the pubescent gonocoxites.

##### Description.

We present descriptions and illustrations of the male and female genitalia, which were missing from the original description.

Body length 6 mm; body coloration pale red. Head wider than pronotum; eyes reduced to small white membranes without ommatidia; gular sutures parallel and widely separate; antennomeres V–VII 1.8× longer than wide. Elytra shorter than pronotum.

♂: Type specimen differs from specimens collected in Virginia in the number of thick black setae on sternite VIII: type has ~ 8 per comb whereas Virginia specimens have five. Aedeagus large, 1.4 mm long; ventral process long, broad, and bent ventrally in lateral view (Fig. 20D, E); lightly sclerotized median lobe protrudes beyond ventral process in lateral view; dorsal plate short and diamond-shaped; internal sac with four spines, major spine wide and 1/2 as long as ventral process, other three spines shorter than dorsal plate.

♀: Sternite VIII with a shallow, curved notch at apex (Fig. 20B); paraprocts undivided anteriorly, apices approximately equal in length with basal portion; proctiger conical; sternum IX with valvifers and coxites fused, setose with fine pubescence on lower 2/3 (Fig. 20C); subgenital plate absent.

##### Distribution.

USA: NC, **VA**.

##### Remarks.

*Lathrobiumshermani* was previously known from only the holotype. Recently, additional specimens were collected in buried pipe traps in Stuart County, Virginia (Fig. 18). Fall (1917) did not state the method used to collect the holotype, but several attempts by the authors to recollect it at the type locality via litter sifting failed. In their checklist of Canadian beetles, Bousquet et al. (2013) listed *L.shermani* as occurring in Ontario, but this was due to a misidentification of *L.pallecens*. *Lathrobiumshermani* is restricted to the southeastern USA.

#### ﻿ Lathrobium (Abletobium) smokiense

Taxon classificationAnimaliaColeopteraStaphylinidae

Haberski & Caterino sp. nov.

B9D316E5-E691-5F31-8F9E-421EB03974F7

https://zoobank.org/63F86704-4FC0-4F62-85C9-94A7201D1F04

##### Type material.

***Holotype*** ♂ (FMNH): “USA: TN: Sevier Co., 35.6308°N, 83.3904°W, Mt. Kephart, V.6.2018, M. Caterino, Sifted Litter.” / Caterino DNA voucher, Extraction MSC-6218., Extraction Date: XXX/ “CLEMSON ENT [QR CODE] CUAC000169009”. ***Paratypes*** (21, FMNH, CUAC, GSNP): 2: same data as holotype (CUAC000169008, CUAC000169010); 8: same locality as type, 35.6311°N, 83.3895°W, VI.5.2018 (CUAC000079121, CUAC000169007, CUAC000169011); 6: same locality as type, 35.6311°N, 83.3895°W, IX.14.2021 (CUAC000156757, CUAC000177157, CUAC000177158, CUAC000177159, CUAC000177160, CUAC000177161); 5: same locality as type, 35.6308°N, 83.3904°W, 6190ft, vi.05.2018 (CUAC000177152, CUAC000177153, CUAC000177154, CUAC000177155, CUAC000177156).

##### Other material.

North Carolina: Haywood, Co.: Balsam Mountain Trail, 5167’, Great Smoky Mountains National Park (35.6425, -83.2007), M.S. Caterino, 6 May 2020 (2, CUAC, GSNP); Haywood Co.: Big Cataloochee Mountain, 5725’, Great Smoky Mountains National Park (35.6425, -83.2007), M.S. Caterino, C. Harden, 14 Jul 2020 (3: CUAC, GSNP); Haywood Co.: Big Cataloochee Mountain, 6107’, Great Smoky Mountains National Park (35.6727, -83.1762), M.S. Caterino, 5 Nov 2020 (CUAC); Swain Co.: Clingmans Dome, 6364’, Great Smoky Mountains National Park (35.5613, -83.5006), M.S. Caterino, 5 Jun 2018 (CUAC); Swain Co.: Mills Overlook, Great Smoky Mountains National Park (35.6079, -83.4380), C. Harden, 29 Sep 2020 (CUAC); Haywood Co.: Mount Sterling Trail, 5586’, Great Smoky Mountains National Park (35.6675, -83.1805), M.S. Caterino, 14 Jul 2020 (CUAC). Tennessee: Sevier Co.: Newfound Gap Rd., 4575’, Great Smoky Mountains National Park (35.6237, -83.4163), M.S. Caterino, F. Etzler, 12 Mar 2020 (2, CUAC); Sevier Co.: Mount LeConte, 6467’, Great Smoky Mountains National Park (35.6529, -83.4378), M.S. Caterino, 25 Jun 2019 (CUAC); Sevier Co.: Mount LeConte, 6571’, Great Smoky Mountains National Park (35.6542, -83.4363), M.S. Caterino, 25 Jun 2019 (2, CUAC); Sevier Co.: Indian Gap, 5500’, Great Smoky Mountains National Park, H. & A. Howden, 26 Apr 1956 (CNC).

##### Diagnosis.

This species can be distinguished from its close relative, *L.balsamense*, only by their aedeagi. In *L.smokiense*, the entire aedeagus is well sclerotized, whereas those of *L.balsamense* are more typical for the genus with distinct ventral processes (Fig. 9E, F vs Fig. 10D, E). Females can only be identified by geography, association with males, or DNA.

*Lathrobiumsmokiense* can be distinguished from *L.shermani* by the same characters given for *L.balsamense*.

##### Description.

External morphology is identical to *L.balsamense* but differs in the aedeagus. Aedeagus (Fig. 9E, F) with median lobe entirely sclerotized, sides meeting at a seam on dorsal side to produce a tube; dorsal plate small and distal; internal sac without spines.

##### Etymology.

The specific name refers to the Great Smoky Mountains where the species was first collected and is most abundant.

##### Distribution and ecology.

*Lathrobiumsmokiense* inhabits spruce-fir forests above 1500 m in the Great Smoky Mountains (Fig. 13). It is not found north of the French Broad River basin (Asheville depression), where its microhabitat is inhabited by *L.lividum* and *L.islae*. It can be collected from leaf litter but is most often beneath bryophyte mats on boulders. One specimen was collected from wildcat dung. Collected May–Sep.

#### ﻿ Lathrobium (Abletobium) solum

Taxon classificationAnimaliaColeopteraStaphylinidae

Haberski & Caterino sp. nov.

88E55E2C-34C3-5E78-8ADC-FD6D94F652ED

https://zoobank.org/981FEB2B-311A-41A0-BAE6-85B55B380793

##### Type material.

***Holotype*** ♂ (FMNH): “USA: Virginia: Botetourt Co., Jefferson NF, 0.25mi NW Blackhorse Gap parking, 37.42796N, 79.7558W, 2425ft., 24.May–19.July-2019, C.W Harden, Buried pipe trap baited with cheese, in gravelly soil, deciduous wooded rocky gully. BHG-02” / “CLEMSON ENT [QR CODE] CUAC000185155”. ***Paratypes*** (6, VMNH, CUAC, FMNH): 6: same data as holotype (CUAC000185154, CUAC000177139, CUAC000177140, CUAC000177141, CUAC000177142, CUAC000177143).

##### Diagnosis.

**Figure 21.**
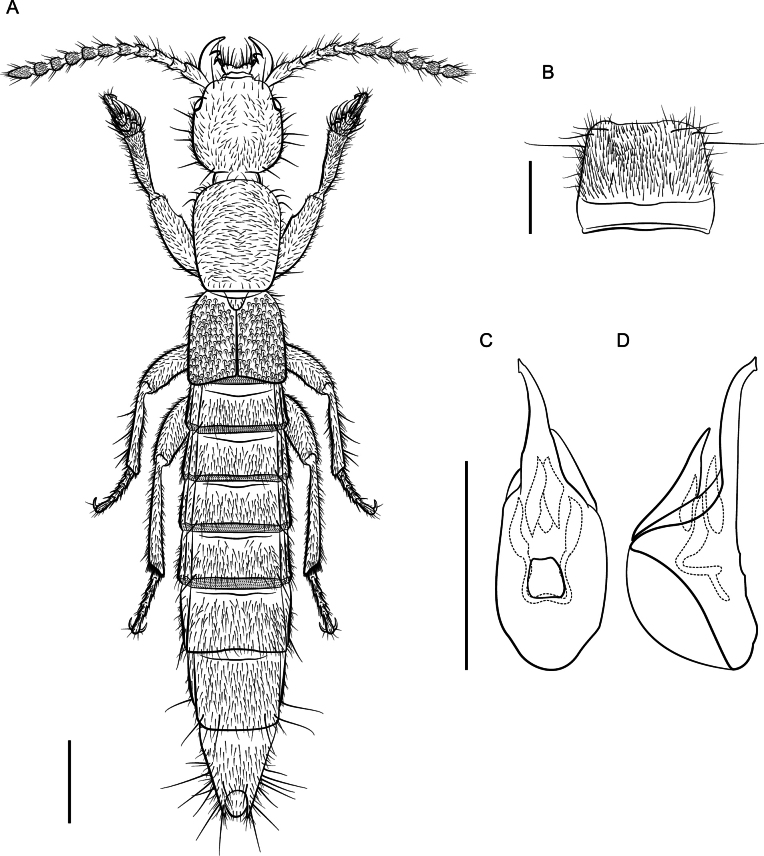
*Lathrobiumsolum***A** habitus **B** male sternite VIII **C** aedeagus in ventral view **D** aedeagus in lateral view. Scale bars: 1 mm.

Can be distinguished from all other microphthalmous *Lathrobium*, except *L.absconditum*, by its large size and unadorned male sternite VIII. It differs from *L.absconditum* in its transverse middle antennomeres and widely separate gular sutures but is most easily recognized by the aedeagus. *Lathrobiumsolum* has a distinct stirrup-shaped major spine in the internal sac, and the ventral process reaches the dorsal plate in lateral view, unlike in *L.absconditum* (Fig. 21C).

##### Description.

Habitus (Fig. 21A). Large species, total body length ~ 9 mm long, FL 3.5 mm long. Coloration: body and appendages pale reddish, distal segments of antennae lighter.

Similar to *L.hardeni*, except gular sutures straight and widely separated, 1/8 width of head but narrowing slightly posteriorly. Eyes reduced to small white membranes without ommatidia, occupying 1/10 lateral width of head. Antennomeres II–IV elongate, gradually widening so that antennomeres V–IX are as long as wide. Pronotum without a visible line at midline; interstices shiny with no microsculpture. Posterior margin more sinuate.

♂: Posterior margin of sternite VIII with a broad, shallow emargination (Fig. 21B). Aedeagus 1.5 mm long; ventral process long, reaches dorsal plate, apex produced in an asymmetrical trunk (Fig. 21C, D); dorsal plate short and diamond-shaped; internal sac with five more-or-less symmetrical spines, two largest spines connected by a thin stirrup.

♀: Female unknown.

##### Etymology.

The specific name is a play on words. In Latin *solum* can mean soil but also “lonely.” This species is hypogean, and the males are alone until a female is found.

##### Distribution and ecology.

*Lathrobiumsolum* is known only from the type locality in Botetourt County, Virginia (Fig. 18), where it was collected from a rocky gully in deciduous forest. All specimens were collected with buried pipe traps, suggesting they are hypogean. Collected May–July.

#### ﻿ Lathrobium (Abletobium) thompsonorum

Taxon classificationAnimaliaColeopteraStaphylinidae

Haberski & Caterino sp. nov.

151CFB32-897D-598B-A523-A6219748D0F6

https://zoobank.org/E7FCB1CA-BEB0-4AD4-AC24-5722F61A29C6

##### Type material.

***Holotype*** ♂ (FMNH): “USA: KY: Monroe Co., 36.6579°N, 85.6259°W, Hestand, Thompson Ln., C.W.Harden, 28.v–3.ix.2022, Buried pipe trap, house-03” / “CLEMSON ENT [QR CODE] CUAC000185201”. ***Paratypes* (12, CUAC, CNCI, and FMNH)**: 1: same data as holotype (CUAC000177144); 1: same locality as type, 25.ii–8.v.2021 (CUAC000169037, DNA Extract MSC-7054); 5: same locality as type, 22.xii.2022–19.iv.2023; 5: same locality as type, 19.iv–17.vi.2023.

##### Other material.

Kentucky: same data as holotype, CUAC000187892 (1 larva, CUAC).

##### Diagnosis.

**Figure 22.**
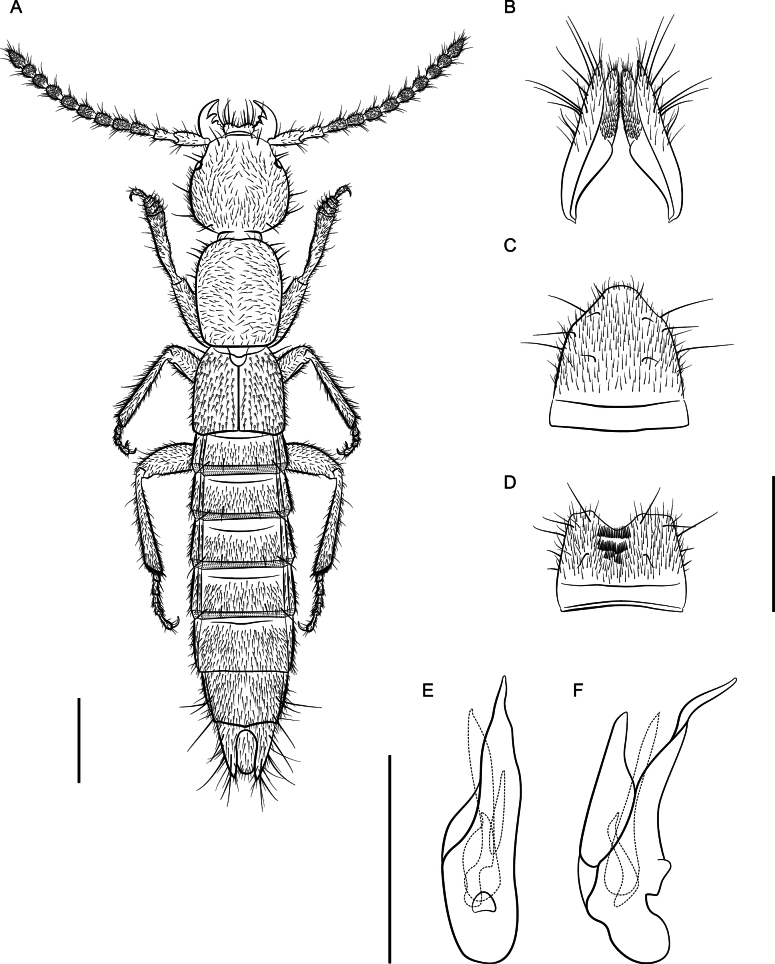
*Lathrobiumthompsonorum***A** habitus **B** female terminalia **C** female sternite VIII **D** male sternite VIII **E** aedeagus in ventral view **F** aedeagus in lateral view. Scale bars: 1 mm.

*Lathrobiumthompsonorum* is the only microphthalmous *Lathrobium* known from west of the Appalachian Plateaus. Males can be distinguished by the unique, twisted ventral process of the aedeagus (Fig. 22E, F). Females have valvifers and coxites fully divided, which is unique among microphthalmous species in the Nearctic.

##### Description.

Habitus (Fig. 22A). Mid-size species, total body length ~ 7.5 mm long, FL 3.0 mm long. Coloration: body and appendages pale reddish.

Similar to *L.hardeni*, except posterior angles of head more strongly rounded; gular sutures straight, separated by 1/10 width of head but narrowing slightly posteriorly. Eyes reduced to small white membranes without ommatidia, occupying 1/7 lateral width of head. Antennomeres II–IX obconic and longer than wide, V–VII twice as long as wide; apical antennomere longer, subacute. Pronotum with punctures spaced one diameter apart. Posterior margin of elytra more sinuate.

♂: Posterior margin of sternite VIII with rounded emargination, wider than deep; armed with two transverse combs of ~ 20, and one comb of 10 thick black setae (Fig. 22D). Aedeagus 1.4 mm long; ventral process long, asymmetrical, distal end twisted in lateral view (Fig. 22E, F); dorsal plate broad, distal end narrowing to curved point; internal sac with three spines, major spine longer than dorsal plate, 2× length of minor spines.

♀: Sternite VIII oblong (Fig. 22C); paraprocts undivided anteriorly, apices longer than basal portion; proctiger conical; sternum IX with coxites and valvifers fully divided, equal in length (Fig. 22B), coxite narrower and setose, valvifer sinuate and glabrous; subgenital plate absent.

**Figure 23.**
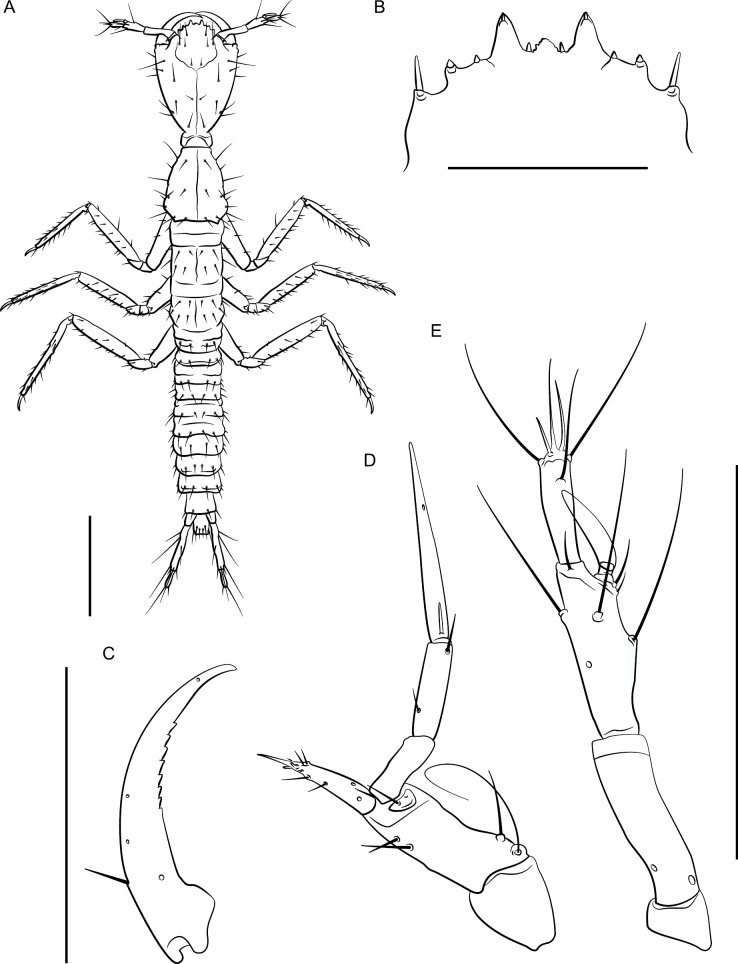
*Lathrobiumthompsonorum* first instar larva **A** habitus **B** nasale **C** mandible **D** maxilla **E** antenna. Scale bars: 1 mm (**A**); 250 μm (**B**); 500 μm (**C, D, E**).

***First instar larva***: Body elongate, ~ 5 mm long; lightly sclerotized (Fig. 23A), head more sclerotized; head light brown, legs and body white, translucent.

Head ovate, strongly tapered posteriad (in dorsal view), dorsoventrally flattened, 1.3× as long as wide, dorsal setae as in Fig. 23A; head 3.6× wider than neck; dorsal ecdysial lines bifurcate 2/5 distance between neck and nasale margin; stemmata absent; anterior margin of nasale (Fig. 23B) as in *L.hardeni*, but median triangular tooth with edge serrated; Apotome of gula reaching tentoral pits.

Antennal (Fig. 23E) length ratios: 1.0:3.1:3.1:2; antennomere I triangular; antennomere II with two pores; antennomere III with three elongate macrosetae, three solenidia, one pore; antennomere IV club-shaped with apical solenidia; sensory appendage 0.8× as long as antennomere IV.

Mandibles (Fig. 23C) long, falciform, serrate along middle 1/3 of inner margin, with a single seta near base on outer ventral edge. Maxilla (Fig. 23D) with cardo triangular; stipes elongate, 1.6× longer than cardo; mala digitiform, tapering toward apex, 1.1× as long as palpomere I, with apical sensory appendages and two pores; palpifer with one seta. Maxilla as in *L.hardeni*, but palpomere length ratios: 1.0:1.2:2.5. Labium with prementum quadrate, basal 2/3 strongly sclerotized; ligula with elongate membranous apex, 4× as long as wide, densely fimbriate, separated from prementum by a lightly sclerotized transverse strip; palpomere I 1.4× as long as II; palpomere II bearing short sensilla at apex.

Dorsal sclerites of thorax with ecdysial lines along midline of body; prothorax narrow, 1.2× as long as wide, narrowed anteriorly, chaetotaxy as in Fig. 23A; thoracic tergite II subquadrate; tergite III wider than long; abdominal sclerites lightly sclerotized, with two small pleural sclerites per segment on each side; basal segment of urogomphus 3× as long as terminal segment, with seven prominent lateral setae; terminal segment of urogomphus slender, with one short and one long apical setae.

##### Etymology.

Named in honor of the Thompson family, who own the property where this species was discovered and graciously allowed it to be collected.

##### Distribution and ecology.

*Lathrobiumthompsonorum* is known only from the type locality in Monroe County, Kentucky (Fig. 18). All specimens were collected with buried pipe traps, suggesting they are hypogean. Adult and larvae were collected in May, found in the same traps.

**Subgenus Apteralium Casey, 1905: 70.**

#### ﻿ Lathrobium (Apteralium) brevipenne

Taxon classificationAnimaliaColeopteraStaphylinidae

LeConte, 1863

9DE99791-F316-5F53-888F-EFC06B2CC5D3

Lathrobium brevipenne LeConte, 1863: 44.
Apteralium brevipenne : Casey 1905: 78.

##### Type material.

***Lectotype***, *Lathrobiumbrevipenne* LeConte, herein designated (MCZ): “[handwritten] *L.brevipenne* Lec. / Ill. / [red] Type 6447 / Lectotype *Lathrobiumbrevipenne* LeConte Desg. Haberski & Caterino.”

##### Other material.

USA: Arkansas: Logan Co.: 1 km E Lookout, Mt. Magazine, 23 May 1986, J. M. Campbell, oak-hickory leaf litter (11, CNC); Logan Co.: Brown Springs, Mt. Magazine, 23 May 1986, J. M. Campbell, leaf litter edge of stream (1, CNC); Logan Co.: Lookout, Mt. Magazine, 26 May 1986, J. M. Campbell, deciduous leaf litter (15 CNC); same locality, 14 Nov 2021, A. Haberski, P. Wooden (1 CUAC); Logan Co.: Cameron Bluffs, Mt. Magazine, 27 May 1986, J. M. Campbell, leaf litter at base of cliff (4 CNC); Logan Co.: Cove Lake Campground, 27 May 1986, J. M. Campbell (2 CNC); Stone Co.: Blanchard Springs St. Park, 18 May 1973, J. M. Campbell (1, CNC): same locality, 13 Nov 2021, A. Haberski, P. Wooden, litter on rocky outcrop (1, CUAC); Stone Co.: Sylamore Creek near Gunner Pool Recreation Area, 21 May 2017, C. Harden, litter in dry run (1 CUAC); Franklin Co.: FSR 1510, near Ozark Highland Trail, 21 May 1986, J. M. Campbell (6 CNC); Franklin Co.: Ozark Highlands Trail, 22 May 2017, C. Harden, active at night (1 CUAC); Franklin Co.: Gray Springs, Ozark National Forest, 19 May 1986, J. M. Campbell, leaf litter edge of stream (2 CNC); Pulaski Co.: Pinnacle Mountain State Park, 11 May 1986, J. M. Campbell, flood debris on bank (1 CNC); Washington Co.: Lake Wedington area, 19 May 2017, C. Harden, litter in woods above lake (1 CUAC); Cross Co.: Village Creek State Park, 13 Nov 2021, A. Haberski, P. Wooden (1 CUAC). Illinois: Union Co.: Pine Hills Field Station, 15 May 1967, J. M. Campbell (2 CNC); same locality, 19 May 1967 (1 CNC); St. Clair Co.: 01 Aug 1967, G. W. Bock (1 CNC). Indiana: ‘In.’ (1 USNM). Iowa: ‘Ia.’ (5 USNM). Missouri: Boone Co.: Columbia, 02 May 2011 (1 CNC); Oregon Co.: Surprise Sinkhole Cave, 8.5 mi NE Alton, 26 Dec 1979, J. E. Gardener (1 CNC); St. Louis Co.: Clayton, 21 June 1983, B. F. & J. L. Carr (3 CNC).

##### Diagnosis.

Both *L.camplyacra* and *L.carolinae* share a similar gestalt with *L.brevipenne*, but they are endemic to the southern Appalachian Mountains, so their ranges do not overlap. *Lathrobiumbrevipenne* can be distinguished from either species by genitalia. Its aedeagus is highly variable but is never fully sclerotized as in *L.camplyacra* and *L.carolinae*. Female genitalia of *L.brevipenne* are also variable, but all have larger and more sclerotized subgenital plates.

##### Description.

**Figures 24–26.**
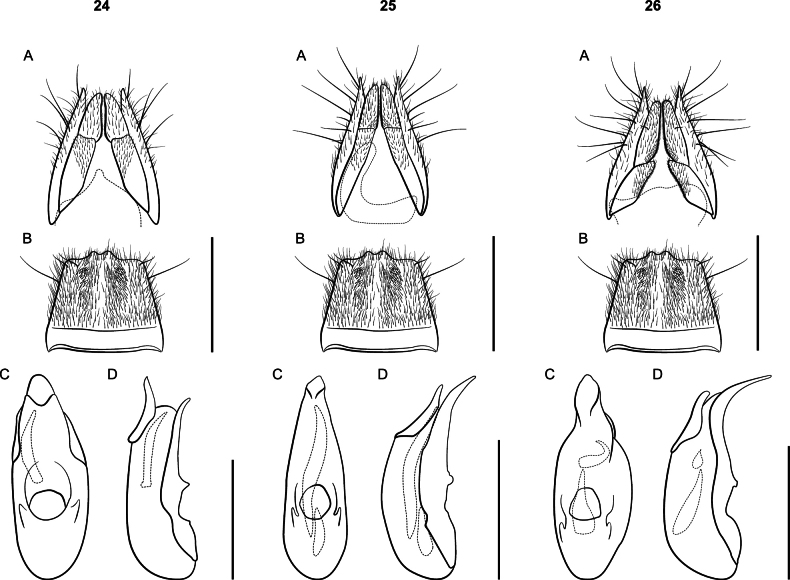
*Lathrobium* terminalia **A** female terminalia **B** male sternite VIII **C** aedeagus in ventral view **D** aedeagus in lateral view. **24***L.brevipenne* morphotype 1 **25***L.brevipenne* morphotype 2 **26***L.brevipenne* morphotype 3. Scale bars: 1 mm.

Large species, body length 9 mm; body coloration dark red, appendages lighter. Eyes small; elytra shorter than pronotum. Females with paraprocts undivided, apices shorter than basal portion; sternite VIII strongly oblong. *Lathrobiumbrevipenne* which has several allopatric populations with distinct genitalic morphotypes and is likely a species complex (Figs 24–26). All morphotypes have a subgenital plate.

##### Distribution.

USA: **AR**, IL, IN, IA, MO (Casey 1905).

#### ﻿ Lathrobium (Apteralium) camplyacra

Taxon classificationAnimaliaColeopteraStaphylinidae

Haberski & Caterino sp. nov.

1D03567D-3361-519E-9AD5-F05F476321B4

https://zoobank.org/74B8FA6C-392A-4D7E-985D-D370B075D6C5

##### Type material.

***Holotype*** ♂ (FMNH): “USA: NC: McDowell Co., 35.2784°N, 82.8008°W, Horse Cove Gap Trail, vii.23.2015, S. Myers, sifted litter.” / “Caterino DNA voucher, Ext. MSC-7059” / “CLEMSON ENT [QR CODE] CUAC000168998”. ***Paratype* (11, CUAC, FMNH)**: 8: same data as holotype (CUAC000168999, CUAC000177162, CUAC000177163, CUAC000177164, CUAC000177165, CUAC000177166, CUAC000177167, CUAC000177168). 3: same locality as type, 35.2782°N, 82.8018°W.

##### Other material.

North Carolina: Jackson Co.: Balsam Mountain Preserve, S. Myers, 15 Jun–7 Jul 2015 (20, CUAC, FMNH, CNC); Transylvania Co.: Courthouse Falls Trail, 3385’ Nantahala National Forest (35.2716, -82.8964), S. Myers, 23 Jul 2015 (CUAC); Jackson Co.: Cowee Bald, 4926’, Nantahala National Forest (35.3270, -83.3361), M.S. Caterino, 9 Jul 2019 (2, CUAC); Jackson Co.: Cowee Bald, 4931’, Nantahala National Forest (35.3270, -83.3359), M.S. Caterino, 15 Sep 2020 (CUAC); Jackson Co.: Dark Ridge, 3467’, Nantahala National Forest (35.3980, -83.1088), S. Myers, 19 Jun 2015 (CUAC); Jackson Co.: Sugarloaf Mountain, 4576’, Nantahala National Forest (35.3699, -83.1212), S. Myers, 15 Jun 2015 (CUAC); Transylvania Co.: Toxaway Mountain, 4746’, Nantahala National Forest (35.1321, -82.9842), M.S. Caterino, 13 Oct 2020 (CUAC); Jackson Co.: 6 km S Cashiers, 3198’ Nantahala National Forest (35.085, -83.1015), A. Smetana, 20 Jun 1986(3, CNC); Jackson Co.: Whiteside Mountains, 4494–4921’, Nantahala National Forest (35.0817, -83.13846), A. Smetana, 21 May 1986 (5, CNC). South Carolina: Oconee Co.: East Fork Chattooga River, Sumter National Forest (34.9843, -83.0981), S. Myers, 4 May 2015 (2, CUAC); Oconee Co.: Indian Camp Creek, Sumter National Forest (34.9899, -83.0724), S. Myers, 4 May 2015 (5, CUAC). Tennessee: Sevier Co.: Newfound, 5000’, Great Smoky Mountains National Park (35.6127, -83.4246), A. Smetana, 09 Jun 1982 (CNC).

##### Diagnosis.

This species can be distinguished from the closely related *L.carolinae* only by the genitalia. Aedeagi are similar, but those of *L.camplyacra* have bent ventral processes. The terminalia of female *L.camplyacra* have partially sclerotized subgenital plates and are longer and narrower than those of *L.carolinae*, with relatively shorter coxites. No intermediate forms are known.

Both *L.camplyacra* and *L.carolinae* share a similar gestalt with *L.brevipenne*. The latter is endemic to the Interior Highlands of the south-central United States. *Lathrobiumbrevipenne* can be distinguished from either species by genitalia. Its aedeagus is highly variable in shape but is never fully sclerotized (Figs 24–26). The females of *L.brevipenne* have larger, more sclerotized subgenital plates.

##### Description.

External morphology (Fig. 5A) identical to *L.carolinae*. It differs only in the sexual characters.

♂: Aedeagus (Fig. 5E, F) with median lobe well sclerotized; ventral process strongly curved, distal tip lying beyond median foreman in lateral view; dorsal plate long; internal sac with three patches of rugose flagella, visible as one structure when invaginated.

♀: Apical lobes of paraproct shorter than continuous basal portion in dorsal view; proctiger conical; sternum IX with coxites and valvifers fully divided, coxites less than ½ length of valvifers (Fig. 5B); subgenital plate a lightly sclerotized chevron.

##### Etymology.

The specific name is from the Greek *camplyo*- meaning bent, and *acra* meaning tip, in reference to the bent ventral process of the aedeagus.

##### Distribution and ecology.

**Figure 27.**
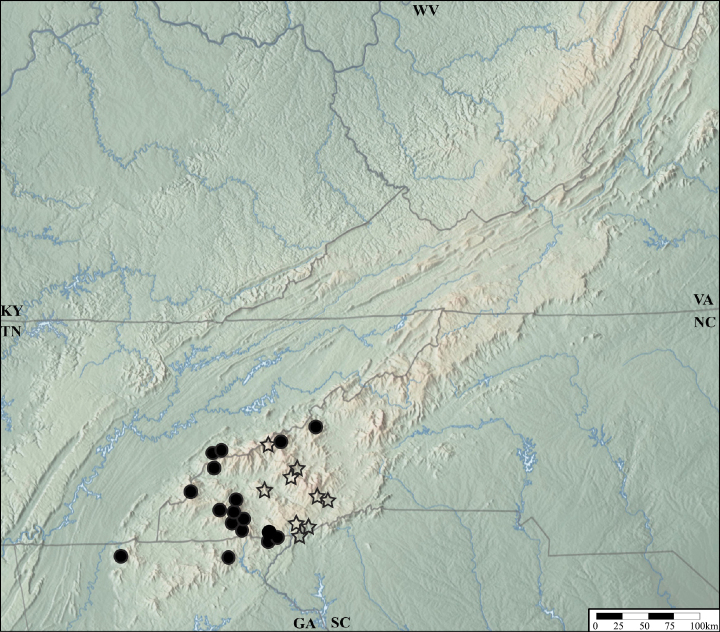
Distribution of *L.camplyacra* (star), *Lathrobiumcarolinae* (circle).

*Lathrobiumcamplyacra* inhabits mid-elevation (600–1600 m) hardwood forests in the Great Balsam, Plott Balsam, and Cowee Mountains of the southern Appalachians (Fig. 27). This area is bordered by the Cullasaja River and Little Tennessee River to the west, the Maggie Valley to the north, and the French Broad River Valley to the east. A single specimen from the CNC collection was reportedly collected from Newfound Gap in the Great Smoky Mountains, which is well outside of this range and needs to be verified.

#### ﻿ Lathrobium (Apteralium) carolinae

Taxon classificationAnimaliaColeopteraStaphylinidae

(Casey, 1905)

CE6442B1-B553-5981-AA72-A1C5A150F7DF

Apteralium carolinae Casey, 1905: 78.
Lathrobium (Apteralium) carolinae : Bernhauer and Schubert 1912: 257.

##### Type material.

***Lectotype***, *Apteraliumcarolinae* Casey, herein designated (USNM): “♂ / Highlands NC, Jun 6 88 / CASEY bequest 1925 / [red] *carolinae* – 2, TYPE USNM 38105 / Lectotype *Apteraliumcarolinae* Casey Desg. Haberski & Caterino.”

##### Other material.

USA: Georgia: Rabun Co.: Satoloh, 13 Jun 1957, R. M. Mason (3, CNC); Rabun Co.: Rabun Bald, 11 May 2021, M. Caterino (1, CUAC); Dillard Co.: 15 Apr 1948, F. Rapp (1, CNC); Union Co.: Brasstown Bald, 02 Jul 2020, M. Caterino (1, CUAC); same locality, 17 Sep 2020 (1, CUAC); Union Co.: Little Bald Mountain, 02 July 2020, M. Caterino (2, CUAC). North Carolina: same data as holotype (1, USNM); Macon Co.: Hwy 64 near Dry Falls NW Highlands, 16 May 1986, A. Smetana (1, CNC): Macon Co.: near Cliffside Lake Campground NW highlands 16 May 1986, A. Smetana (3, CNC); Macon Co.: Van Hook Glade, 4 mi W Highlands, 30 Aug 1967, J. M. & B. A. Campbell (1, CNC); Macon Co.: Copper Ridge Bald, 09 Jul 2019, M. Caterino (2, CUAC); Macon Co.: Forest Service Rd 77, 10 Jul 2020, A. Deczynski (1, CUAC); Macon Co.: Hickory Branch Trail, 26 Jul 2015, M. Caterino, S. Langton-Myers, hardwood litter (5, CUAC); Macon Co.: Hickory Gap, 16 Jul 2015, M. Caterino, S. Langton-Myers (1, CUAC); Macon Co.: Jones Gap, 16 July 2015, M. Caterino, S. Langton-Myers, litter around bracket fungus (8, CUAC); same data, except 22 July 2015, hardwood litter against logs (4, CUAC); Macon Co.: Wayah Bald Rd, 18 Apr 2020, C. Harden, under rock by seep (1, CUAC); Clay Co.: Chunky Gal Trail, 01 Sep 2020, M. Caterino, S. Langton-Myers (1, CUAC); Clay Co.: Riley Knob, 11 May 2020, M. Caterino (1, CUAC); Clay Co.: Shooting Creek Bald, 11 May 2020, M. Caterino (1, CUAC); Caly Co.: Tusquitee Bald, 06 Jul 2021, M. Caterino (1, CUAC); Cherokee Co.: London Bald Trail, 26 Jul 2015, M. Caterino, S. Langton-Myers, hardwood litter (4, CUAC); Graham Co.: Cherohala Skyway, 27 Sep 2020, C. Harden (1, CUAC); Graham Co.: Huckleberry Knob, 04 May 2020, M. Caterino (1, CUAC); same data, except 13 Oct 2020 (2, CUAC); Joyce Kilmer Memorial Forest, 24 Jun 2015, M. Caterino, S. Langton-Myers (8, CUAC); same data, except 20 Jul 2015 (7, CUAC); Graham Co.: Teyahalee Bald, 09 Jul 2019, M. Caterino (1, CUAC); Haywood Co.: Balsam Mountain Trail, 05 Sep 2020, M. Caterino (1, CUAC); Haywood Co.: Pisgah National Forest, 14 July 2020, A. Deczynski (1, CUAC); Swain Co.: Miller Cove Trail, 20 July 2015, M. Caterino, S. Langton-Myers (1, CUAC); Swain Co.: Smokemont Campground, 10 km N Cherokee, 09 Jun 1982, A. Davies (9, CNC); Swain Co.: Lakeshore Trail, Great Smoky Mountains National Park, 18 July 2003, A. Tishechkin (2, LSAM); Transylvania Co.: Twentymile Trail near Twentymile Creek, 19 Oct 2007, I. M. Sokolov, litter on rock explosion (1, LSAM). Tennessee: Blount Co.: Cades Cove, Great Smoky Mountains National Park, Jun 1954, H. Howden (1, CNC); Blount Co.: Greenbrier Cove, Great Smoky Mountains National Park, 27 Jun 2001, C. Carlton, A. Tishechkin, V. Moseley (1, LSAM); Monroe Co.: Indian Boundary Camp, 28 May 2020, C. Harden (1, CUAC).

##### Diagnosis.

This species can be distinguished from *L.camplyacra* only by the genitalia. Aedeagi are similar but the ventral processes of *L.carolinae* are straight, not bent as those of *L.camplyacra*. The terminalia of female *L.carolinae* lack subgenital plates and are shorter and wider than those of *L.camplyacra*. No intermediate forms are known.

**Figure 28.**
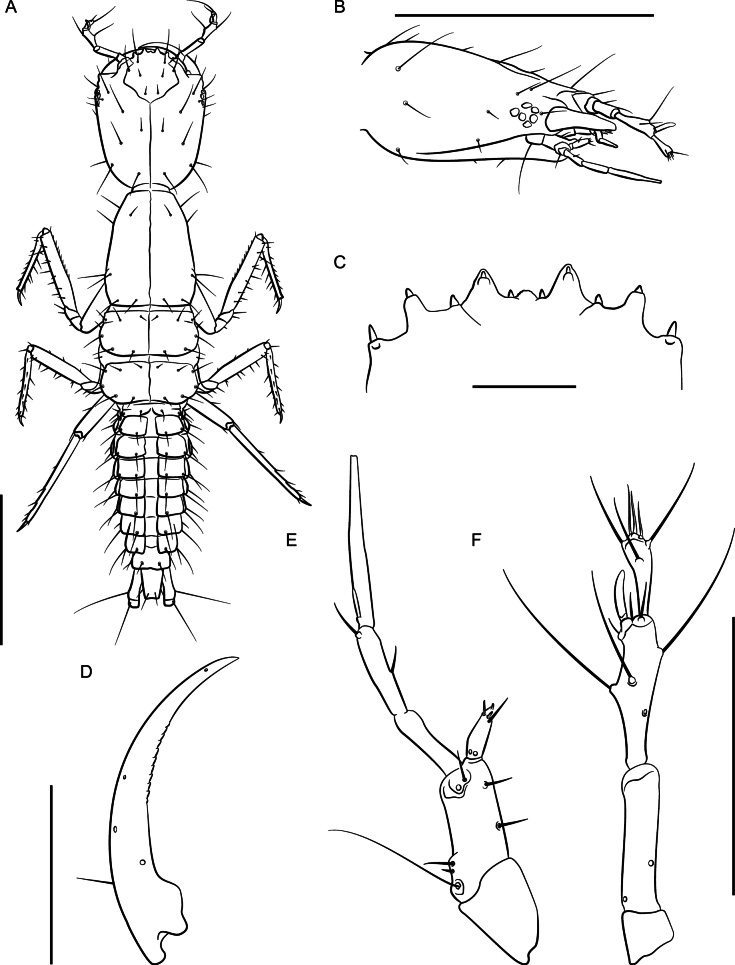
*Lathrobiumcarolinae* second instar larva **A** habitus **B** head in lateral view **C** nasale **D** mandible **E** maxilla **F** antenna. Scale bars: 1 mm (**A, B**); 100 μm (**C**); 250 μm (**D, E, F**).

*Lathrobiumcarolinae* can be distinguished from *L.brevipenne* by the same characters given for *L.camplyacra*.

##### Description.

The original description lacked a description of the genitalia. We present descriptions and illustrations of the male and female genitalia, as well as the first description of the mature larva.

♂: Aedeagus 1.7 mm long (Fig. 4D, E), median lobe well sclerotized; ventral process relatively straight, distal tip not bending beyond median foreman in lateral view; dorsal plate long; internal sac with three patches of rugose flagella, visible as two structures when invaginated.

♀: Paraprocts undivided anteriorly, apices shorter than basal portion; proctiger conical; sternum IX with coxites and valvifers fully divided, coxites ¾ length of valvifers (Fig. 4A); subgenital plate absent.

***Second instar larva***: Larvae were associated with adults by DNA barcoding. Body elongate, ~ 5 mm long; well sclerotized (Fig. 28A); head, thoracic, and abdominal tergites brown, appendages light yellow, intersegmental membrane white, translucent.

Head ovate, widest at stemmata and slightly tapered posteriad (in dorsal view), dorsoventrally flattened, 1.3× as long as wide; dorsal setae as in Fig. 28A; head 2.9× as wide as neck; dorsal ecdysial lines bifurcate 3/8 distance between neck and nasale margin; six stemmata present, arranged as in Fig. 28B; anterior margin of nasale (Fig. 28C) with nine blunt teeth pointing anteriorly, one round median tooth with edge serrated, a pair of paramedian teeth, and three pairs of lateral teeth; innermost lateral teeth are small and indistinct; paramedian and lateral teeth armed with nodular setae, and a pair of nodular setae separate median and paramedian teeth; Apotome of gula reaching tentoral pits.

Antennae (Fig. 28F) length ratios: 1.0:2.7:2.8:1.5; antennomere I triangular; antennomere II with two pores; antennomere III with three elongate macrosetae, three solenidia, one pore, and a curved sensory appendage 2/3 as long as terminal antennomere; antennomere IV club-shaped with apical solenidia; sensory appendage 0.6× as long as antennomere IV.

Mandibles (Fig. 28D) long, falciform, serrate along middle 1/3 of inner margin, with a single seta near base on outer ventral edge. Maxilla (Fig. 28D) with cardo triangular; stipes elongate, 1.3× longer than cardo; mala digitiform, tapering toward apex, 0.7× as long as palpomere I, with apical sensory appendages and two pores; palpifer with one seta. Maxillary palpomere length ratios: 1.0:1.2:2.2; palpomere II with two setae; palpomere III with one basal sensory appendage and numerous small apical appendages. Labium with prementum trapezoidal, basal ¾ strongly sclerotized; ligula with elongate membranous apex, 3× as long as wide, densely fimbriate, separated from prementum by a distinctly sclerotized transverse strip; palpomere I 1.8× as long as II; palpomere II bent near apex and bearing short sensilla at apex.

Dorsal sclerites of thorax with ecdysial lines along midline of body (Fig. 28A); prothorax narrow, 1.3× as long as wide, widest posteriorly and narrowing anteriorly, chaetotaxy as in Fig. 28A; thoracic tergites II and III subequal in width and length; abdominal sclerites well sclerotized, with two small pleural sclerites per segment on each side; urogomphi broken off of only known specimen.

##### Distribution and ecology.

*Lathrobiumcarolinae* inhabits mid-elevation (600–1600 m) hardwood forests in the Appalachian Mountains, from Georgia to the French Broad River basin in North Carolina (Fig. 28), except in those ranges occupied by *L.camplyacra* as described above. Collected Feb–Oct.

**Subgenus Lathrobioma Casey, 1905: 72.**

#### ﻿ Lathrobium (Lathrobioma) divisum

Taxon classificationAnimaliaColeopteraStaphylinidae

LeConte, 1880

11EC6692-26B3-5285-9E93-846DA7803358

Lathrobium divisum LeConte, 1880: 176.
Lathrobium franciscanum Casey, 1905: 84. New synonym.

##### Type material.

***Lectotype***, *Lathrobiumdivisum* LeConte, herein designated (MCZ): “Vanc. / [handwritten] *L.divisum* Lec. / COLLECTION / TYPE [handwritten] *divisum* / [red] Type 6453 / Lectotype *Lathrobiumdivisum* LeConte Desg. Haberski & Caterino.” ***Lectotype***, *Lathrobiumfranciscanum* Casey, herein designated (USNM): “LosGatos CAL/ CASEY bequest 1925 / [handwritten] *Los Gatos is in Santa Clara Co. not far from Sta. Cruz California* / [red] TYPE USNM 38114 / Lectotype *Lathrobiumfranciscanum* Casey Desg. Haberski & Caterino.”

##### Other material.

USA: California: Santa Clara Co.: Los Gatos (1, USNM); Mendocino Co.: Gualala (1, USNM); Illinois (1, MCZ).

##### Diagnosis.

This species can be distinguished from other *Lathrobioma* by its large size. Additionally, males are the only *Lathrobioma* to lack an emargination on sternite VIII and have an asymmetrical aedeagus. Females have paraprocts subequal in length to the basal portion of tergite IX, while other species have paraprocts that are short or fully divided.

##### Description.

**Figures 29–34.**
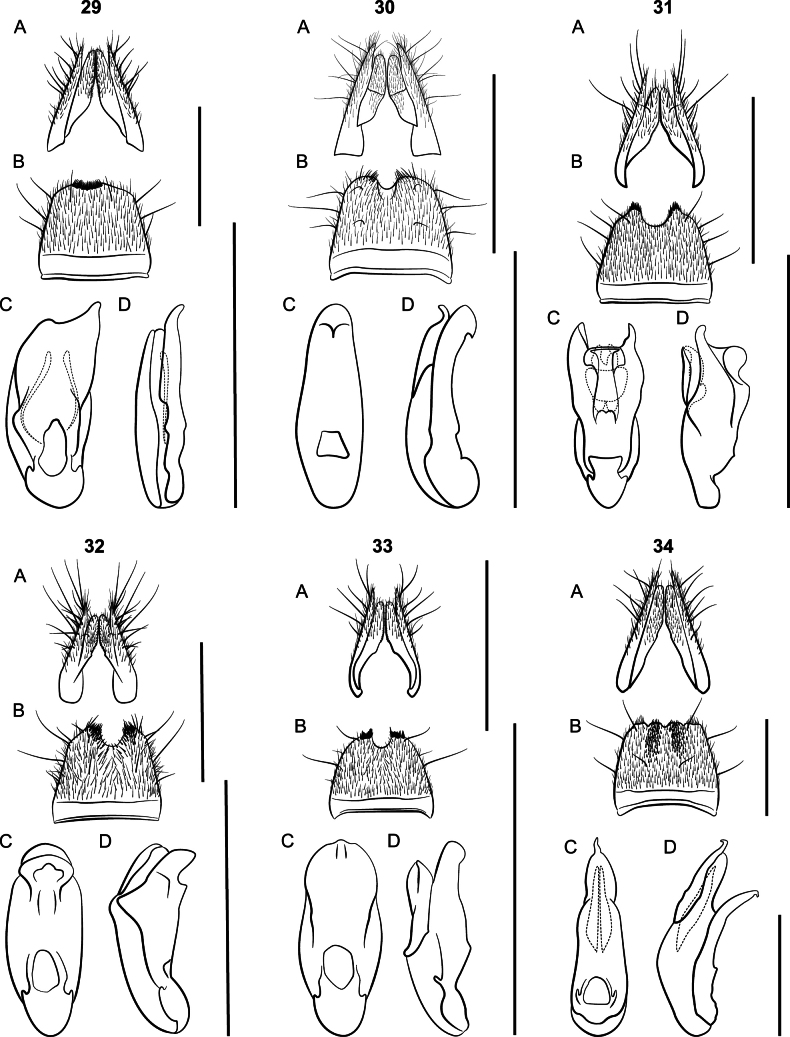
*Lathrobium* terminalia **A** female terminalia **B** male sternite VIII **C** aedeagus in ventral view **D** Aedeagus in lateral view. **29***L.divisum***30***L.nanulum***31***L.othioides***32***L.scolopaceum***33***L.tenue***34***L.amplipenne*. Scale bars: 1 mm.

Large species, body length 7–8 mm. Body coloration red, elytra bicolored, appendages lighter yellow. Gular sutures arcuate; antennomeres V–VII as long as wide. Elytra as long as pronotum. Females with paraprocts undivided, apices as long as basal portion; sternite VIII weakly oblong. Male sternite VIII without emargination, thick black setae at apex; genitalia as in Fig. 29.

##### Distribution.

Canada: BC. USA: CA, **IL**, OR, WA (Newton 2022).

##### Remarks.

*Lathrobiumfranciscanum* is reduced to synonymy with *Lathrobiumdivisum* because the distinguishing characters given by Casey (1905) were insufficiently distinctive. *Lathrobiumfranciscanum* was described from one female and one male, which were differentiated from *L.divisum* based on subtle morphological differences in somatic characters. Its body was supposedly “more slender,” punctures “somewhat sparser,” head “not so large,” and prothorax “slightly narrower” than the head as opposed to “much narrower” in *L.divisum* (Casey 1905). These differences are difficult to see and fall within the range of intraspecific variation in longer series of other *Lathrobium*. We examined the genitalia of both species and found no differences in the shape of their aedeagi.

Casey (1905) placed *L.divisum* in *Lathrobium* s. str., but after examining the types, we transfer it to *Lathrobioma* based on the following synapomorphies: metatarsi compact, tarsomeres I–IV subequal in length, each ~ 1/3 as long as fifth tarsomere; maxillary palpomere III more than 0.4× as wide as long; and male sternite VIII with bristles positioned apically. The final character is presented here for the first time.

#### ﻿ Lathrobium (Lathrobioma) nanulum

Taxon classificationAnimaliaColeopteraStaphylinidae

(Casey, 1905)

0A45041B-C287-512C-8988-6E86BA480A0C

Lathrobioma nanula Casey, 1905: 100.
Lathrobium (Lathrobioma) nanulum : Bernhauer and Schubert 1912: 264.

##### Type material.

***Lectotype***, *Lathrobiomananula* Casey, herein designated (USNM): “MASS / CASEY bequest 1925 / [red] TYPE USNM 38139 / [handwritten] *nanula* / Lectotype *Lathrobiomananula* Casey Desg. Haberski & Caterino.”

##### Other material.

Same data as lectotype (1, USNM).

##### Diagnosis.

*Lathrobiumnanulum* can be distinguished from other *Lathrobioma* by unique genitalic characters. The ventral process of the aedeagus is narrow instead of broad. Female sternum IX with valvifers and coxites fully divided instead of fused.

##### Description.

Small species, body length 5 mm; body and appendages dark red coloration. Gular sutures arcuate; antennomeres V–VII wider than long; maxillary palpomere III more than 0.4× as wide as long. Elytra 1.2× longer than pronotum. Females with paraprocts undivided, apices shorter than basal portion; sternite VIII oblong. Dorsal plate and ventral process of aedeagus with large apical teeth (Fig. 30).

##### Distribution.

USA: MA.

##### Remarks.

Blackwelder (1939), in his revision of the paederine genera, placed *L.nanulum* in the subgenus Deratopeus, now a synonym of the genus *Tetartopeus*, without providing any explanation. We transfer it back to *Lathrobioma*, because it lacks any of the defining characters of *Tetartopeus*, such as a narrow neck, but does share the subgeneric synapomorphies of *Lathrobioma* listed above for *L.divisum*.

#### ﻿ Lathrobium (Lathrobioma) othioides

Taxon classificationAnimaliaColeopteraStaphylinidae

LeConte, 1880

D93B94DE-5692-56E0-A15F-B2CDAEF70337

Lathrobium othioides LeConte, 1880: 175.
Lathrobioma othioides : Casey 1905: 101.
Lathrobium (Lathrobioma) inops Casey, 1885: 135; Bernhauer and Schubert 1912: 264 [in error].

##### Type material.

***Lectotype***, *Lathrobiumothioides* LeConte, herein designated (MCZ): “Mas. / ♂ / [handwritten] *L.othioides* Lec. / [red] Type 6448 / Lectotype *Lathrobiumothioides* LeConte Desg. Haberski & Caterino.”

##### Other material.

Canada: Lake Superior (2, USNM). USA: Iowa: ‘Ia.’ (1, USNM). Massachusetts: ‘Mass.’ (5, MCZ); Norfolk Co.: Brookline, 21 Mar 1899 (1, MCZ); same locality, 17 Apr 1899, C. A. Frost (1, CUAC); same data, except 09 Apr 1899 (1, MCZ); ‘Mass’ (1, USNM). New Jersey: ‘N.J.?’ (2, USNM).

##### Diagnosis.

Males have distinctive aedeagi with the apex of the ventral process divided into two projecting horns, absent in all other *Lathrobioma*. Females can be difficult to distinguish from *L.tenue*, but their gonocoxites are generally more robust and convex in the basal half.

##### Description.

Body length 6 mm; body coloration dark red, appendages lighter red. Gular sutures arcuate; maxillary palpomere III > 0.4× as wide as long; antennomeres V–VII as wide as long. Elytra as long as pronotum. Females with paraprocts undivided, apices shorter than basal portion; sternite VIII weakly oblong. Aedeagus with characteristic projections of ventral process (Fig. 31).

##### Distribution.

Canada: ON, NB, QC (Bousquet et al. 2013). USA: IA, **NJ**, MA, RI.

##### Remarks.

Bernhauer and Schubert (1912) listed *L.inops* as a synonym of *L.othioides*, but after comparing the types, we determined this was incorrect. The aedeagus of *L.othioides* is quite distinctive and that of the *L.inops* lectotype did not match it. Instead, it was indistinguishable from that of *L.scolopaceum*, with which we synonymize *L.inops* below.

#### ﻿ Lathrobium (Lathrobioma) scolopaceum

Taxon classificationAnimaliaColeopteraStaphylinidae

(Casey, 1905)

5D6865AC-7F7D-50E7-BA48-28D3F36D2DCE

Lathrobioma scolopacea Casey, 1905: 103.
Lathrobium (Lathrobioma) scolopaceum : Bernhauer and Schubert 1912: 267.
Lathrobioma dakotana Casey, 1905: 100. New synonym.
Lathrobium (Lathrobioma) dakotanum : Bernhauer and Schubert 1912: 258.
Lathrobioma virginica Casey, 1905: 99. New synonym.
Lathrobium (Lathrobioma) virginicum : Bernhauer and Schubert 1912: 269.
Lathrobioma inops Casey, 1885; Casey 1905: 103. New synonym.
Lathrobium (Lathrobioma) inops : Blackwelder 1939, 108.

##### Type material.

***Lectotype***, *Lathrobiomascolopacea* Casey, herein designated (USNM): “B MASS / CASEY bequest 1925 / [red] TYPE USNM 38143 / [handwritten] *scolopacea* / Lectotype *Lathrobiomascolopacea* Casey Desg. Haberski & Caterino.” ***Lectotype***, *Lathrobiomadakotana* Casey, herein designated (USNM): “[handwritten] Bismarck Dak / CASEY bequest 1925 / [red] TYPE USNM 38136 / [handwritten] *Lathrobiomadakotana* / Lectotype *Lathrobiomadakotana* Casey Desg. Haberski & Caterino.” ***Lectotype***, *Lathrobiomavirginica* Casey, herein designated (USNM): “Grafton WV / CASEY bequest 1925 / [red] TYPE USNM 38139 / Lectotype *Lathrobiomavirginica* Casey Desg. Haberski & Caterino.” ***Lectotype***, *Lathrobiomainops* Casey, herein designated (USNM): “L.Sup / ♂ / CASEY bequest 1925 / [red] TYPE USNM 38144 / Lectotype *Lathrobiomainops* Casey Desg. Haberski & Caterino.”

##### Other material.

Canada: Ontario: ‘L. Sup’ (1, USNM). USA: Massachusetts: ‘Mass’ (2, USNM); Middlesex Co.: Framingham, 1944, C. A. Frost (1, MCZ). Maine: Knox Co.: Isle-au-haut, Aug 1905 (1, MCZ). New Hampshire: Grafton Co.: Downes Brook, Potash Mountain, 05 Jun 2021, A. Haberski (2, CUAC); Grafton Co.: Mount Lafayette, 04 Jun 2012, A. Haberski (1, CUAC); Strafford Co.: Spruce Hole Conservation Area, D. S. Chandler (1, UNHC); Grafton Co.: Hubbard Brook Experimental Forest, D. S. Chandler (1, UNH).

##### Diagnosis.

Females of *L.scolopaceum* are readily distinguished from all other *Lathrobioma* by their divided paraprocts. Males can be distinguished by their uniquely shaped aedeagus, which has a ventrally projected apex of the ventral process.

##### Description.

Body length 6 mm; coloration reddish, appendages lighter. Gular sutures arcuate; maxillary palpomere III > 0.4× as wide as long; antennomeres V–VII as long as wide. Elytra at least as long as pronotum, sometimes longer and wider. Females with paraprocts divided; sternite VIII weakly oblong. Characteristic aedeagus as in Fig. 32.

##### Distribution.

Canada: ON, NB, NS, QC (Bousquet et al. 2013). USA: IA, MA, ME, NH, ND, RI, VA, **WV** (Newton 2022).

##### Remarks.

We reduce *Lathrobiumdakotanum*, *Lathrobiumvirginicum*, and *Lathrobiuminops* to synonymy with *Lathrobiumscolopaceum* because the distinguishing characters given by Casey (1905) were inaccurate. *Lathrobiumdakotanum* and *L.virginicum* were each described from a single female and differentiated from *L.scolopaceum* based on their elytra being at least equal in length to the pronotum, as opposed to “much shorter” (Casey 1905). We measured the specimens in Casey’s collection and found that the elytra of *L.scolopaceum* were 1.1× longer the pronotum, approximately the same as that of *L.dakotanum* and *L.virginicum*.

*Lathrobiumvirginicum* was further differentiated by having a head as wide as its elytra, as opposed to narrower in *L.scolopaceum*, *L.dakotanum*, and *L.inops*. Upon measuring, we found all four species have a head wider than their pronotum.

*Lathrobiuminops* was distinguished as being larger and more slender than *L.scolopaceum* (Casey, 1905), but this was not the case.

The genitalia are identical in all four species.

#### ﻿ Lathrobium (Lathrobioma) tenue

Taxon classificationAnimaliaColeopteraStaphylinidae

LeConte, 1863

895DEB65-4052-5CAC-9E25-6C915CD98917

Lathrobium tenue LeConte, 1863: 44.
Lathrobioma tenuis Casey, 1905: 101.
Lathrobioma hespera Casey, 1905: 100. New synonym.
Lathrobium (Lathrobioma) hesperum : Bernhauer and Schubert 1912: 261
Lathrobioma nigrolinea Casey, 1905: 102. New synonym.
Lathrobium (Lathrobioma) nigrolinea : Bernhauer and Schubert 1912: 264.
Lathrobium (Lathrobioma) nigrolineum : Blackwelder 1939: 108.
Lathrobioma oregona Casey, 1905: 102. New synonym.
Lathrobium (Lathrobioma) oregonum Bernhauer and Schubert 1912: 264.
Lathrobioma shoshonica Casey, 1905: 99. New synonym.
Lathrobium (Lathrobioma) shoshonicum Bernhauer and Schubert 1912: 267.

##### Type material.

***Lectotype***, *Lathrobiomatenuis* Casey, herein designated (MCZ): Pink Disc / “[handwritten] *L.tenue* Lec. / [red] Type 6452 / Lectotype *Lathrobiumtenue* LeConte Desg. Haberski & Caterino.” ***Lectotype***, *Lathrobiomahespera* Casey, herein designated (USNM): “Br.C/ CASEY bequest 1925 / [red] TYPE USNM 38140 / Lectotype *Lathrobiomahespera* Casey Desg. Haberski & Caterino.” ***Lectotype***, *Lathrobiomanigrolinea* Casey, herein designated (USNM): “Winnipeg Man./ CASEY bequest 1925 / [handwritten] *nigrolinea* / [red] TYPE USNM 38141 / Lectotype *Lathrobiomanigrolinea* Casey Desg. Haberski & Caterino.” ***Lectotype***, *Lathrobiomaoregona* Casey, herein designated (USNM): “Port Oreg/ CASEY bequest 1925 / [handwritten] *oregona* / [red] TYPE USNM 38142 / Lectotype *Lathrobiomaoregona* Casey Desg. Haberski & Caterino.” ***Lectotype***, *Lathrobiomashoshonica* Casey, herein designated (USNM): “Or. / ♂ / CASEY bequest 1925 / [handwritten] *shoshonica* / [red] TYPE USNM 38138 / Lectotype *Lathrobiomashoshonica* Casey Desg. Haberski & Caterino.”

##### Other material.

Canada: Ontario (3, MCZ). USA: Colorado: Alamosa (1, MCZ). Connecticut: New Haven (1, USNM). Michigan: ‘Mic.’ (1, MCZ). Oregon: ‘Oreg’ (1, USNM). Rhode Island: ‘R.I.’ (7, USNM). Virginia: Highland Co.: Water Sinks, Lucas Tract, 04 May 2019, C. Harden, gravel stream bank (1, CUAC). West Virginia: Pocahontas Co.: Cranberry Wilderness, Monongahela National Forest, 11 Jun 2019, C. Harden, under rock near stream in spruce/hardwoods (1, CUAC).

##### Diagnosis.

Males of *L.tenue* have a broadly rounded and symmetrical ventral process of the aedeagus that is unique among *Lathrobioma* spp. Females are difficult to differentiate from *L.othioides*, but their gonocoxites are narrower and concave in the basal half.

##### Description.

Body length 6 mm; coloration dark, appendages yellow or light red. Gular sutures arcuate; maxillary palpomere III > 0.4× as wide as long; antennomeres V–VII as long as wide. Elytra at least as long as pronotum, sometimes longer and wider. Females with paraprocts undivided, apices shorter than basal portion; sternite VIII weakly oblong. Ventral process of aedeagus broadly rounded (Fig. 33).

##### Distribution.

Canada: BC, MB, **ON**, QC, SK (Bousquet et a. 2013). USA: **CO**, **CT**, ID, MA, **MI**, NY, OR, RI, **VA**, WA, **WV** (Newton 2022).

##### Remarks.

We reduce *Lathrobiumhesperum*, *Lathrobiumoregonum*, *Lathrobiumnigrolinea*, and *Lathrobiumshoshonicum* to synonymy with *Lathrobiumtenue* due to insufficient morphological differences between species. *Lathrobiumshoshonicum* and *L.hesperum* were described as having elytra longer than their pronota, in contrast to *L.tenue* which supposedly had elytra shorter than their pronota. However, upon measuring the type specimen and those in Casey’s collection, we found *L.tenue* also has elytra longer than its pronotum.

*Lathrobiumoregonum* and *L.nigrolinea* were differentiated primarily on the shape of the emargination of male sternite VIII. In these species, the setae at the tips of the emargination are inflexed, rather than diverging as in *L.tenue*. This difference was based on a single male of each species and could represent normal variation, regional difference, or coincidence.

We examined the genitalia of all five species and found no differences.

**Subgenus Lathrobium s. str.**

*Litolathra* Casey, 1905: 71.

Lathrobium (Litolathra): Bernhauer and Schubert 1912: 40.

#### ﻿ Lathrobium (Lathrobium) amplipenne

Taxon classificationAnimaliaColeopteraStaphylinidae

Casey, 1905

D944862B-9CB9-5319-94F4-28FA7267BF0E

Lathrobium amplipenne Casey, 1905: 81.

##### Type material.

***Lectotype***, *Lathrobiumamplipenne* Casey, herein designated (USNM): “N. Y. / CASEY bequest 1925 / [red] TYPE USNM 38107 / [handwritten] *Lathrobiumamplipenne* / Lectotype *Lathrobiumamplipenne* Casey Desg. Haberski & Caterino.”

##### Other material.

Illinois: Union Co., 1 mi E Wolf Lake, 8.V.1976, A. Smetana (2, CNC); 2 mi NE Reynoldsville, 9.V.1976, A. Smetana (1, CNC). Indiana: Tippecanoe Co., 27.X.1956, N.M. Downie (1, CNC). Louisiana: Concordia Pa. [Park?], 5 mi W Ferriday, 1.V.1976, A. Smetana (1, CNC). Missouri: St. Louis Co., 9.V.1920, G.W. Bock (1, CNC). New York: Tompkins Co.: Ithaca Valley, (9, USNM). Pennsylvania: ‘Penn.’. F. C. Bowditch (1, MCZ). South Carolina: Charleston Co.: Ravenel, 05 May 2001, J. C. Ciegler, UV light (2, CUAC); Dorchester Co.: Bluff Trail, McAlheny Nature Preserve, Reevesville, 11 May 2013, J. C. Ciegler (1, CUAC); Marion Co.: Woodbury, 10 Sep 2011, J. C. Ciegler (1, CUAC).

##### Diagnosis.

The habitus of this species closely resembles that of *L.armatum*, *L.geminum*, *L.praelongum*, and *L.pedale*. Males differ from those species in their unique sternite VIII, which has three small emarginations at the apex and two longitudinal patches of dense setae (Fig. 34B). Females are difficult to tell apart from *L.armatum* and *L.praelongum*. Their paraprocts are slightly shorter than those of *L.armatum*, and longer than those of *L.amplipenne*, relative to the basal portion of tergite IX. Females can be easily distinguished from *L.geminum*, which has gonocoxites shorter than paraprocts, and *L.pedale*, which has valvifers and coxites divided.

##### Description.

Large species, body length 10 mm; body coloration dark, appendages lighter red, elytra bicolored. Gular sutures converging, nearly touching posteriorly; antennomeres V–VII longer than wide. Elytra 1.3× as long as pronotum. Females with paraprocts undivided, apices shorter than basal portion; sternite VIII conical with shallow notch at tip. Male sternite VIII with three emarginations (Fig. 34B). Genitalia as in Fig. 34. The length of the ventral process of the aedeagus can vary.

##### Distribution.

Canada: ON, NB, QC (Bousquet et al. 2013). USA; **IL**, **IN**, **LA**, MI, **MO**, NY, **PA**, **SC.**

#### ﻿ Lathrobium (Lathrobium) armatum

Taxon classificationAnimaliaColeopteraStaphylinidae

Say, 1830

B9805AF2-2C4D-52EE-8307-CD28045163FC

Lathrobium armatum Say, 1830: 40.
Lathrobium deceptivum Casey, 1905: 83. New synonym.
Lathrobium nigrolucens Casey, 1905: 83. New synonym.
Lathrobium procerum Casey, 1905: Bernhauer and Schubert 1912: 255.
Lathrobium subaequale Casey, 1905: 82. New synonym.

##### Type material.

Holotype not examined. It was presumably lost with much of Say’s collection (Mawdsley, 1993). ***Lectotype****Lathrobiumdeceptivum* Casey, herein designated (USNM): “N.Y. / CASEY bequest 1925 / [red] TYPE USNM 38108 / [handwritten] *deceptivum* / Lectotype *Lathrobiumdeceptivum* Casey Desg. Haberski & Caterino.” ***Lectotype****Lathrobiumsubaequale* Casey, herein designated (USNM): “N.J. / CASEY bequest 1925 / [red] TYPE USNM 38110 / [handwritten] *subaequale* / Lectotype *Lathrobiumsubaequale* Casey Desg. Haberski & Caterino.” ***Lectotype****Lathrobiumprocerum* Casey, herein designated (USNM): “Ill. / CASEY bequest 1925 / [red] TYPE USNM 38110 / [handwritten] *procerum* / Lectotype *Lathrobiomaprocerum* Casey Desg. Haberski & Caterino.” ***Lectotype****Lathrobiumnigrolucens* Casey, herein designated (USNM): “Mass/ CASEY bequest 1925 / [red] TYPE USNM 38109 / [handwritten] *nigrolucens* / Lectotype *Lathrobiomanigrolucens* Casey Desg. Haberski & Caterino.”

##### Other material.

Canada: Ontario (1, USNM). Quebec: MRC des Deux-Montagnes, Parc National d’Oka, 06 May 2023, R. Vigneault, handpicked near a beach (1, NBC). USA: Illinois (6, USNM). Indiana: Allen Co.: New Haven, 15 Jun 1986, N. M. Downie (1, UNHC). Massachusetts: ‘Mass.’ (1, MCZ). Michigan: ‘Mi.’ (2, MCZ). Missouri: ‘Mo.’ (1, MCZ). New Hampshire: Strafford Co.: Spruce Hole Conservation Area, 3 mi SW Durham, 09 Jun 1982, D. S. Chandler, bog margin in oak, beech, huckleberry litter (17, UNCH); same data, except 16 Apr 1982, marsh (1, UNHC); same data, except 22 Apr 1982, near bog edge (2, UNHC); same data, except 30 Aug 1982, along pond (5, UNHC); Hillsborough Co.: Antrim, 11 Jun 1932, C. A. Frost (1, UNHC); Rockingham Co.: Hampton, 15 Mar 2003, S. A. Shaw (1, UNHC); Rockingham Co.: Hampton, 10 Jun 2011 (1, UNHC); Grafton Co.: Rumney, 22 Apr 1926, P. J. Darlington (1, MCZ). New York (7, USNM); North Carolina (1, MCZ). Vermont: Burlington Co., Apr 1953, D. S. Chandler (1, UNHC).

##### Diagnosis.

*Lathrobiumarmatum* is similar in external appearance to *L.amplipenne* and *L.pedale* and are especially difficult to distinguish form *L.praelongum*. The female terminalia of *L.armatum* are shorter with a more convex inner margin, and its paraprocts are longer relative to the basal portion of tergite IX than in the aforementioned species. Males are easily identified by their aedeagus, which has a unique rear-facing hook projecting form the apex of the dorsal plate.

##### Description.

**Figures 35–40.**
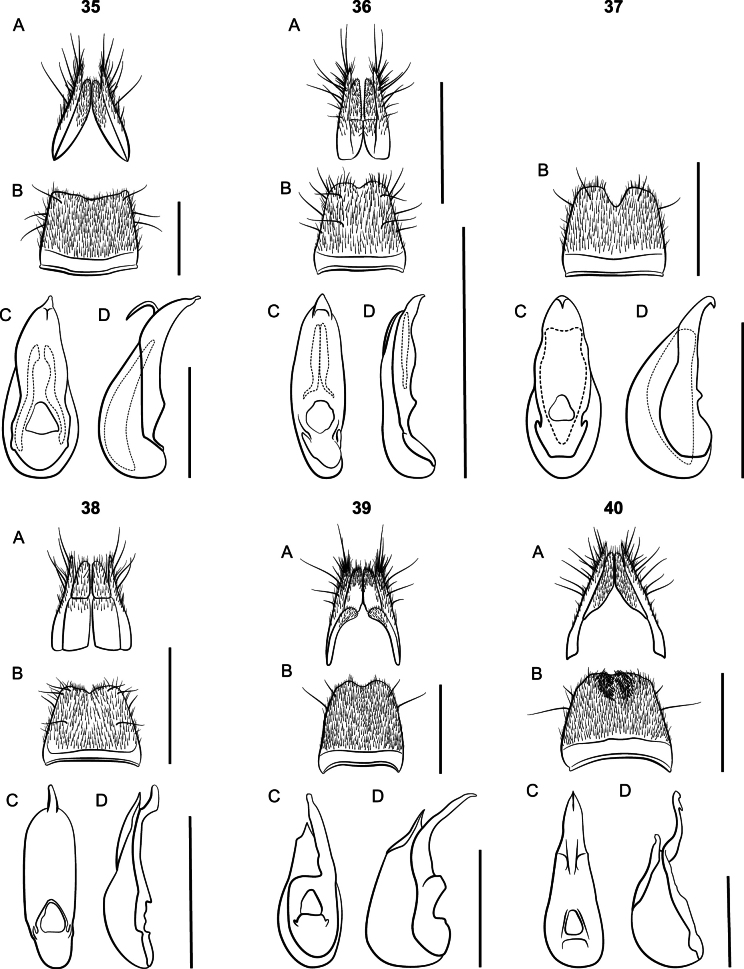
*Lathrobium* terminalia **A** female terminalia **B** male sternite VIII **C** aedeagus in ventral view **D** aedeagus in lateral view. **35***L.armatum***36***L.confusum***37***L.crurale***38***L.fauveli***39***L.fulvipenne***40***L.geminum.* Scale bars: 1 mm.

Large species, body length 10 mm; body coloration dark, appendages red, elytra sometimes bicolored. Gular sutures converging, nearly touching posteriorly; antennomeres V–VII as wide as long. Elytra 1.3× longer than pronotum. Females with paraprocts undivided, apical lobes shorter than basal portion, ~ 0.9× as long; sternite VIII conical. Male sternite VIII either with a small round emargination or no emargination. Dorsal plate of aedeagus with large, rear-facing hook (Fig. 35).

##### Distribution.

Canada: ON, NB, QC (Bousquet et al. 2013). USA: DC, IL, IN, MA, **MI**, **MO**, NH, NJ, NY, **NC**, SC, VT.

##### Remarks.

We reduce *Lathrobiumdeceptivum*, *Lathrobiumnigrolucens*, and *Lathrobiumsubaequale* to synonymy with *Lathrobiumarmatum* based on a lack of distinguishing morphological characters. Casey recognized that the herein synonymized species were difficult to discriminate and that they might not be full species. He differentiated them based on a combination of elytra length and color. *Lathrobiumsubequale* was described as having bicolored elytra, in contrast to the solid black elytra of *L.armatum*. However, this character is variable and specimens of *L.armatum* in LeConte’s collection have lightly bicolored elytra. *Lathrobiumdeceptivum* and *L.nigrolucens* were distinguished from *L.armatum* based on the length of elytra relative to pronotum, but many species are dimorphic for this character. *Lathrobiumdeceptivum* was described from two males, *L.subaequale* from a single female, and *L.nigrolucens* from a short series of both. Genitalia of all of the above species were indistinguishable.

#### ﻿ Lathrobium (Lathrobium) confusum

Taxon classificationAnimaliaColeopteraStaphylinidae

LeConte, 1880

357D121D-EC99-5CC0-BE5E-3D932B8A64D3

Lathrobium confusum LeConte, 1880: 176.
Litolathra confusa (LeConte, 1880): Casey 1905: 96.
Litolathra amputans Casey, 1905: 95. New synonym.
Lathrobium (Litolathra) amputans : Bernhauer and Schubert 1912: 255.
Litolathra convictor Casey, 1905: 95. New synonym.
Lathrobium (Litolathra) convictor : Bernhauer and Schubert 1912: 257.
Litolathra inornata Casey, 1905: 96. New synonym.
Lathrobium (Litolathra) inornatum : Bernhauer and Schubert 1912: 261.
Litolathra suspecta Casey, 1905: 97. New synonym.
Lathrobium (Litolathra) suspectum : Bernhauer and Schubert 1912: 268.

##### Type material.

***Lectotype***, *Lathrobiumconfusum* LeConte, herein designated (MCZ): White disc / “[handwritten] *L.confusum* Lec. / [handwritten] 8117 / [red] Type 6456 / Lectotype *Lathrobiumconfusum* LeConte Desg. Haberski & Caterino.” ***Lectotype***, *Litolathraamputans* Casey, herein designated (USNM): “Ia. / CASEY bequest 1925 / [red] TYPE USNM 38132 / [handwritten] *amputans* / Lectotype *Litolathraamputans* Casey Desg. Haberski & Caterino.” ***Lectotype***, *Litolathraconvictor* Casey, herein designated (USNM): “City ham / CASEY bequest 1925 / [red] TYPE USNM 38130 / [handwritten] *convictor* / Lectotype *Litolathraconvictor* Casey Desg. Haberski & Caterino.” ***Lectotype***, *Litolathrainornata* Casey, herein designated (USNM): “Wshngtn D.C. / CASEY bequest 1925 / [red] TYPE USNM 38133 / [handwritten] *inornata* / Lectotype *Litolathrainornata* Casey Desg. Haberski & Caterino.” ***Lectotype***, *Litolathrasuspecta* Casey, herein designated (USNM): “N.Y. / ♂ / CASEY bequest 1925 / [red] TYPE USNM 38131 / [handwritten] *suspecta* / Lectotype *Litolathrasuspecta* Casey Desg. Haberski & Caterino.”

##### Other material.

USA: Connecticut: New Haven Co.: New Haven (5, USNM). Massachusetts: ‘Mass’ (4, USNM). New Hampshire: Strafford Co.: Cooper Cedar Woods 1mi SE New Durham, 15 Aug 1982, D. S. Chandler, cedar swamp litter (1, UNHC); Strafford Co.: Foss Farm Rd water tower, 22 Oct 1982, W. J. Morse (1, UNHC); same data, except 30 Aug 1982 (3, UNHC); Strafford Co.: Spruce Hole Conservation Area 3 mi SW Durham, 09 Jun 1982, D. S. Chandler, litter on bog edge (12, UNHC); Strafford Co.: 1 mi SW Durham, 30 Oct 1982, W. J. Morse (4, UNHC). New Jersey: Mercer Co.: Princeton, 07 Jun 2021, A. Deczynski (1, CUAC). New York: ‘N. Y.’ (4, USNM); ‘skill NY’ [=Catskills] (1, USNM); Yates Co.: Dundee (1, USNM). North Carolina: Henderson Co.: Bearwallow Mountain, 10 Aug 2021, M. Caterino, A. Haberski, flood debris (1, CUAC); Jackson Co.: 6 km S Cashiers, 20 Jun 1986, A. Smetana (1, CNC). South Carolina: Pickens Co.: Clemson, 07 May 1940 (1, CUAC). Virginia: Shenandoah Co.: George Washing National Forest, Crooked Run Rd, Rte 720, 17 Mar 2019, C. harden, litter/duff along stream in laurel hardwoods (1, CUAC); Shenandoah Co.: George Washing National Forest, Dead Deer Creek near Signal Knob, 01 May–31 May 2015, C. Harden, flood debris (1, CUAC); Shenandoah Co.: George Washing National Forest, Rte 678 2 mi S jct w/619, 03 Jul 2018, C. Harden (1, CUAC); Shenandoah Co.: Passage Creek near Elizabeth Furnace Recreational Area, 20 Mar 2017, C. Harden, flood debris (1, CUAC); Highland Co.: Locust Springs, George Washing National Forest, 28 Jul 2017, C. Harden (3, CUAC); Smyth Co.: Mount Rogers, 15 Jun 2019, C. Harden (1, CUAC); Smyth Co.: Whitetop Mountain, 16 Jun 2019, C. Harden, litter along stream (1, CUAC); Powhatan Co.: Powhatan State Park, 02 Apr 2017, C. Harden, wetlands, nr vernal pool (1, CUAC); Washington D. C.: ‘D. C.’ (1, USNM). West Virginia: Pocahontas Co.: Cranberry Wilderness, Monongahela National Forest, 11 Jun 2019, C. Harden, under rock nr steep stream in spruce/hardwoods (1, CUAC); Pocahontas Co.: Gaudineer Knob Scenic Area, 29 Jun 2017, C. Harden (2, CUAC); Pocahontas Co.: Kennison Mountain FS Rd 232, 20 May 2018, C. Harden, litter nr rushing ephemeral rivulet (1, CUAC); Pocahontas Co.: Pocahontas Trail near Cranberry Mountain Lodge, 10 Sep 2017, C. Harden (1, CUAC).

##### Diagnosis.

*Lathrobiumconfusum* superficially resembles *Lathrobioma*, although it is not closely related. It differs from all *Lathrobioma* by having long antennomeres, and males lack the thick black setae on sternite VIII that are characteristic of the subgenus. *Lathrobiumconfusum* is more closely related to *L.rhodeanum*, with which is shares characteristically long antennomeres and paraprocts. The two can be distinguished by the hind tarsomeres which are shorter and more compact in *L.confusum*.

##### Description.

Body length 6 mm; coloration dark, appendages light yellow. Gular sutures arcuate (Fig. 1C); antennomeres V–VII longer than wide. Elytra approximately as long as pronotum. Male sternite VIII with shallow notch and no thick setae; Female with paraprocts undivided, apices 2.6× longer than basal portion, sternite VIII conical; genitalia as in Fig. 36.

##### Distribution.

Canada: ON, NB, QC (Bousquet et al. 2013). USA: **CT**, DC, IA, MA, NH, **NJ**, NY, **NC**, **SC**, **VA**, **WV**.

##### Remarks.

We reduce *Lathrobiumamputans*, *Lathrobiumconvictor*, *Lathrobiuminornatum*, and *Lathrobiumsuspectum* to synonymy with *Lathrobiumconfusum*, because the characters given by Casey (1905) were either inaccurate or insufficiently distinctive. *Lathrobiumamputans* was described as having elytra shorter than wide in females, and those of *L.confusum* were said to be subequal. Upon measuring the types, we found the elytra to be subequal in length and width in both species. *Lathrobiumconvictor* was described as having elytra equal in length to the pronotum, and those of *L.confusum* were described as shorter than the pronotum, but we found the elytra of both species were 1.1–1.2× longer than the pronotum.

*Lathrobiuminornatum* was described as having the emargination of sternite VIII smaller, shallower, and more broadly rounded compared to that of *L.confusum*, but we found no differences in width or depth. The difference in shape was modest and the emargination of *L.amputans* was actually less round than that of *L.confusum*.

*Lathrobiumsuspectum* and *L.confusum* were differentiated based on obscure minutia such as form “moderately stout” vs “rather stouter”, pronotal punctures “fine and moderately sparse” vs “moderately coarse and sparse,” but these differences were not observed in the types (Casey 1905). The length of the antennae relative to the head and pronotum combined were supposedly shorter in *L.suspectum*, but this too proved incorrect when measured.

We examined the genitalia of the above species and found no differences in aedeagi or female terminalia.

#### ﻿ Lathrobium (Lathrobium) crurale

Taxon classificationAnimaliaColeopteraStaphylinidae

(Casey, 1905)

297DC242-948B-50A0-A961-0C8BADC64EEB

Litolathra cruralis Casey, 1905: 94.
Lathrobium (Litolathra) crurale : Bernhauer and Schubert 1912: 258.

##### Type material.

***Lectotype****Litolathracruralis* Casey, herein designated (USNM): “N. J. / ♂ / CASEY bequest 1925 / [red] TYPE USNM 38129 / Lectotype *Litolathracruralis* Casey Desg. Haberski & Caterino.”

##### Other material.

USA: Iowa: ‘Ia.’ (1, USNM). New Jersey: same data as lectotype (1, USNM). Ohio: Ross Co. (1, USNM).

##### Diagnosis.

This species closely resembles *L.fauveli*. The antennomeres IV and V are 2× as long as wide in *L.crurale* and 1.6× times as long in *L.fauveli*. Males of *L.crurale* can also be distinguished by the deeper emargination on sternite VIII which is ~ ¼ the depth of the sternite, as opposed to 1/10 as deep in *L.fauveli*. The female sternite VIII is more conical in *L.fauveli* than *L.crurale*.

##### Description.

Body length 7 mm; body coloration red, appendages light yellow. Gular sutures parallel, widely separate; antennomeres V–VIII 2× as long as wide. Elytra as long as pronotum. Females with paraprocts undivided, apices longer than basal portion; sternite VIII oblong. Male genitalia as in Fig. 37. We were unable to dissect the genitalia of Casey’s female specimens.

##### Distribution.

USA: IA, NJ, OH.

#### ﻿ Lathrobium (Lathrobium) fauveli

Taxon classificationAnimaliaColeopteraStaphylinidae

Duvivier, 1883

96AE749B-BA37-55F2-B725-BC9D4F6AD709

Lathrobium fauveli Duvivier, 1883: 161.
Lathrobium simplex LeConte, 1880: 176 (preoccupied).
Lathrobium gravidulum Casey, 1905: 90. New synonym.
Lathrobium innocens Casey, 1905: 89. New synonym.
Lathrobium neglectum Casey, 1905: 89. New synonym.

##### Type material.

***Lectotype***, *Lathrobiumfauveli* Duvivier, herein designated (MCZ): Faded disc / “♂ / [handwritten] *L.simplex* Lec. / [red] Type 6451 / Lectotype *Lathrobiumsimplex* LeConte Desg. Haberski & Caterino.” ***Lectotype***, *Lathrobiumgravidulum* Casey, herein designated (USNM): “Mass / ♂ / CASEY bequest 1925 / [red] TYPE USNM 38124 / [handwritten] *gravidulum* / Lectotype *Lathrobiumgravidulum* Casey Desg. Haberski & Caterino.” ***Lectotype***, *Lathrobiuminnocens* Casey, herein designated (USNM): “Marq / ♂ / CASEY bequest 1925 / [red] TYPE USNM 38126 / [handwritten] *innocens* / Lectotype *Lathrobiuminnocens* Casey Desg. Haberski & Caterino.” ***Lectotype***, *Lathrobiumneglectum* Casey, herein designated (USNM): “RI / CASEY bequest 1925 / [red] TYPE USNM 38125 / [handwritten] *neglectum* / Lectotype *Lathrobiumneglectum* Casey Desg. Haberski & Caterino.”

##### Other material.

Canada: Nova Scotia: Queens Co.: Kejimkujik National Park, 09 May 1999, J. C. Ciegler (1, CUAC). Quebec: MRC de Portneuf: Pont-Rouge, 22 Apr 2023, N. Bédard, sifted from dead leaves in a sandpit (1, NBC). USA: Connecticut: New Haven Co. New Haven (6, USNM). Massachusetts: ‘Mass.’ (1, MCZ); ‘Mass.’ (4, USNM). Michigan: Marquette Co.: Marquette (1, USNM). New Hampshire: Grafton Co.: Downes Brook, Potash Mountain, 05 Jun 2021, A. Haberski, along creek edge (1, CUAC); Coos Co.: Mount Washington 2700’ elev. (1, UNHC); Strafford Co.: Spruce Hole Conservation Area, D. S. Chandler (1, UNHC); Grafton Co.: Hubbard Brook Experimental Forest, D. S. Chandler (1, UNHC). Rhode Island: ‘R. I.’ (1, USNM).

##### Diagnosis.

This species closely resembles *L.crurale* and can be distinguished from that species by the characters discussed in the diagnosis of *L.crurale*.

##### Description.

Body length 8 mm; body coloration dark red, appendages lighter red. Gular sutures parallel, widely separate; antennomeres V–VIII longer than wide. Elytra as long as pronotum. Females with paraprocts undivided, apices longer than basal portion; sternite VIII conical. Ventral process of aedeagus with distinctive apical tooth (Fig. 38).

##### Distribution.

Canada: AB, BC, MB, NB, NF, NS, ON, PE, QC, SK (Bousquet et al. 2013). USA: CT, MA, ME, MI, NH, RI, WI (Casey 1905; Newton 2022).

##### Remarks.

We reduce *Lathrobiumgravidulum*, *Lathrobiuminnocens*, and *Lathrobiumneglectum* to synonymy with *Lathrobiumfauveli*, based on the absence of clear distinguishing characters. *Lathrobiumneglectum* and *L.innocens* were distinguished from *L.fauveli* based on larger emarginations in male sternite VIII. However, we found no difference in size among species. *Lathrobiumgravidulum* was distinguished based on subtle differences in punctation and the coloration of appendages, but neither are consistent. Examination of the genitalia found no differences in either aedeagi or female terminalia.

#### ﻿ Lathrobium (Lathrobium) fulvipenne

Taxon classificationAnimaliaColeopteraStaphylinidae

(Gravenhorst, 1806)

97435346-6046-5BAD-B4D7-476F321C6E44

Lathrobium alpestre Heer, 1839: 239.
Lathrobium atriceps Stephens, 1833: 267.
Lathrobium castaneipenne Kolenati, 1846: 22.
Lathrobium letzneri Gerhardt, 1869: 257.
Lathrobium muelleri Bernhauer, 1899: 435.
Lathrobium punctulatum Mannerheim, 1830: 37.
Staphylinus fulvipenne Gravenhorst, 1806: 104.
Staphylinus fulvipennis Gravenhorst, 1806: 104.

##### Type material.

Types not examined.

##### Diagnosis.

Males have a distinctive, asymmetrical aedeagus that differs from all other Nearctic species (Fig. 39C). Females can be distinguished by the shape of their valvifers (Fig. 39A).

##### Description.

Body length 8 mm; body coloration dark, appendages and mouthparts light red, elytra bicolored with narrow black base, or monochromatic black. Gular sutures parallel; antennomeres V–VII longer than wide. Wing dimorphic, elytra as long or slightly longer than pronotum. Females tergite IX with paraprocts undivided, apical lobes shorter than basal portion in dorsal view; sternite VIII oblong, valvifers and coxites divided (Fig. 39A). Male sternite VIII without patches of dark setae, apical emargination shallow and round (Fig. 39 B). Ventral process of aedeagus asymmetrical in ventral view (Fig. 39C; Assing and Schülke 2012).

##### Distribution.

Canada: AB, BC, NB, NF, QC (Bousquet et al. 2013).

##### Remarks.

Native to the Palearctic and adventive in North America. Common in unforested habitats (Assing and Schülke 2012).

#### ﻿ Lathrobium (Lathrobium) geminum

Taxon classificationAnimaliaColeopteraStaphylinidae

Kraatz, 1857

BEDEBC7C-21C6-5096-8E47-9A3EEB6C5101

Lathrobium bicolor Heer, 1839: 240 (junior homonym).
Lathrobium boreale Thomson, 1860: 198.
Lathrobium boreale Hochhuth, 1851: 41.
Lathrobium difficile Coiffait, 1953: 104.
Lathrobium fallaciosum Coiffait, 1953: 104.
Lathrobium obscuriceps Motschulsky, 1860: 564.
Lathrobium rufescens Motschulsky, 1860: 563.
Lathrobium volgense Hochhuth, 1851: 42.

##### Type material.

Types not examined.

##### Diagnosis.

In North America, this species is most similar to *L.amplipenne*. Males can be distinguished from *L.amplipenne*, and all other Nearctic species, by their distinctive aedeagus (Fig. 40D), and females can be distinguished by their short gonocoxites, which are only ~ ½ as long as their paraprocts, rather than subequal as in *L.amplipenne*.

##### Description.

Large species, body length 8–11 mm; body coloration dark, appendages light brown, elytra bicolored with broad black base, rarely solid black. Gular converging, antennomeres V–VII 1.2× as long as wide. Wing dimorphic, elytra approximately as long as pronotum. Females tergite IX with paraprocts undivided, apical lobes shorter than basal portion in dorsal view; sternite VIII with truncate apex; valvifers and coxites fused (Fig. 40A). Male sternite VIII with two longitudinal patches of dark setae in posterior third, apex indistinctly emarginated. Ventral process of aedeagus distinctively shaped with apical tooth (Fig. 40D) (Assing and Schülke 2012).

##### Distribution.

Canada: BC (Pentinsaari et al. 2019).

##### Remarks.

Native to the Palearctic and adventive in North America. Common in moist, open habitats (Assing and Schülke 2012). Canadian specimens collected in wetland adjacent to lake (Pentinsaari et al. 2019).

#### ﻿ Lathrobium (Lathrobium) islae

Taxon classificationAnimaliaColeopteraStaphylinidae

Haberski & Caterino sp. nov.

259799AF-ECA8-5594-AFAF-E0CFE7C68725

https://zoobank.org/F12B5DD3-06F5-4F5B-AE25-1BF7B7B593B0

##### Type material.

***Holotype*** ♂ (FMNH): “USA: NC: Caldwell Co., 36.1117°N, 81.8068°W, Grandfather Mountain, Calloway Peak X.6.2020, M. Caterino, F. Etzler, A. Haberski, sifted litter.” / “Caterino DNA voucher, Ext. MSC-6239” / “CLEMSON ENT [QR CODE] CUAC000169030”. ***Paratypes* (36, CUAC, FMNH, VMNH)**: 10: same locality as type, 36.1118°N, 81.8105°W, x.06.2020 (CUAC000112914, CUAC000112915, CUAC000177130, CUAC000177131, CUAC000177132, CUAC000177133, CUAC000177134, CUAC000177135, CUAC000177136, CUAC000177137); 3: same locality as type, 36.0978°N, 81.8293°W, 5370ft., iv.21.2022 (CUAC000177093, CUAC000177094, CUAC000177095); 1: same locality as type, 36.1118°N, 81.8112°W, x.06.2020 (CUAC000177109); 20: same locality as type, 36.1117°N, 81.8088°W, x.06.2020 (CUAC000177110, CUAC000177111, CUAC000177112, CUAC000177113, CUAC000177114, CUAC000177115, CUAC000177116, CUAC000177117, CUAC000177118, CUAC000177119, CUAC000177120, CUAC000177121, CUAC000177122, CUAC000177123, CUAC000177124, CUAC000177125, CUAC000177126, CUAC000177127, CUAC000177128, CUAC000177129); 1: same locality as type, 36.1104°N, 81.8046°W, v.17.2021 (CUAC000177138); 1: same locality as type, 36.1116°N, 81.8117°W, V.17.2021 (CUAC000135036).

##### Other material.

North Carolina: Mitchell Co.: Grassy Ridge Bald, 6135’, (36.0985, -82.1791), M.S. Caterino, 08 Jun 2020 (CUAC); Mitchell Co.: Roan High Bluff, 6225–6251’, (36.0931, -82.1453), M.S. Caterino, 15 Aug 2018 (3, CUAC); Mitchell Co.: Roan High Knob, 5756–6286’, (36.1045, -82.1224), M.S. Caterino, 08 Jun 2020 (3, CUAC). Virginia: Smyth Co.: Mt. Rogers, 5699’, Jefferson National Forest (36.6602, -81.5447), M.S. Caterino & P. Marek, 03 Jul 2018 (7, CUAC); Smyth Co.: Mt. Rogers, 5699’, Jefferson National Forest (36.6602, -81.5447), M.S. Caterino & P. Marek, 03 Jul 2018, CUAC000187893, CUAC000187899 (15 larvae, CUAC); Smyth Co.: Mt. Rogers, 5666–5680’, Jefferson National Forest (36.6605, -81.5447), M.S. Caterino & F. Etzler, A. Haberski, 27 Oct 2020 (27, CUAC); Smyth Co.: Mt. Rogers, 5686’, Jefferson National Forest (36.6612, -81.5456), M.S. Caterino, 27 Oct 2020 (3, CUAC); Smyth Co.: Mt. Rogers, Jefferson National Forest (36.657, -81.555), C.W. Harden, 15 Apr 2019 (CUAC); Smyth Co.: Whitetop Mountain, 5503’, Jefferson National Forest (36.6391, -81.6064), M.S. Caterino & P. Marek, 03 Jul 2018 (CUAC); Smyth Co.: Whitetop Mountain, 5436’, Jefferson National Forest (36.6379, -81.6053), M.S. Caterino, 27 Oct 2020 (CUAC); Smyth Co.: Whitetop Mountain, Jefferson National Forest (36.629, -81.596), C.W. Harden, 15 Apr 2019 (CUAC);Grayson Co.: Wilburn Ridge, 5450’, Jefferson National Forest (36.6525, -81.5167), M.S. Caterino & P. Marek, 03 Jul 2018 (2, CUAC).

##### Diagnosis.

This species can be distinguished from the closely related *L.lividum* only by its genitalia. The spines of the internal sac of their aedeagi differ conspicuously (Fig. 7E, F vs Fig. 8D, E). Differences in female genitalia are more subtle, but the gonocoxites of *L.islae* are narrower at their base. No intermediate forms are known.

Four other species of Nearctic *Lathrobium* have short elytra and functional eyes, but none are likely to be mistaken for *L.islae* or *L.lividum*. *Lathrobiumbrevipenne*, *L.carolinae*, and *L.camplyacra* are twice as large, lighter in color, and have an overall different gestalt. *Lathrobiumpallescens* has a pale red body color and its eyes are much smaller, 1/8 the lateral length of the head with ~ 30 ommatidia.

##### Description.

Habitus (Fig. 7A). Small species, total body length ~ 4.5 mm long, FL 2.5–3.0 mm long. Coloration: body black; legs, palpomeres, and antennae dark red.

Head subquadrate, as wide as long, widest posteriorly and narrower anterior to eyes; posterior angles slightly rounded; epicranium coarsely punctate, punctures less dense in median dorsal and anterior regions; interstices with strong transversely reticulate microsculpture throughout; head setose throughout, with long macrosetae projecting at posterior corners of head, corners of the eyes, laterally posterior to the eyes, and above the mandible insertions; gular sutures arcuate, widely separate, 1/16 width of head apart at their most proximal point; neck ½ as wide as head. Eyes large and well developed with ~ 95 ommatidia, occupying ¼ lateral length of the head. Antennae moniliform, as long as head and pronotum combined; scape as long as antennomeres II and III combined; antennomeres II–IV elongate, gradually widening so that antennomeres V–IX are as wide as long; apical antennomere longer, subacute.

Pronotum longer than wide, as wide or slightly wider than head; sides parallel; angles rounded; punctures large, spaced one diameter apart, impunctate at midline with a visible line; interstices shiny with a finely punctate microsculpture. Elytra shorter but slightly wider than pronotum, as wide as head, as long as wide; anterior angles somewhat squared, posterior margins sinuate; scutellum round; punctures small, irregularly spaced, most 1–2× their diameter apart; interstices with finely punctate microsculpture. Hindwings vestigial, 0.2 mm long, 1/5 length of elytra. Posterior margin of abdominal tergite VII without palisade fringe.

♂: Sternite VII flattened medially with shallow notch on the posterior margin; posterior margin of sternite VIII with a deep U-shaped notch (Fig. 7D). Aedeagus 1.7 mm long (Fig. 7E, F), ventral process short, not extending much beyond the median foramen posteriorly, lightly sclerotized portion of the median lobe protruding beyond it in lateral view; dorsal plate asymmetrical; internal sac with a single large spine with a characteristic club tip in ventral view.

♀: Sternite VIII slightly oblong with a triangular patch of dense setae at the tip (Fig. 7C); paraprocts divided anteriorly; proctiger conical. sternum IX with valvifers and coxites fused, valvifers narrow at base (Fig. 7B); subgenital plate absent.

**Figure 41.**
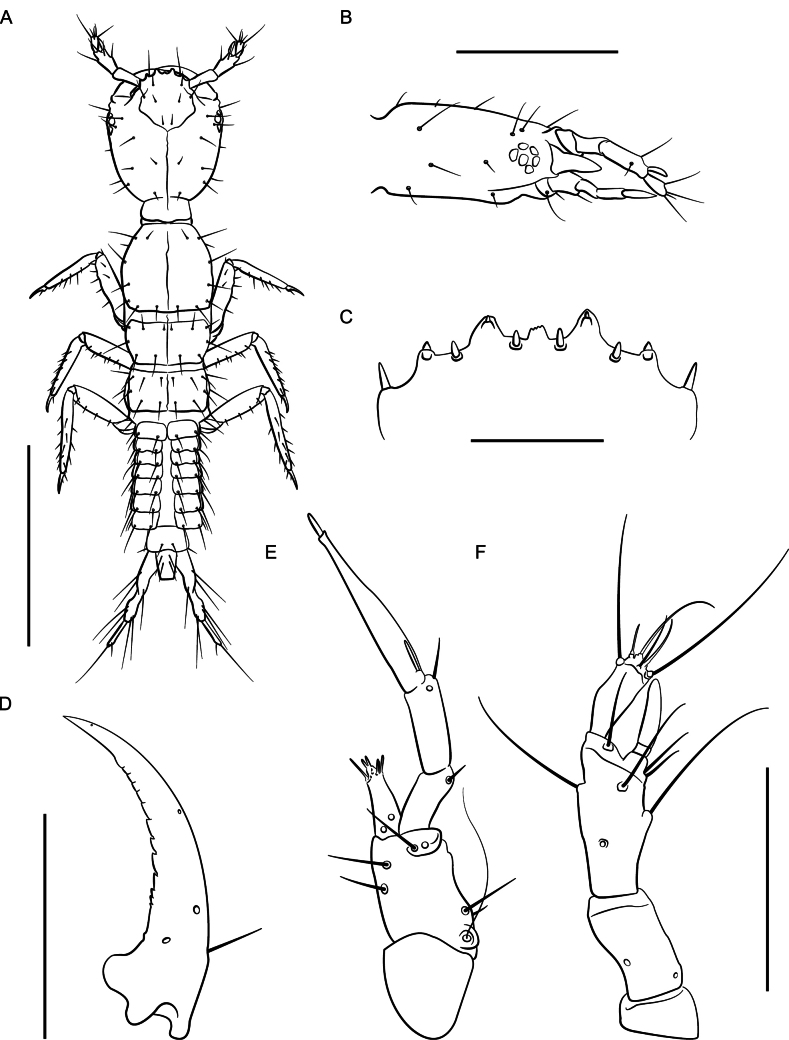
*Lathrobiumislae* first instar larva **A** habitus **B** head in lateral view **C** nasale **D** mandible **E** maxilla **F** antenna. Scale bars: 1 mm (**A**); 500 μm (**B**); 100 μm (**B, D**); 250 μm (**E, F**).

***First instar larva***: Body elongate, ~ 3 mm long; well sclerotized (Fig. 41A); head, thoracic, and abdominal tergites brown, appendages light yellow, intersegmental membrane white, translucent.

Head ovate, widest at stemmata and slightly tapered posteriad (in dorsal view), dorsoventrally flattened, 1.1× as long as wide, dorsal setae as in Fig. 41A; head 2.5× wider than neck, dorsal ecdysial lines bifurcate 1/2 distance between neck and nasale margin; six stemmata present, arranged as in Fig. 41B; anterior margin of nasale (Fig. 41C) with nine blunt teeth pointing anteriorly, one quadrate median tooth with tetradentate anterior edge, a pair of paramedian teeth, and three pairs of lateral teeth; the innermost lateral teeth are small and indistinct; paramedian and lateral teeth armed with nodular setae, and a pair of nodular setae separate median and paramedian teeth; Apotome of gula not reaching tentoral pits.

Antennae (Fig. 41F) length ratio: 1.0:2:3.1:1.6; antennomere I triangular; antennomere II with two pores; antennomere III with three elongate macrosetae, three solenidia, one pore; antennomere IV club-shaped with apical solenidia; sensory appendage 0.9× as long as antennomere IV.

Mandibles (Fig. 41D) long, falciform, serrate along inner margin, with a single seta near the base on the outer ventral edge. Maxilla (Fig. 41D) with cardo triangular, widening from base to apex; stipes quadrate, 1.3× longer than cardo; mala digitiform, tapering toward apex, 0.9× as long as palpomere I, with apical sensory appendages and two pores; palpifer with one seta. Maxillary palpomere length ratios: 1.0:1.2:2.8; palpomere II with two setae; palpomere III with one basal sensory appendage and numerous small apical appendages. Labium with prementum subquadrate, basal portion strongly sclerotized; ligula with elongate membranous apex, twice as long as wide, densely fimbriate, separated from prementum by a distinctly sclerotized transverse strip; palpomere I 1.6× as long as II; palpomere II bearing short sensilla at apex.

Dorsal sclerites of thorax with ecdysial lines along midline of body; prothorax subquadrate, narrowing slightly anteriorly, chaetotaxy as in Fig. 41A; thoracic tergite II and III subequal; abdominal sclerites well sclerotized, with two small pleural sclerites per segment on each side; basal segment of urogomphus 2.6× as long as terminal segment, with seven prominent lateral setae; terminal segment of urogomphus slender, with one short and one long apical seta.

**Figure 42.**
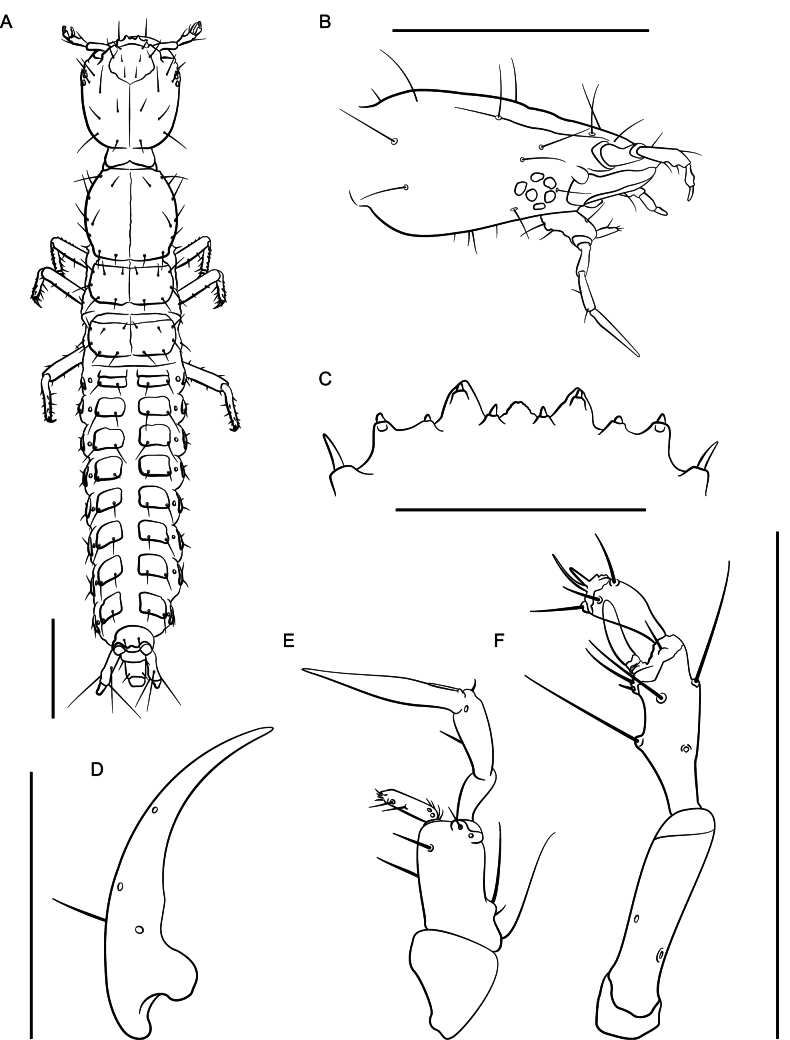
*Lathrobiumislae* second instar larva **A** habitus **B** head in lateral view **C** nasale **D** mandible **E** maxilla **F** antenna. Scale bars: 1 mm (**A, B**); 250 μm (**C**); 500 μm (**D, E, F**).

***Second instar larva***: Second instar (Fig. 42A) resembles first, except as follows. Body larger, ~ 6 mm long. Head subquadrate; dorsal ecdysial lines of the head bifurcate 2/5 distance between nasale margin and neck; median tooth of nasale triangular with serrated edges and a blunt tip (Fig. 42C). Apotome of gula reaching tentoral pits. Antenna (Fig. 42F) length ratios: 1.0:3.5:2.8:1.5; antennomere IV club-shaped with apex 2× as wide as base. Mandible interior margin smooth (Fig. 42D). Maxilla palpomere length ratios: 1.0:1.5:3.3; Labial palpomere I 1.5× II (Fig. 42E); palpomere I distinctly curved; palpomere II bent near apex. Prothorax round. Urogomphi broken off of only known specimen.

**Figure 43.**
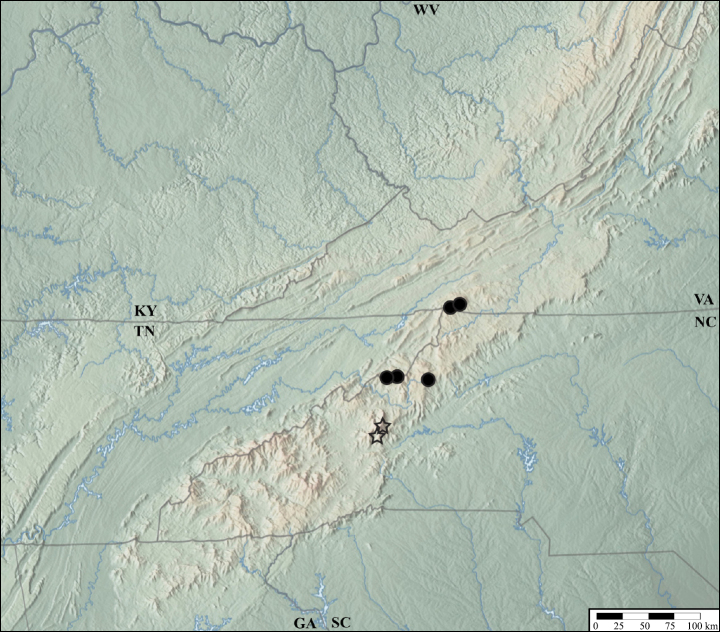
Distribution of *Lathrobiumislae* (circle), *L.lividum* (star).

##### Etymology.

Named in honor of Isla Haberski, daughter of the first author, who was born during the preparation of this manuscript.

##### Distribution and ecology.

*Lathrobiumislae* inhabits spruce-fir forests above 1500 m in the Grayson Highlands, Roan Highlands, and on Grandfather Mountain (Fig. 43). It can be collected from leaf litter but is most common on boulders beneath bryophyte mats. It has not been found in spruce-fir forests south of the French Broad River basin, where its microhabitat is inhabited by *L.smokiense* and *L.balsamense*. Adults collected Jul–Sep. Larvae collected Mar–Jul.

##### Remarks.

*Lathrobiumislae* was not monophyletic in our COI phylogeny and ASAP identified five genetic partitions. Given this degree of genetic variation, populations outside the type locality might also differ in minor ways morphologically, perhaps in characters not yet evaluated. Larvae were associated with adults by DNA barcoding.

#### ﻿ Lathrobium (Lathrobium) leconteanum

Taxon classificationAnimaliaColeopteraStaphylinidae

Scheerpeltz, 1933

F9D9F355-F1B4-5382-BABC-17D73B0BA094

Lathrobium leconteanum Scheerpeltz, 1933: 1274.
Lathrobium concolor LeConte, 1880: 175 (junior homonym).

##### Type material.

***Lectotype***, *Lathrobiumleconteanum* Scheerpeltz, herein designated (MCZ): Faded disc / “♂ ♀ / [handwritten] *L.concolor* Lec. / [red] Type 6450 / Lectotype *Lathrobiumconcolor* LeConte Desg. Haberski & Caterino.”

##### Other material.

USA: Indiana: ‘Ind.’ (1, USNM). New Hampshire: Coos Co.: Mount Washington (1, UNHC); Grafton Co.: Hubbard Brook Experimental Forest, D. S. Chandler (1, UNHC).

##### Diagnosis.

This species is similar in appearance to *L.washingtoni* but differs in having elongate antennomeres. Males are otherwise difficult to tell apart, even in the primary and secondary sexual characters. *Lathrobiumleconteanum* has four spines on the internal sac of the aedeagus whereas *L.washingtoni* has two. Females are more easily differentiated because *L.leconteanum* has an undivided tergite IX and *L.washingtoni* has tergite IX fully divided.

##### Description.

**Figures 44–49.**
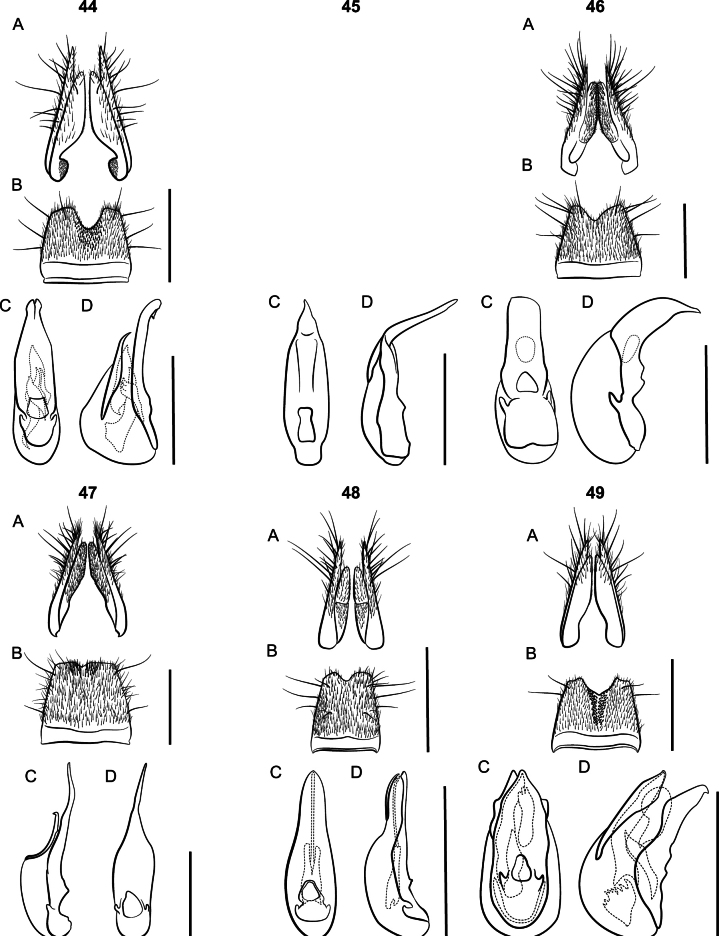
*Lathrobium* terminalia **A** female terminalia **B** male sternite VIII **C** aedeagus in ventral view **D** aedeagus in lateral view. **44***L.leconteanum***45***L.lineatocolle***46***L.pedale***47***L.praelongum***48***L.rhodeanum***49***L.simile*. Scale bars: 1 mm.

Body length 7 mm; body coloration dark red, appendages lighter. Gular sutures parallel, widely separate; antennomeres V–VII longer than wide. Elytra 1.2× longer than pronotum. Females with paraprocts undivided, apices 1.3× as long as basal portion; sternite VIII conical with small apical notch. Genitalia as in Fig. 44.

##### Distribution.

Canada: ON, NF (Bousquet et al. 2013). USA: IN, NH.

#### ﻿ Lathrobium (Lathrobium) lineatocolle

Taxon classificationAnimaliaColeopteraStaphylinidae

Scriba, 1859

535B49AF-7B99-5DB2-B972-4570ED4D53D4

##### Type material.

Types not examined.

##### Diagnosis.

This species is most similar to *L.fulvipenne* but can be distinguished by the ventral process of the aedeagus, which lacks an apical tooth and is nearly symmetrical in ventral view (Fig. 45C).

##### Description.

Body length 8 mm; body coloration dark, appendages light brown, elytra usually bicolored with narrow black base. Gular sutures parallel; antennomeres V–VII longer than wide. Elytra as long as pronotum or slightly shorter. Female tergite IX with apical lobes of paraprocts longer than continuous anterior portion in dorsal view; sternite VIII with truncate apex. Ventral process of the aedeagus strongly deflexed, distal tip lying beyond median foreman in lateral view (Fig. 45C).

##### Distribution.

Canada: ON (Pentinsaari et al. 2019).

##### Remarks.

Native to the Palearctic and adventive in North American. Found in forest and riparian habitats (Pentinsaari et al. 2019).

#### ﻿ Lathrobium (Lathrobium) lividum

Taxon classificationAnimaliaColeopteraStaphylinidae

Haberski & Caterino sp. nov.

DFAF18CF-BED1-57F9-9394-BA3B397FA1DE

https://zoobank.org/53298BFA-CDA9-49A4-9584-C2D01C39C768

##### Type material.

***Holotype*** ♂ (FMNH): “USA: NC: Yancey Co., 35.7643°N, 82.2629°W, Mt. Mitchell SP, Mt. Mitchell, 6556’, ix.07.2021, M. Caterino & E. Recuero, sifted litter.” / “Caterino DNA voucher, Ext. MSC-7880, Morphosp. MM.B.318” / “CLEMSON ENT [QR CODE] CUAC000135757”. ***Paratypes* (40)**: 5 (CUAC): same locality as type, 35.7643°N, 82.2633°W, X.06.2020 (CUAC000169022, CUAC000169023, CUAC000177098, CUAC000177099, CUAC000177100); 9 (CUAC): same locality as type, 35.7643°N, 82.2633°W, 6589ft, ix.07.2021 (CUAC000169022, CUAC000169023, CUAC000177169, CUAC000177170, CUAC000177171, CUAC000177172, CUAC000177173, CUAC000177174, CUAC000177175); 1 (CUAC): same locality as type, 35.7644°N, 82.2641°W, V.15.2018 (CUAC00079253); 5 (MCZ): “Black Mts. N. C., Mt. Mitchell 5000–6711 ft, IX.05.1930, Darlington.”; 2 (CUAC): “USA: NC: Yancey Co., 35.7798°N, 82.2599°W, Mount Mitchell State Park, Big Tom, 6586’, v.15.2018, M. Caterino, sifted litter.” (CUAC000048570); 10 (CUAC) same locality, 35.7795°N, 82.2596°W, 6554’, ix.07.2021, M. Caterino (CUAC000157555, CUAC000157567, CUAC000157568, CUAC000172501, CUAC000172505, CUAC000172506, CUAC000172509, CUAC000172511, CUAC000172513, CUAC000172514); 2 (CUAC) “USA: NC: Yancey Co., 35.8525°N, 82.2468°W, Pisgah National Forest, Celo Knob, 6284’, vi.15.2020, M. Caterino, sifted litter” (CUAC000004036, CUAC000169024); 4: “USA: NC: Yancey Co., 35.8524°N, 82.2485°W, Pisgah National Forest, Celo Knob, 6300’, x.19.2021, M. Caterino, E. Recuero & A. Haberski, sifted litter” (CUAC000177101, CUAC000177102, CUAC000177103, CUAC000177104); 4: “USA: NC: Yancey Co., 35.8527°N, 82.2487°W, Pisgah National Forest, Celo Knob, 6294’, x.19.2021, M. Caterino, E. Recuero & A. Haberski, sifted litter” (CUAC000177105, CUAC000177106, CUAC000177107, CUAC000177108); 3: “USA: NC: Yancey Co., 35.8522°N, 82.2485°W, Pisgah National Forest, Celo Knob, 6300’, vi.15.2020, M. Caterino & F. Etzler, sifted litter” (CUAC000177145, CUAC000177146, CUAC000177147); 2: “USA: NC: Yancey Co., 35.8523°N, 82.2486°W, Pisgah National Forest, Celo Knob, 6300’, vi.15.2020, M. Caterino & F. Etzler, sifted litter” (CUAC000177148, CUAC000177149); 2 (CUAC) “USA: NC: Yancey Co., 35.8525°N, 82.2468°W, Pisgah National Forest, Celo Knob, 6284’, vi.15.2020, M. Caterino, sifted litter” (CUAC000004036, CUAC000169024); 2 (CUAC) “USA: NC: Yancey Co., 35.7782°N, 82.2610°W, Mount Mitchell State Park, Mt. Craig, 6550’ v.15.2018, M. Caterino, sifted litter” (CUAC000003088, CUAC000169028).

##### Other material.

**North Carolina**: Buncombe Co.: Base of Mt. Mitchell, 5413’, Blue Ridge Parkway, A. Smetana, 03 Jun 1986 (5, CNC); Yancey Co.: Mt. Mitchell, 6561–6679’, Mount Mitchell State Park, A. Smetana, 04 Jun 1986 (6, CNC); Buncombe Co.: Craggy Dome, 5696–5845’ 13 Sep 2022 (4, CUAC).

##### Diagnosis.

This species can be distinguished from the closely related *L.islae* only by its genitalia. The spines of the internal sac of their aedeagi differ conspicuously (Fig. 8D, E vs Fig. 7E, F), but differences in female genitalia are more subtle. The gonocoxites of *L.islae* are narrowed at the base, but not in *L.lividum*. No intermediate forms are known.

##### Description.

External morphology is identical to that of *L.islae*. It differs only in genitalia.

♂: Aedeagus (Fig. 8D, E) with ventral process longer, nearly reaching the end of the median lobe; dorsal plate small and blade-like; the internal sac with a single large, curved spine that projects above the median lobe.

♀: Gonocoxite width subequal from base to apex (Fig. 8A).

##### Etymology.

The specific name is Latin, meaning bruised, in reference to its dark coloration.

##### Distribution and ecology.

*Lathrobiumlividum* might have the smallest range of any Nearctic *Lathrobium*. It is endemic to spruce-fir forests above 1500 m elevation in the Black Mountains and Craggy Mountains of North Carolina (Fig. 43). Collected Jul–Sep.

#### ﻿ Lathrobium (Lathrobium) pedale

Taxon classificationAnimaliaColeopteraStaphylinidae

LeConte, 1863

5BA0DAC6-AF33-587B-957A-D015AFCF9333

Lathrobium pedale LeConte, 1863: 43.

##### Type material.

***Lectotype***, *Lathrobiumpedale* LeConte, herein designated (MCZ): “La. / ♂ / [handwritten] *L.pedale* Lec. / [red] Type 6454 / Lectotype *Lathrobiumpedale* LeConte Desg. Haberski & Caterino.”

##### Other material.

USA: North Carolina: Swain Co.: Hazel Creek, Great Smoky Mountains National Park, 18 Jul 2003, S. L. Staines, C. Ware (1, LSAM). South Carolina: Colleton Co.: Canadys, 30 Jul 1993, J. C. Ciegler (1, CUAC); Kershaw Co.: Wateree Floodlands Memorial Forest, 13 Apr, 2021, C. Harden, pine stump/flood debris (1, CUAC). Virginia: Botetourt Co.: Solitude Swamp, 28 Jun 2018, C. Harden, deep oak litter on swamp margin (1, CUAC).

##### Diagnosis.

This species closely resembles *L.amplipenne*, *L.armatum*, and *L.praelongum* in external morphology, but differs in the primary and secondary sexual characters. Males of *L.pedale* have a large emargination on sternite VIII, where the aforementioned species have small emarginations or none at all. They also lack a dorsal plate of the aedeagus. Females differ from the three aforementioned species by having valvifers and coxites fully divided.

##### Description.

Large species, body length 9 mm; body coloration dark red, appendages lighter red or yellow. Gular sutures converging, nearly touching posteriorly; antennomeres V–VII as wide as long. Elytra as long as pronotum. Females with paraprocts undivided, apices shorter than basal portion; sternite VIII weakly oblong. Genitalia as in Fig. 46.

##### Distribution.

USA: LA, **NC**, **SC**, **VA**.

#### ﻿ Lathrobium (Lathrobium) praelongum

Taxon classificationAnimaliaColeopteraStaphylinidae

Casey, 1905

771BA83E-C72A-54D6-9C43-3893ED7E2BE8

Lathrobium praelongum Casey, 1905: 82.

##### Type material.

***Lectotype***, *Lathrobiumpraelongum* Casey, herein designated (USNM): “Coll J B: / CASEY bequest 1925 / [handwritten] *praelongum* / [red] TYPE USNM 38113 / Lectotype *Lathrobiumpraelongum* Casey Desg. Haberski & Caterino.”

##### Other material.

USA: Maryland: Allegany Co.: Little Orleans, 05 Jun 2021, A. Deczynski (1, CUAC). North Carolina: Swain Co.: Hazel Creek Great Smoky Mountains National Park, 18 Jul 2003, S. L. Staines, C. Ware (2, LSAM). Virginia: Highland Co.: Bullpasture River, 02 Jun 2019, C. Harden, streamside gravel/cobble/sand (1, CUAC); Shenandoah Co.: Passage Creek near Buzzard Rock, 18 Apr 2016, C. Harden, under stones, wet sandy soil (1, CUAC); Shenandoah Co.: Elizabeth Furnace Recreational Area, Rte 619 Mudhole Gap trail, 09 Jan 2016, C. Harden (1, CUAC); Shenandoah Co.: George Washing National Forest, Signal Knob trailhead, 25 Oct 2017, C. Harden, moist ditch in floodplain (1, CUAC); Appomattox Co.: Holiday Lake State Park, 15 Jun 2017, C. Harden (1, CUAC); Bath Co.: Whites Cave, 15 Oct 2020, C. Harden (1, CUAC).

##### Diagnosis.

This species closely resembles *L.amplipenne*, *L.armatum*, and *L.pedale* in external morphology. Males are readily distinguished by the uniquely shaped ventral process of the aedeagus that is twice as long as the median lobe. Females are more challenging, but they have shorter paraprocts than the aforementioned species, and denser pubescence on their gonocoxites.

##### Description.

Large species, body length 9 mm; body coloration dark, appendages paler red, elytra bicolored. Gular sutures converging, nearly touching posteriorly; antennomeres V–VII as wide as long. Elytra at least as long as pronotum. Females with paraprocts undivided, apices shorter than basal portion, ~ 0.7× as long; sternite VIII weakly oblong. Characteristic aedeagus (Fig. 47).

##### Distribution.

USA: **IL, MD**, NJ, **NC**, **VA**.

#### ﻿ Lathrobium (Lathrobium) rhodeanum

Taxon classificationAnimaliaColeopteraStaphylinidae

(Casey, 1905)

57EB84F6-A205-54E4-974D-2A805ABC4C17

Litolathra rhodeana Casey, 1905: 94.
Lathrobium (Litolathra) rhodeanum : Bernhauer and Schubert 1912: 266.
Litolathra semirubida Casey, 1905: 94. New synonym.
Lathrobium (Litolathra) semirubidum : Bernhauer and Schubert 1912: 267.

##### Type material.

***Lectotype***, *Litolathrarhodeana* Casey, herein designated (USNM): “R. I. / ♂ / CASEY bequest 1925 / [red] TYPE USNM 38127 / [handwritten] *rhodeana* / Lectotype *Litolathrarhodeana* Casey Desg. Haberski & Caterino.” ***Lectotype****Litolathrasemirubida* Casey, herein designated (USNM): “R.I. / CASEY bequest 1925 / [red] TYPE USNM 38128 / [handwritten] *semirubida* / Lectotype *Litolathrasemirubida* Casey Desg. Haberski & Caterino.”

##### Other material.

USA: Massachusetts: ‘Mass’ (1, USNM). New York: ‘N. Y.’ (1, USNM). Rhode Island: ‘R. I.’ (2, USNM). South Carolina: Hampton Co.: Bluff Lake, Webb Wildlife Management Area, 29 Apr 2017, J. C. Ciegler, UV light (1, CUAC). Virginia: Sussex Co.: Chub Sandhill Natural Area Preserve, 24 Sep 2017, C. Harden, litter from dark moist woods near pool (2, CUAC); Botetourt Co.: Solitude Swamp, 28 Jun 2018, C. Harden, deep oak litter on swamp margin, exposed mounds (5, CUAC).

##### Diagnosis.

This species closely resembles *L.confusum*. They differ in the lengths of the hind tarsomeres which are longer in *L.rhodeanum* than in *L.confusum*. Females are otherwise difficult to tell apart, but males are easily identified by the elongate spine in the internal sac of the aedeagus, which is unique.

##### Description.

Body length 7 mm. Body coloration red throughout. Females with paraprocts undivided, apices 2.7× longer than basal portion, sternite VIII oblong. Elytra longer and wider than pronotum. Antennomeres V–VII 2× as long as wide. Aedeagus with characteristic elongate structures of the internal sac (Fig. 48).

##### Distribution.

Canada: QC (Newton 2022). USA: MA, NY, RI, **SC**, **VA**.

##### Remarks.

We reduce *Lathrobiumsemirubidum* to synonymy with *Lathrobiumrhodeanum*. *Lathrobiumsemirubidum* was described from a single specimen that subtly differs from *L.rhodeanum* in body coloration, punctation, and head width, but these differences do not exceed the intraspecific variation observed in longer series of other *Lathrobium*. The lectotypes of both species were collected from the same location, on the same day, and their aedeagi are indistinguishable.

#### ﻿ Lathrobium (Lathrobium) simile

Taxon classificationAnimaliaColeopteraStaphylinidae

LeConte, 1863

25E53830-CC0E-59A6-85E1-1D3F72FBA9C2

Lathrobium simile LeConte, 1863: 43.

##### Type material.

***Lectotype***, *Lathrobiumsimile* LeConte, herein designated (MCZ): Pink disc / “♂ / [handwritten] *L.simile* Lec. / [red] Type 6449 / Lectotype *Lathrobiumsimile* Leconte Desg. Haberski & Caterino.”

##### Other material.

Canada: Manitoba: Aweme (4, USNM). USA: Massachusetts: ‘Mass’ (4, USNM). New Hampshire: Strafford Co.: Spruce Hole Conservation Area, D. S. Chandler (1, UNHC); Grafton Co.: Hubbard Brook Experimental Forest, D. S. Chandler (1, UNH). New Jersey: ‘N. J.?’ (1, USNM). New York: ‘N. Y.’ (3, USNM). Pennsylvania: Philadelphia, July 1928 (1, CNC). Rhode Island: ‘R. I.’ (6, USNM). Vermont: White River Junction, 29.V.1979, E.J. Kiteley (4, CNC).

##### Diagnosis.

This species can be difficult to distinguish from *L.sparsellum*. *Lathrobiumsimile* has elongate antennomeres, whereas *L.sparsellum* has subquadrate antennomeres. Additionally, *L.simile* has a marginally thicker neck. Males can be further distinguished by the emargination on sternite VIII. The emargination of *L.simile* is wider than deep and there is a patch of thick black setae below. The emargination of *L.sparsellum* is deeper than wide and there are no thick black setae. Females are easily distinguished by the shape of the proctiger, which is conical in *L.simile* but pointed in *L.sparsellum*, a unique characteristic of that species.

##### Description.

Body length 8 mm; body coloration red, appendages light yellow. Gular sutures parallel, widely separate; antennomeres V–VII longer than wide. Elytra as long as pronotum. Females with paraprocts divided; sternite VIII oblong. Male sternite VIII with wide, shallow emargination and two vertical rows of thick black setae (Fig. 49B). Genitalia as in Fig. 49.

##### Distribution.

Canada: MB, NB, NS, ON, QC (Bousquet et al. 2013). USA: CT, DC, IN, MA, ME, NH, NJ, NY, **PA**, RI, **VT** (Newton 2022).

#### ﻿ Lathrobium (Lathrobium) sparsellum

Taxon classificationAnimaliaColeopteraStaphylinidae

Casey, 1905

3A46C9E9-4FE0-573E-84A9-3DA766AAC7A8

Lathrobium sparsellum Casey, 1905: 87.
Lathrobium obtusum Casey, 1905: 86. New synonym.
Lathrobium postremum Casey, 1905: 88. New synonym.
Lathrobium rigidum Casey, 1905: 88. New synonym.

##### Type material.

***Lectotype***, *Lathrobiumsparsellum* Casey, herein designated (USNM): “Winnipg. Man. / CASEY bequest 1925 / [red] TYPE USNM 38120 / [handwritten] *sparsellum* / Lectotype *Lathrobiumsparsellum* Casey Desg. Haberski & Caterino.” ***Lectotype***, *Lathrobiumobtusum* Casey, herein designated (USNM): “Mass/ CASEY bequest 1925 / [red] TYPE USNM 38119 / Lectotype *Lathrobiumobtusum* Casey Desg. Haberski & Caterino.” ***Lectotype***, *Lathrobiumpostremum* Casey, herein designated (USNM): “R.I. / CASEY bequest 1925 / [red] TYPE USNM 38123 / [handwritten] *postremum* / Lectotype *Lathrobiumpostremum* Casey Desg. Haberski & Caterino.” ***Lectotype***, *Lathrobiumrigidum* Casey, herein designated (USNM): “R.I. / CASEY bequest 1925 / [red] TYPE USNM 38122 / [handwritten] *rigidum* / Lectotype *Lathrobiumrigidum* Casey Desg. Haberski & Caterino.”

##### Other material.

Canada: ‘Can’ (1, USNM). Quebec: MRC des Deux-Montagnes: Parc National d’Oka, 06 May 2023, R. Vigneault, handpicked near beach (1, NBC); same data, except white tulle interception trap in composting site (1, NBC). USA; Connecticut: New Haven Co.: New Haven (1, USNM). Massachusetts: ‘Mass’ (2, USNM). New Hampshire: Strafford Co.: Spruce Hole Conservation Area, D. S. Chandler (1, UNHC); Rhode Island: ‘R. I.’ (4, USNM).

##### Diagnosis.

This species closely resembles *L.simile* but can be differentiated as described under the diagnosis for *L.simile*.

##### Description.

**Figures 50–53.**
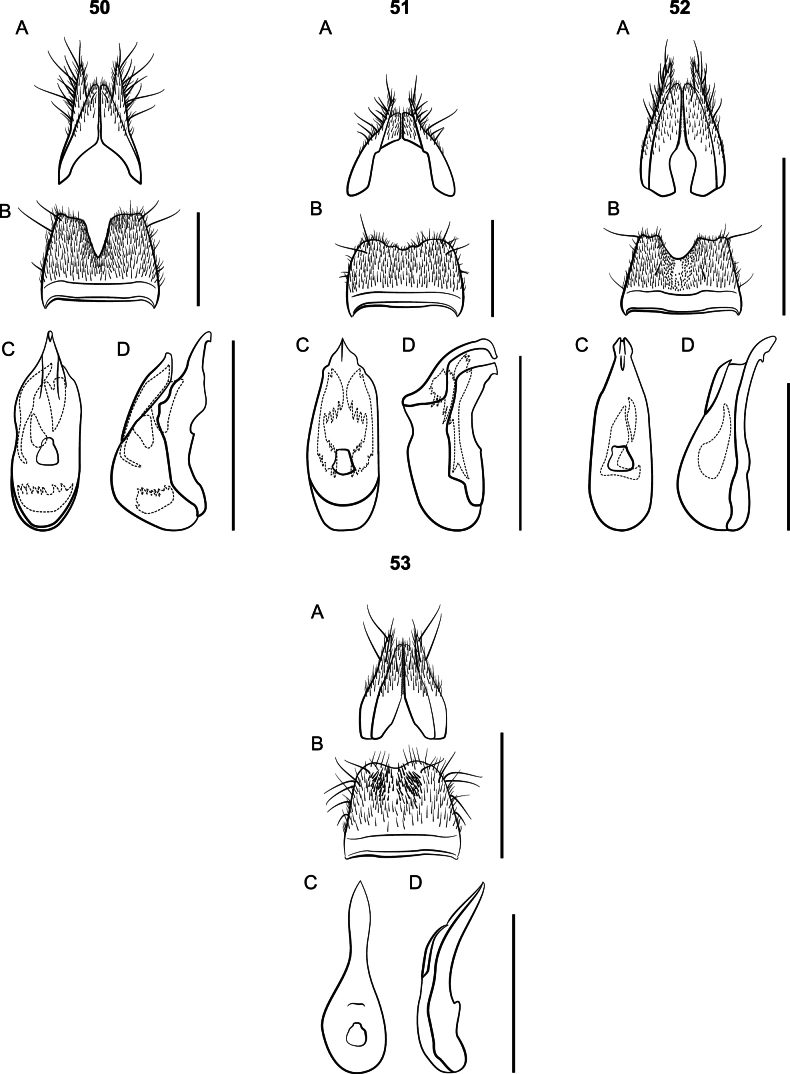
*Lathrobium* terminalia **A** female terminalia **B** male sternite VIII **C** aedeagus in ventral view **D** aedeagus in lateral view. **50***L.sparsellum***51***L.spissicorne***52***L.washingtoni***53***L.debile*. Scale bars: 1 mm.

Body length 8 mm; body coloration dark, appendages lighter red. Gular sutures parallel, widely separate; antennomeres V–VII as long as wide. Elytra as long as pronotum. Females with paraprocts divided; proctiger narrow; sternite VIII weakly oblong. Male sternite VIII with narrow, deep emargination. Genitalia as in Fig. 50.

##### Distribution.

Canada: AB, MB, NB, NF, NS, ON, QC (Bousquet et al. 2013). USA: **CT**, MA, ME, NH, NY, RI, VT (Newton 2022).

##### Remarks.

We reduce *Lathrobiumobtusum*, *Lathrobiumpostremum*, and *Lathrobiumrigidum* to synonymy with *Lathrobiumsparsellum* based on the inaccuracies of the original descriptions and the inability to find alternative distinguishing characters. *Lathrobiumpostremum* and *L.rigidum* were differentiated from *L.sparsellum* based on their elytra being shorter than the pronotum, as opposed to subequal as in *L.sparsellum*. However, we measured Casey’s specimens and found the elytra to be subequal or slightly longer than the pronotum in all three species.

*Lathrobiumobtusum* was distinguished from *L.sparsellum* primarily based on the gular sutures being “moderately separated and feebly converging” as opposed to “widely separated, almost straight” in *L.sparsellum* (Casey 1905). However, we could not see a difference in suture shape, and sutures in both species were approximately equidistant. We examined the genitalia of all four species and found no morphological differences in males or females.

#### ﻿ Lathrobium (Lathrobium) spissicorne

Taxon classificationAnimaliaColeopteraStaphylinidae

Casey, 1905

8FBDA31F-41E4-5AF1-B48C-83A647A62A3C

Lathrobium spissicorne Casey, 1905: 83.

##### Type material.

***Lectotype****Lathrobiumspissicorne* Casey, herein designated (USNM): “Mass / CASEY bequest 1925 / [red] TYPE USNM 38111 / [handwritten] *spissicorne* / Lectotype *Lathrobiumspissicorne* Casey Desg. Haberski & Caterino.”

##### Other material.

USA: Massachusetts: ‘Mass’ (1, USNM); ‘Mass’ (1, MCZ). Michigan: Wayne Co.: Detroit, Oct (1, USNM).

##### Diagnosis.

*Lathrobiumspissicorne* can be distinguished from all other Nearctic *Lathrobium* by unique primary and secondary sexual characters. The male sternite VIII has a scalloped emargination, and the dorsal plate of the aedeagus bends over the top of the ventral process. In females, the inner margin of the gonocoxites is broad and distinctively arched.

##### Description.

Body length 7 mm; body coloration dark red throughout. Gular sutures parallel, widely separate; antennomeres V–VII as wide as long. Elytra longer than pronotum. Female sternite VIII weakly oblong. Genitalia as in Fig. 51.

##### Distribution.

Canada: NB, ON, QC, PE (Bousquet et al. 2013). USA: MA, MI.

#### ﻿ Lathrobium (Lathrobium) washingtoni

Taxon classificationAnimaliaColeopteraStaphylinidae

Casey, 1905

9A5AC410-1697-58C9-B8C3-B98538437C05

Lathrobium washingtoni Casey, 1905: 87.
Lathrobium illini Casey, 1905: 86. New synonym.
Lathrobium longiventre Casey, 1905: 85. New synonym.
Lathrobium picescens Casey, 1905: 85. New synonym.
Lathrobium vancouveri Casey, 1905: 86. New synonym.

##### Type material.

***Lectotype***, *Lathrobiumwashingtoni* Casey, herein designated (USNM): “N. H. / ♂ / [red] TYPE USNM 38121 / CASEY bequest 1925 / [handwritten] *washingtoni* / Lectotype *Lathrobiumwashingtoni* Casey Desg. Haberski & Caterino.” ***Lectotype***, *Lathrobiumillini* Casey, herein designated (USNM): “Illinois / CASEY bequest 1925 / [handwritten] *illini* / [red] TYPE USNM 38118 / Lectotype *Lathrobiumillini* Casey Desg. Haberski & Caterino.” ***Lectotype***, *Lathrobiumlongiventre* Casey, herein designated (USNM): “Bayfld / CASEY bequest 1925 / [handwritten] *longiventre* / [red] TYPE USNM 38115 / Lectotype *Lathrobiumlongiventre* Casey Desg. Haberski & Caterino.” ***Lectotype***, *Lathrobiumpicescens* Casey, herein designated (USNM): “N.H. / CASEY bequest 1925 / [handwritten] *picescens* / [red] TYPE USNM 38116 / Lectotype *Lathrobiumpicescens* Casey Desg. Haberski & Caterino.” ***Lectotype***, *Lathrobiumvancouveri* Casey, herein designated (USNM): “Br.C / CASEY bequest 1925 / [handwritten] *vancouveri* / [red] TYPE USNM 38117 / Lectotype *Lathrobiumvancouveri* Casey Desg. Haberski & Caterino.”

##### Other material.

Canada: British Columbia: Terrace, Hippisley (1, MCZ). Newfoundland: ‘N’f’land’ 19–24 Jul 1907 (1, MCZ). USA: Alaska: Fairbanks North Star Borough: Nome Creek, 11 Jun 2018, R. Nowicki (1, UAM). Minnesota: ‘Min.’ (2, USNM). New Hampshire: ‘N. H.’ (1, USNM); Coos Co.: Mount Washington (1, MCZ); Grafton Co.: Woodstock, White Mountains (1, USNM). Wisconsin, Bayfield Co. Bayfield (1, USNM).

##### Diagnosis.

This species is similar in appearance to *L.leconteanum* and can be differentiated as discussed in the diagnosis of *L.leconteanum*.

##### Description.

Body length 6–7 mm; body coloration dark red, appendages lighter red. Gular sutures, widely separate, converging slightly posteriorly; antennomeres V–VIII as wide as long. Elytra as long as pronotum. Females with paraprocts divided; sternite VIII strongly oblong, tip 1/3 width of base. Genitalia as in Fig. 52.

##### Distribution.

Canada: AB, BC, MB, NB, NF, NS, NT, ON, QC, SK (Bousquet et al. 2013). USA: AK, ID, IL, **MN**, MT, NH, WI (Newton 2022).

##### Remarks.

We reduce *Lathrobiumillini*, *Lathrobiumlongiventre*, *Lathrobiumpicescens*, and *Lathrobiumvancouveri* to synonymy with *Lathrobiumwashingtoni*, because the original diagnoses were based on a single inaccurate character. All four of the herein synonymized species were distinguished from *L.washingtoni* by having the middle joints of the antennae not longer than wide, as opposed to “elongate” (Casey 1905). We measured the fifth antennomeres of each species and found the ratio of length to width varied within and between species from 1.1 to 1.4× as long as wide, making this character useless for diagnosis. We examined the genitalia and found no differences among species in males or females.

**Subgenus Lathrolepta Casey, 1905: 72.**

#### ﻿ Lathrobium (Lathrolepta) debile

Taxon classificationAnimaliaColeopteraStaphylinidae

LeConte, 1880

C95BDAF8-F825-50B4-BBBE-6F551141CD17

Lathrobium debile LeConte, 1880: 176.
Lathrolepta debilis Casey, 1905: 104.
Lathrobium (Lathrolepta) debilie : Bernhauer and Schubert 1912: 258.

##### Type material.

***Lectotype***, *Lathrobiumdebile* LeConte, herein designated (MCZ): “Mic. / [handwritten] 463 / [handwritten] L.debile Lec. / Lectotype *Lathrobiumdebile* LeConte Desg. Haberski & Caterino.”

##### Other material.

Canada: Ontario: ‘Ont. Can.’ (1, USN). USA: Indiana: ‘In.’ (1, USNM). Massachusetts: ‘Mass,’ Jul 1903, J. Blanchard (1, MCZ); ‘Mass’ (5, USNM). Michigan: ‘Mic.’ (1, USNM). New Hampshire: Grafton Co.: Downes Brook, Potash Mountain, 05 Jun 2021, A. Haberski (1, CUAC); Belknap Co.: Lower Gilmanton, 23 Apr 1982, D. S. Chandler (6, UNHC); Chesire Co.: Rhododendron State Park, 22 Apr 1982, D. S. Chandler, sifted rotten spruce & fir logs (4, UNHC); Hillsborough Co.: Miller State Park, 22 Apr 1982, D. S. Chandler, oak litter (2, UNHC); Strafford Co.: Spruce Hole Conservation Area, 3 mi SW Durham, 02 Jul 1987, litter near bog (2, UNHC). New York: ‘N. Y.’ (1, USNM).

##### Diagnosis.

*Lathrobiumdebile* differs from all other described species of Nearctic *Lathrobium* in its small size and diverging gular sutures. However, it resembles members of the *sibiricum* group, which may have undescribed diversity in Alaska and Northwest Territories. Species in the *sibiricum* group lack a dorsal plate on the aedeagus and have a sclerotized ring-like structure on the membranous endophallus. *Lathrobiumdebile* has a dorsal plate and lacks the ring-like structure.

##### Description.

Extremely small species, body length 3.5–4 mm; coloration reddish, appendages lighter. Gular sutures divergent (Fig. 1D). Antennae short, antennomeres as long as wide. Female sternite VIII oblong. Genitalia as in Fig. 53.

##### Distribution.

Canada: ON, NB, QC (Bousquet et al. 2013). USA: IA, **IN**, MA, MI, NH, NY (Newton 2022).

### ﻿Genus *Pseudolathra* Casey

#### ﻿ Pseudolathra parcum

Taxon classificationAnimaliaColeopteraStaphylinidae

(LeConte, 1880) comb. nov.

77095513-750A-5BF2-86EC-C91265E18E4C

Lathrobium parcum LeConte, 1880: 92.

##### Type material.

***Holotype*** (MCZ): “Capron 15.4. Fla / 741 / [handwritten] *L.parcum* Lec. / [red] Type 6458.”

##### Remarks.

This species is transferred to *Pseudolathra* based on the protibia not being expanded with protibial combs in lateral view (Żyła et al. 2020).

#### ﻿ Pseudolathra texana

Taxon classificationAnimaliaColeopteraStaphylinidae

(Casey, 1905) comb. nov.

129460FD-95B4-5288-A23D-997DC51054EF

Lathrobiopsis texana Casey, 1905: 98.
Lathrobium (Lathrobiopsis) texanum : Bernhauer and Schubert 1912: 269.

##### Type material.

***Lectotype***, *Lathrobiopsistexana* Casey, herein designated (USNM): “Tex. / CASEY bequest 1925 / [red] TYPE USNM 38120 / [handwritten] *Lathrobiopsistexanum Csy* / Lectotype *Lathrobiopsistexana* Casey Desg. Haberski & Caterino.”

##### Remarks.

This species is transferred to *Pseudolathra* based on the protibia not being expanded with protibial combs (Żyła et al. 2020) and the presence of a carina on the elytral epipleuron (Assing and Schülke 2012). Its habitus is overall inconsistent with North American *Lathrobium*. The head is square with the posterior angles projecting; the pronotum is quadrate rather than long; and the elytral punctures are arranged in regular rows.

### ﻿Checklist of the Nearctic species of *Lathrobium* north of Mexico

**Subgenus Abletobium Casey**

1. *Lathrobiumabsconditum* Haberski & Caterino, sp. nov.
2. *Lathrobiumbalsamense* Haberski & Caterino, sp. nov.
3. *Lathrobiumhardeni* Haberski & Caterino, sp. nov.
4. *Lathrobiumlapidum* Haberski & Caterino, sp. nov.
5. *Lathrobiumpallescens* (Casey, 1905)
6. *Lathrobiumshermani* Fall, 1917
7. *Lathrobiumsmokiense* Haberski & Caterino, sp. nov.
8. *Lathrobiumsolum* Haberski & Caterino, sp. nov.
9. *Lathrobiumthompsonorum* Haberski & Caterino, sp. nov.

**Subgenus Apteralium Casey**

1. *Lathrobiumbrevipenne* LeConte, 1863
2. *Lathrobiumcamplyacra* Haberski & Caterino, sp. nov.
3. *Lathrobiumcarolinae* (Casey, 1905)

**Subgenus Lathrobioma Casey**

1. *Lathrobiumdivisum* LeConte, 1880
2. *Lathrobiumnanulum* (Casey, 1905)
3. *Lathrobiumothioides* LeConte, 1880
4. *Lathrobiumscolopaceum* (Casey, 1905)
5. *Lathrobiumtenue* LeConte, 1863

**Subgenus Lathrobium s. str.**

1. *Lathrobiumamplipenne* Casey, 1905
2. *Lathrobiumarmatum* Say, 1830
3. *Lathrobiumconfusum* LeConte, 1880
4. *Lathrobiumcrurale* (Casey, 1905)
5. *Lathrobiumfauveli* Duvivier, 1883
6. *Lathrobiumfulvipenne* (Gravenhorst, 1806)
7. *Lathrobiumgeminum* Kraatz, 1857
8. *Lathrobiuminsanum* Blatchley, 19108.
9. *Lathrobiumislae* Haberski & Caterino, sp. nov.
10. *Lathrobiumleconteanum* Scheerpeltz, 1933
11. *Lathrobiumlineatocolle* Scriba, 1859
12. *Lathrobiumlintneri* Notman, 1921
13. *Lathrobiumlividum* Haberski & Caterino, sp. nov.
14. *Lathrobiumpedale* LeConte, 1863
15. *Lathrobiumpraelongum* Casey, 1905
16. *Lathrobiumpuncticolle* Kirby, 1837
17. *Lathrobiumrhodeanum* (Casey, 1905)
18. *Lathrobiumsimile* LeConte, 1863
19. *Lathrobiumsparsellum* Casey, 1905
20. *Lathrobiumspissicorne* Casey, 1905
21. *Lathrobiumsubgracile* (Casey, 1905)
22. *Lathrobiumtenebrosum* Notman, 1919
23. *Lathrobiumwashingtoni* Casey, 1905

**Subgenus Lathrolepta Casey**

1. *Lathrobiumdebile* LeConte, 1880

The MCZ contains labelled holotypes for two additional species from California, “*Lathrobiumatrapubes* Watrous, 1983” and “*Lathrobiumsubcarinatum* Watrous, 1983”. The CNC contains an un-named holotype of an undescribed species near *Lathrobiumsibiricum* Fauvel, 1875 from Arctic Alaska. These have not yet been properly described, though we have confirmed that they are members of *Lathrobium* and morphologically distinct from other species.

### ﻿Key to North American *Lathrobium* north of Mexico

**Table:** 

1	Eyes reduced to white membrane completely lacking ommatidia	**2**
–	Eyes present with distinct ommatidia	**9**
2	Antennomeres V and VI not longer than wide	**3**
–	Antennomeres V and VI longer than wide	**6**
3	Smaller species, body length ~ 6 mm. Male sternite VIII with V-shaped emargination and without thick black setae. Female with apical lobes of paraproct shorter than continuous basal portion in dorsal view; sternite VIII conical. Restricted to spruce-fir forests above 1500 m elevation in the Southern Appalachians	**4**
–	Larger species, body length > 8 mm. Male sternite VIII with a broad and shallow emargination, with or without thick black setae; aedeagus with triangular ventral process in lateral view (Figs 17F, 21D). Female, where known, with apical lobes of paraproct longer than continuous basal portion in dorsal view; sternite VIII oblong. Found at lower elevations	**5**
4	Aedeagus tubular (Fig. 9E, F)	***Lathrobiumsmokiense***
–	Aedeagus not tubular (Fig. 10D, E)	***Lathrobiumbalsamense***
5	Male sternite VIII with three transverse combs of thick black setae (Fig. 17D); aedeagus with dorsal plate ½ length of ventral process, ventral process strongly asymmetrical in ventral view (Fig. 17E, F)	***Lathrobiumlapidum***
–	Male sternite VIII without thick black setae (Fig. 21B); aedeagus with dorsal plate 1/3 length of ventral process, ventral process weakly asymmetrical in ventral view (Fig. 21C, D)	***Lathrobiumsolum***
6	Large species, body length ~ 9 mm. Male sternite VIII without transverse combs of thick black setae (Fig. 11D). Female gonocoxites with pubescent lobes (Fig. 11B)	***Lathrobiumabsconditum***
–	Smaller species, body length < 9 mm. Male sternite VIII with transverse combs of thick black setae (Figs 14D, 20B). Female gonocoxites without lobe-like projections (Figs 20B, 22A)	**7**
7	Male sternite VIII with two combs of 5–8 thick black setae to either side of midline (Fig. 20B). Female gonocoxites with fine pubescence (Fig. 20A); subgenital plate absent	***Lathrobiumshermani***
–	Male sternite VIII with three combs of thick black setae (Fig. 14D). Female with either valvifers and coxites divided, or with subgenital plate	**8**
8	Male sternite VIII with V-shaped emargination (Fig. 22D). Female sternite VIII without apical notch; sternum IX with valvifers and coxites divided (Fig. 22B); no subgenital plate; apical lobes of paraproct longer than continuous basal portion in dorsal view	***Lathrobiumthompsonorum***
–	Male sternite VIII without emargination (Fig. 14D). Female sternite VIII with apical notch; sternite IX with valvifers and coxites fused; subgenital plate present (Fig. 14B); apical lobes of paraproct shorter than continuous basal portion in dorsal view	***Lathrobiumhardeni***
9	Elytra distinctly shorter than pronotum	**10**
–	Elytra longer than, or equal to pronotum	**15**
10	Eyes small, ~ 30 ommatidia. Body pale. Head wider than elytra	***Lathrobiumpallescens***
–	Eyes larger. Body black or reddish. Head width subequal to elytra	**11**
11	Small species, body length < 5 mm; coloration black. Gular sutures arcuate (Fig. 1C). Restricted to spruce-fir forests > 1500 m elevation in the southern Appalachians	**12**
–	Larger species, body length > 8 mm; coloration reddish. Gular sutures straight (Fig. 1A). Restricted to hardwood forest < 1500 m elevation in the southern Appalachians or the Interior Highlands	**13**
12	Spine of internal sac of aedeagus long with ball-shaped tip (Fig. 7F). Female gonocoxites narrow at base (Fig. 7B)	***Lathrobiumislae***
–	Spine of internal sac of aedeagus short, with curved spine projecting above ventral process (Fig. 8E). Female gonocoxites not narrowed at base (Fig. 8A)	***Lathrobiumlividum***
13	Male sternite VIII with two projecting lobes (Fig. 24B); aedeagus variable (Figs 24–26C, D). Female sternite VIII oblong. Endemic to the Interior Highlands of the United States	***Lathrobiumbrevipenne***
–	Male sternite VIII without projections (Fig. 5D); aedeagus with median lobe fully sclerotized. Female sternite VIII conical (Fig. 5C). Endemic to the Southern Appalachian Mountains	**14**
14	Aedeagus with ventral process straight in lateral view (Fig. 4e). Female subgenital plate absent (Fig. 4A)	***Lathrobiumcarolinae***
–	Aedeagus with ventral process curved, tip ending below median foreman in lateral view (Fig. 5F). Female with subgenital plate present as small, lightly sclerotized chevron (Fig. 5B)	***Lathrobiumcamplyacra***
15	Metatarsi compact, tarsomeres I–IV subequal in length. Small species, body length ≤ 6 mm (except *L.divisum*)	**16**
–	Metatarsi elongate, tarsomere II longer than III or IV. Body length ≥ 6mm	**22**
16	Very small species, body length < 4 mm. Gular sutures divergent (Fig. 1D)	***Lathrobiumdebile***
–	Body length > 4mm. Gular sutures arcuate or parallel (Fig. 1A, C)	**17**
17	Antennomeres V and VI longer than wide. Male sternite VIII without thick black setae (Fig. 36B)	***Lathrobiumconfusum***
–	Antennomeres V and VI not longer than wide. Male sternite VIII with apical thick black setae (Figs 29–33B)	**18**
18	Large species, body length 8 mm. Male sternite VIII without emargination (Fig. 29B). Female with apical lobes of paraproct as long as continuous basal portion in dorsal view	***Lathrobiumdivisum***
–	Small species, body length 6 mm. Male sternite VIII with round emargination (Figs 30–33B). Female with apical lobes of paraproct shorter than continuous basal portion in dorsal view, or paraprocts divided	**19**
19	Aedeagus narrow, apices of dorsal plate and ventral process with large apical tooth (Fig. 30C, D). Female with valvifers and coxites divided (Fig. 30A)	***Lathrobiumnanulum***
–	Aedeagus wide and scoop-shaped in ventral view (Figs 30C, 31–33C). Female with valvifers and coxites fused (Fig. 31A)	**20**
20	Ventral process of aedeagus with apex downturned in lateral view with scalloped indentation on ventral surface (Fig. 32C, D). Female with paraprocts divided (Fig. 2A)	***Lathrobiumscolopaceum***
–	Ventral process of aedeagus broadly rounded or with horn-like projections (Figs 31C, 33C). Female with paraprocts continuous (Fig. 2B)	**21**
21	Ventral process of aedeagus with two horn-like projections (Fig. 31C). Females with median edge of gonocoxites broad and sinuate (Fig. 31A)	***Lathrobiumothioides***
–	Ventral process of aedeagus broadly rounded (Fig. 33C). Females with median edge of gonocoxites narrow and concave (Fig. 33A)	***Lathrobiumtenue***
22	Gular sutures converging, often nearly touching posteriorly (Fig. 1B). Large species, body length ≥9 mm. Elytra often bicolored	**23**
–	Gular sutures straight or slightly convergent but widely separated throughout (Fig. 1A). Smaller species, body length ≤ 8 mm. Elytra rarely bicolored	**26**
23	Antennomeres V and VI longer than wide	**24**
–	Antennomeres V and VI as long as wide	**25**
24	Male sternite VIII with three small emarginations (Fig. 34B), ventral process of aedeagus with oblique bend (Fig. 34D). Female gonocoxites as long as tergite IX (Fig. 34A)	***Lathrobiumamplipenne***
–	Male sternite VIII with a single medial emargination (Fig. 37B), ventral process of aedeagus with 90-degree bend (Fig. 40D). Female gonocoxites ½ as long as tergite IX (Fig. 40A)	***Lathrobiumgeminum***
25	Male sternite VIII with large emargination, 1/4 depth of sternite (Fig. 46B); aedeagus with apex of ventral process broad and flat (Fig. 46C). Female with valvifers and gonocoxites divided (Fig. 46A); apical lobes of paraproct as long as basal portion	***Lathrobiumpedale***
–	Male sternite VIII with small emargination, 1/10 depth of sternite (Figs 35B, 47B); aedeagus with apex of ventral process pointed. Female with valvifers and gonocoxites fused (Fig. 35A); apical lobes of paraproct shorter than basal portion	**26**
26	Dorsal plate of aedeagus with large, back-curving hook-like projection (Fig. 35D). Female sternite VIII conical; interior edge of gonocoxites convex (Fig. 35A)	***Lathrobiumarmatum***
–	Aedeagus with apex of ventral process narrow and as long as median lobe (Fig. 47C). Female sternite VIII oblong; interior edge of gonocoxites concave (Fig. 47A)	***Lathrobiumpraelongum***
27	Antennomeres V and VI as long as wide	**28**
–	Antennomeres V and VI more than 1.5× longer than wide	**29**
28	Male sternite VIII with deep emargination, 1/3 depth of sternite (Fig. 52B). Aedeagus with dorsal plate relatively short (Fig. 52D). Female with paraprocts divided (Fig. 2A)	***Lathrobiumwashingtoni***
–	Male sternite VIII with shallow emargination, 1/8 depth of sternite (Fig. 51B). Aedeagus with dorsal plate longer (Fig. 51D). Female with paraprocts continuous (Fig. 2B)	***Lathrobiumspissicorne***
29	Male sternite VIII with a shallow emargination, < 1/8 depth of sternite (Figs 38B, 48B). Female with valvifers and gonocoxites divided (Fig. 38A)	**30**
–	Male sternite VIII with a deep emargination, > 1/5 depth of sternite (Figs 37B, 50B). Female with valvifers and gonocoxites fused (where known)	**33**
30	Ventral process of aedeagus more-or-less straight, tip level with foreman in lateral view, excluding apical projection (Figs 38D, 48D). Female tergite IX with apical lobes of paraprocts longer than continuous anterior portion in dorsal view (Fig. 2B)	**31**
–	Ventral process of aedeagus angled ventrally, tip ending below median foreman in lateral view (Figs 39D, 45D). Female tergite IX with apical lobes of paraprocts longer than continuous anterior portion in dorsal view (Fig. 2B)	**32**
31	Male sternite VIII longer than wide (Fig. 48B). Female sternite VIII oblong; valvifers and coxites subequal in length (Fig. 48A); apical lobes of paraproct more than twice as long as basal portion	***Lathrobiumrhodeanum***
–	Male sternite VIII as long as wide (Fig. 38B). Female sternite VIII conical; coxites shorter than valvifers (Fig. 38A); apical lobes of paraproct 1.5× longer than basal portion	***Lathrobiumfauveli***
32	Ventral process of aedeagus considerably asymmetrical in ventral view (Fig. 39C). Female sclerite VIII oblong	***Lathrobiumfulvipenne***
–	Ventral process of aedeagus symmetrical in ventral view (Fig. 45C). Female sclerite VIII conical with truncate apex	***Lathrobiumlineatocolle***
33	Female with paraprocts undivided (Fig. 2B, C). Male sternite VIII without thick black setae, emargination 1/5 depth of sternite (Fig. 37B)	***Lathrobiumcrurale***
–	Female with paraprocts divided (Fig. 2A). Male sternite VIII with thick black setae along medial line (Figs 44B, 49B), or emargination ½ depth of sternite (Fig. 50B)	**34**
34	Male sternite VIII without thick black setae, emargination deeper than wide, ½ depth of sternite (Fig. 50B). Female with gonocoxites narrow and glabrous at base (Fig. 50A); proctiger pointed	***Lathrobiumsparsellum***
–	Male sternite VIII with thick black setae medially, emargination < 1/2 depth of sternite (Fig. 44B, 49B). Female gonocoxites wide at base (Fig. 50A), or with fine pubescence (Fig. 44A); proctiger conical	**35**
35	Ventral process of aedeagus with apex divided (Fig. 44). Female gonocoxites with pubescent lobes (Fig. 44A); proctiger narrow	***Lathrobiumleconteanum***
–	Ventral process of aedeagus with apex not divided (Fig. 49C). Female gonocoxites glabrous at base, without pubescent lobes (Fig. 49A); proctiger conical	***Lathrobiumsimile***

## ﻿Discussion

After the taxonomic changes made in this paper, there are 41 valid species of Nearctic *Lathrobium*, and three as yet undescribed species. Forty-four species is assuredly an underestimate of the true diversity. The subterranean fauna is poorly documented and a relatively modest effort by a few collectors discovered five of the new species described here. Buried pipe traps are an efficient and inexpensive method for collecting hypogean beetles, but their use has so far been limited, and a wider deployment is likely to reveal more undescribed species. We have already seen morphologically distinct specimens from Illinois and North Carolina, but further material is needed to warrant their descriptions.

We used a combined morphological and molecular approach to test the hypothesis of cryptic speciation among three flightless lineages of Appalachian *Lathrobium*. They have accumulated genotypic and phenotypic variations at different rates making it difficult to delimit species based on a single character system. We obtained incongruent results in the *L.smokiense–balsamense* and *L.islae–lividum* lineages. In the case of *L.islae–lividum*, only mPTP failed to split the lineage into at least two putative species, and the paraphyly in our tree was consistent with some scenarios of recent speciation (Bush and Butlin 2004).

The results for *L.smokiense–balsamense* were more ambiguous, but we felt the morphological differences justified species designation. Our molecular methods were based on a single mitochondrial locus, COI. Gene-tree/species-tree discordance is therefore a potential source of error in our phylogenetic and mPTP analyses (e.g., Maddison 1997). Furthermore, uniparental inheritance and smaller effective population size in mtDNA can cause mito-nuclear discordance in biogeographic signals, so interpreting COI alone might present an incomplete picture of *Lathrobium* evolution (Toews and Brelsford 2012). Morphology avoids these complications because it is influenced by multiple genes. Our morphological dataset had the additional advantage of a larger sample size with a broader geographic scope. However, we recognize that there might be one or more additional cryptic species that we cannot adequately defend with the current data.

The *Lathrobiumsmokiense–balsamense* and *L.islae–lividum* lineages are endemic to isolated sky-islands of red spruce-Fraser fir forest on peaks above 1700 m elevation. Linear distance between islands did not correlate with genetic distance. The highest pairwise genetic distances all occurred between neighboring localities, Grandfather Mountain and Roan High Knob (33 km), Clingmans Dome and Mount Kephart (8 km), and Mt. Lyn Lowry and Richland Balsam Mountain (25 km). The lack of isolation by distance effects and lack of strong population structure are consistent with a hypothesis of range expansion in the recent past (Crespi et al. 2003), possibly when spruce-fir forest was more contiguous ~ 10,000 ya (Delcourt and Delcourt 1998).

Polyphyly of *L.smokiensis* and *L.balsamensis* may indicate speciation was more recent than in *L.islae–lividum*, or it may be indicative of recent secondary contact. Linear distances between spruce-fir islands south of the French Broad River basin are shorter than to the north. Forest patches may therefore have come into contact more often or stayed connected longer.

The southern Appalachian red spruce-Fraser fir forest is among the most endangered forest types in the United States (Noss and Scott 1995; Wear and Greis 2002), and the above-mentioned species are endemic to a rare bryophyte mat microhabitat within it. They share this microhabitat with the spruce-fir moss spider, *Microhexuramontivaga*, which was added to the Federal List of Endangered and Threatened Wildlife and Plants by the USFWS in 1994 due to low abundance and significant threats to its remaining habitat (Fridell 1995). A conservation assessment of *Lathrobium* populations is unlikely, but they may benefit from the habitat protection already afforded to *M.montivaga*.

The deep divergence in the *L.carolinae–camplyacra* lineage implies ancient vicariance, and the lack of genetic diversity within the two species suggests a recent population bottleneck. Unlike spruce-fir species, *L.carolinae* and *L.camplyacra* might have experienced a recent range contraction. The expansion of spruce-fir into lower elevations during the last glacial maximum might have pushed northern hardwoods into small refugia, causing a bottleneck.

River basins appear to be the primary barriers to gene flow in the region. The French Broad River basin is a well-documented barrier implicated in numerous taxa (Crespi et al. 2003; Thomas and Hedin 2008; Caterino and Langton-Myers 2019), and none of the flightless *Lathrobium* have been able to disperse across it. The Little Tennessee River basin, which largely separates *L.camplyacra* and *L.carolinae*, acts as an impediment to gene flow but not an absolute physical barrier. *Lathrobiumcarolinae* occurs east of the basin along the Great Smoky Mountains, but apparently there has not been enough time, or insufficient isolation, to observe genetic variation in COI between populations east and west of the river, at least as sampled. A similar pattern has been observed in *Dasycerus* staphylinids (Caterino and Harden 2024) and *Sabacon* harvestmen (Hedin and McCormack 2017). Hedin and McCormack (2017) hypothesized that reduced gene flow across the river basin combined with competitive exclusion or low hybrid fitness maintains lineage boundaries.

### ﻿Subgeneric placement of microphthalmous Lathrobium

The depigmented, microphthalmous, and flightless *Abletobium* bears a strong resemblance to the Palearctic subgenus Glyptomerus, as noted by Casey (1905) and Fall (1917). These characters are common in hypogean and troglobitic taxa across Arthropoda, so it is unclear how the two are related or if they are synonyms. The addition of seven new Nearctic *Abletobium* allowed us to reassess characters that may be phylogenetically relevant to this question.

Three characters, in addition to the general habitus, have been proposed as synapomorphies for *Glyptomerus* (Coiffait 1982): 1) dorsal plate and ventral process of the aedeagus without teeth, except sometimes at the apex; 2) median lobe of the aedeagus always divided in two at the apex; and 3) male sternite VIII with transverse combs of thick black setae. However, these characters are variable within, or not exclusive to *Glyptomerus* (Bordoni 2020, 2018, 2012). Among Nearctic *Lathrobium* the first character is not exclusive to the “glyptomeroid” habitus, and in fact applies to most species. The second character does not occur in any Nearctic glyptomeroids. Only the third character, combs of black setae, is exclusive to glyptomeroids, but is present in only half the species (*L.lapidum*, *L.thompsonorum*, *L.hardeni*, and *L.shermani*).

Casey (1905) erected *Abletobium* as a monospecific genus for *L.pallescens* and distinguished it from *Glyptomerus* based on a single character: *Abletobium* had eyes that were small, with distinct ommatidia, whereas *Glyptomerus* had eyes reduced to white scars. It also lacks characters two and three listed above. Fall (1917) added *L.shermani* to the subgenus, which does not have eyes but does have combs of black setae on sternite VIII. He noted its resemblance to *Glyptomerus* but determined *Abletobium* was a better fit. To further cloud the matter, several Palearctic *Glyptomerus* (e.g., *Lathrobiumzoiai* Briganti, 1980) have setal combs and eyes resembling *L.pallescens*. The lack of stemmata in Nearctic larvae might distinguish the two subgenera, but too few larvae have been described to be certain. We placed new species in *Abletobium* until the matter can be further tested.

Our COI phylogeny did not include any representatives of *Glyptomerus* and was inadequate to evaluate subgeneric boundaries. DNA is the most promising avenue for unraveling relationships among morphologically convergent taxa. A more complete phylogeny is needed.

### ﻿Larval morphology

Thirteen diagnostic characters were found by Staniec and Bordoni (2022) to distinguish mature (2^nd^ instar) larvae of *Lathrobium* from the well-known larvae of closely related genera: 1) nasale with median tooth; 2) head at least 3× as wide as neck; 3) apotome at most reaching tentorial pits; 4) antennal sensory appendage > ½ length of antennomere IV; 5) mandibular seta L1 residual; 6) stipes quadrate in outline with four setae (one tiny), cuticular process near mala and microstructure near trichobothrium; 7) mala > ½ length of maxillary palpomere I; 8) hypopharynx with microtrichia in central area forming inverted triangle in outline; 9) prementum extended anteriorly; 10) labial palpomere II more or less bent inside; 11) both halves of sternum of thoracic segment I almost touch each other along middle line; 12) prosternum strongly developed, divided into two sternites by narrow, membranous area of width at most 1/10 that of sternite; 13) article I of urogomphi at least 1.5× as long as abdominal segment X (pygopod).

The Nearctic larvae we described conform to all of these characters, except number 2. *Lathrobiumislae* and *L.carolinae* had necks slightly wider than 1/3 the width of the head, though within rounding error. Character 13 could not be judged in *L.carolinae* or *L.islae* because the urogomphi were broken off.

Paederines have only two larval instars, but distinguishing between them is difficult without a side-by-side comparison. We were able to compare 1^st^ and 2^nd^ instars for *L.islae* and *L.hardeni* and found the following differences: body length increased ~ 2× and 1.75× respectively; antennae, maxilla, and labial palpomeres became more elongate; stipes of *L.hardeni* changed from quadrate to elongate; median tooth of the nasale became larger and more sagittate; antennomere IV became club-shaped; and labial palpomere II acquired its characteristic inward bend. These characters might make it easier to age larvae.

Second instar larvae have now been described for 14 species of *Lathrobium*: *Lathrobiumalzonai* Capra & Binaghi, 1938, *Lathrobiumbrunnipes* (Fabricius, 1792), *Lathrobiumcavicola* (H. Müller, 1856), *Lathrobiumfreyi* Koch, 1938, *Lathrobiumelongatum* (Linnaeus, 1767), *Lathrobiumfulvipenne* (Gravenhorst, 1806), *Lathrobiumgeminum* Kraatz, 1857, *Lathrobiumlineatocolle* Scriba, 1859, *L.absconditum*, *L.carolinae*, *L.hardeni*, *L.islae*, and *L.thompsonorum*. This is a small fraction of worldwide *Lathrobium*, so it difficult to know the extent of intraspecific and interspecific variability. Nevertheless, Staniec and Bordoni (2022) summarized 25 variable characters that might be useful for species diagnosis. We here review those characters variable in Nearctic larvae: 1) body length (5–7 mm); 2) presence or absence of stemmata; 3) head shape (triangular, U-shaped, or quadrate); 4) width ratio of neck and head (2.5:1–3.6:1); 5) shape of the median tooth of the nasale (round, emarginate, trifurcate); 6) apotome reaching or not reaching tentorial pits; 7) length ratio of antennomeres; 8) inner margin of mandibles serrate or smooth; 9) mandibles serrate in apical, middle, or basal 1/3; 10) shape of stipes (1.5× to 2.8× as long as wide); 11) length ratio of maxillary palpomeres; 12) strip separating ligula from prementum strongly or poorly sclerotized; 13) abdominal sclerites poorly or strongly sclerotized. Characters 2, 8, 9, and 13 are new, although 8 was noted by previous authors (Staniec et al. 2014), bringing the total number of potentially informative characters to 29. Characters 2, 3, 4, and 13 differentiate eyed species, *L.carolinae* and *L.islae*, from eyeless *Abletobium* larvae.

## Supplementary Material

XML Treatment for Lathrobium (Abletobium) absconditum

XML Treatment for Lathrobium (Abletobium) balsamense

XML Treatment for Lathrobium (Abletobium) hardeni

XML Treatment for Lathrobium (Abletobium) lapidum

XML Treatment for Lathrobium (Abletobium) pallescens

XML Treatment for Lathrobium (Abletobium) shermani

XML Treatment for Lathrobium (Abletobium) smokiense

XML Treatment for Lathrobium (Abletobium) solum

XML Treatment for Lathrobium (Abletobium) thompsonorum

XML Treatment for Lathrobium (Apteralium) brevipenne

XML Treatment for Lathrobium (Apteralium) camplyacra

XML Treatment for Lathrobium (Apteralium) carolinae

XML Treatment for Lathrobium (Lathrobioma) divisum

XML Treatment for Lathrobium (Lathrobioma) nanulum

XML Treatment for Lathrobium (Lathrobioma) othioides

XML Treatment for Lathrobium (Lathrobioma) scolopaceum

XML Treatment for Lathrobium (Lathrobioma) tenue

XML Treatment for Lathrobium (Lathrobium) amplipenne

XML Treatment for Lathrobium (Lathrobium) armatum

XML Treatment for Lathrobium (Lathrobium) confusum

XML Treatment for Lathrobium (Lathrobium) crurale

XML Treatment for Lathrobium (Lathrobium) fauveli

XML Treatment for Lathrobium (Lathrobium) fulvipenne

XML Treatment for Lathrobium (Lathrobium) geminum

XML Treatment for Lathrobium (Lathrobium) islae

XML Treatment for Lathrobium (Lathrobium) leconteanum

XML Treatment for Lathrobium (Lathrobium) lineatocolle

XML Treatment for Lathrobium (Lathrobium) lividum

XML Treatment for Lathrobium (Lathrobium) pedale

XML Treatment for Lathrobium (Lathrobium) praelongum

XML Treatment for Lathrobium (Lathrobium) rhodeanum

XML Treatment for Lathrobium (Lathrobium) simile

XML Treatment for Lathrobium (Lathrobium) sparsellum

XML Treatment for Lathrobium (Lathrobium) spissicorne

XML Treatment for Lathrobium (Lathrobium) washingtoni

XML Treatment for Lathrobium (Lathrolepta) debile

XML Treatment for Pseudolathra parcum

XML Treatment for Pseudolathra texana

## ﻿Additional information

### Author ORCIDs

Adam Haberski https://orcid.org/0000-0002-4062-178X

Michael S. Caterino https://orcid.org/0000-0002-2597-5707

### Data availability

All of the data that support the findings of this study are available in the main text or Supplementary Information.

## Supplementary materials

Supplementary material 1 COI sequence data from Appalachian *Lathrobium* and outgroups Data type txt https://binary.pensoft.net/file/1033589This dataset is made available under the Open Database License (http://opendatacommons.org/licenses/odbl/1.0/). The Open Database License (ODbL) is a license agreement intended to allow users to freely share, modify, and use this Dataset while maintaining this same freedom for others, provided that the original source and author(s) are credited.Adam Haberski, Michael S. Caterino
